# Biomarkers of neurodegeneration across the Global South

**DOI:** 10.1016/S2666-7568(24)00132-6

**Published:** 2024-10-03

**Authors:** Eimear McGlinchey, Claudia Duran-Aniotz, Rufus Akinyemi, Faheem Arshad, Eduardo R Zimmer, Hanna Cho, Boluwatife Adeleye Adewale, Agustin Ibanez

**Affiliations:** Trinity College Dublin, Dublin, Ireland; Global Brain Health Institute, University of California San Francisco (UCSF), San Francisco, CA, USA; Global Brain Health Institute, Trinity College Dublin, Dublin, Ireland; Latin American Brain Health Institute (BrainLat), Universidad Adolfo Ibanez, Santiago de Chile, Chile; Global Brain Health Institute, University of California San Francisco (UCSF), San Francisco, CA, USA; Global Brain Health Institute, Trinity College Dublin, Dublin, Ireland; Neuroscience and Ageing Research Unit, Institute for Advanced Medical Research and Training; Centre for Genomic and Precision Medicine; Global Brain Health Institute, University of California San Francisco (UCSF), San Francisco, CA, USA; Global Brain Health Institute, Trinity College Dublin, Dublin, Ireland; College of Medicine, University of Ibadan, Ibadan, Nigeria; National Institute of Mental Health and Neurosciences (NIMHANS), Bengaluru, India; Department of Pharmacology, Graduate Program in Biological Sciences: Pharmacology and Therapeutics (PPGFT) and Biochemistry (PPGBioq), Universidade Federal do Rio Grande do Sul, Porto Alegre, Brazil; Brain Institute of Rio Grande do Sul, Pontificia Universidade Catolica do Rio Grande do Sul, Porto Alegre, Brazil; McGill Centre for Studies in Aging, McGill University, Montreal, QC, Canada; Global Brain Health Institute, University of California San Francisco (UCSF), San Francisco, CA, USA; Global Brain Health Institute, Trinity College Dublin, Dublin, Ireland; Department of Neurology, Gangnam Severance Hospital, Yonsei University College of Medicine, Seoul, South Korea; Neuroscience and Ageing Research Unit, Institute for Advanced Medical Research and Training; Trinity College Dublin, Dublin, Ireland; Global Brain Health Institute, University of California San Francisco (UCSF), San Francisco, CA, USA; Global Brain Health Institute, Trinity College Dublin, Dublin, Ireland; Latin American Brain Health Institute (BrainLat), Universidad Adolfo Ibanez, Santiago de Chile, Chile

## Abstract

Research on neurodegenerative diseases has predominantly focused on high-income countries in the Global North. This Series paper describes the state of biomarker evidence for neurodegeneration in the Global South, including Latin America, Africa, and countries in south, east, and southeast Asia. Latin America shows growth in fluid biomarker and neuroimaging research, with notable advancements in genetics. Research in Africa focuses on genetics and cognition but there is a paucity of data on fluid and neuroimaging biomarkers. South and east Asia, particularly India and China, has achieved substantial progress in plasma, neuroimaging, and genetic studies. However, all three regions face several challenges in the form of a lack of harmonisation, insufficient funding, and few comparative studies both within the Global South, and between the Global North and Global South. Other barriers include scarce infrastructure, lack of knowledge centralisation, genetic and cultural diversity, sociocultural stigmas, and restricted access to tools such as PET scans. However, the diverse ethnic, genetic, economic, and cultural backgrounds in the Global South present unique opportunities for bidirectional learning, underscoring the need for global collaboration to enhance the understanding of dementia and brain health.

## Editorial note

The Lancet Group takes a neutral position with respect to territorial claims in published maps and institutional affiliations.

## Introduction

The prevalence of cognitive decline and dementia is increasing globally, but with large heterogeneity across regions.^1-6^ Approximately 60% of people living with dementia are estimated to be from low-income and middle-income countries in the Global South. This population is projected to increase to 71% by 2050,^5^ representing an increase of 200%,^6^ with the greatest increase in eastern sub-Saharan Africa, at 357%.^6^ Paradoxically, most dementia research is based in the Global North, with no robust characterisations of the diversity and disparity of the populations in the Global South.^2,7,8^ This imbalance highlights the urgent need for strategies to characterise and care for populations in resource-restricted settings.

Biomarkers are crucial for detecting hallmarks of neurodegeneration longitudinally, assessing disease course, confirming clinical diagnosis, standardising clinical research, and providing biological outcomes for clinical trials.^9,10^ As disease-modifying treatments emerge, biomarkers will become even more valuable.^11^ Although fundamental definitions of biomarkers can differ,^12^ in this Series paper we will use a broad definition: objective measures of typical biological or pathological processes,^13^ including fluids (plasma and cerebrospinal fluid [CSF]), cognition, neuroimaging, and genetics. These measures constitute crucial components for phenotyping and disease characterisation in the Global South and were studied across neurocognitive disorders, including Alzheimer’s disease, mild cognitive impairment (MCI), vascular dementia, frontotemporal lobar degeneration (FTLD), dementia with Lewy bodies, Parkinson’s disease, Huntington’s disease, and amyotrophic lateral sclerosis (ALS).

For this Series paper, the term Global South will be used instead of the World Bank classification of countries. Although neither term is perfect because of the grouping of countries with different socioeconomic and health dynamics under the same umbrella, the World Bank classification is more fine grain, which can result in a misleading representation. This detailed economic breakdown overlooks the profound inequalities existing within regions such as Latin America, which, despite having countries such as Chile, a high-income country, is one of the most economically disparate regions globally. This region has risk factors distinct from other high-income countries in the Global North,^1-6,8^ resulting in less than 1% of its population having access to standard biomarkers of neurodegeneration.^10,14-16^ Current research underscores distinguishable differences in genetic backgrounds, risk factors, environmental influences, and brain phenotypes between populations in the Global North and Global South,^1,3,4,6,8,17-20^ highlighting the limitations of universal models in capturing complex brain phenotypes. The understanding of the scientific community is also incomplete because of a lack of validation in diverse and underserved populations and the absence of appropriate cutoff scores^21,22^ and diversity in clinical trials.^9,14,23^ This scenario is further complicated by the heterogeneity of comorbidities, such as cardiometabolic syndromes, observed in the Global South.^3,24^ All these factors call for customised approaches that go beyond universal models to deliver better results.

The National Institute of Aging (NIA) Health Disparities Research Framework^25^ proposes that environmental, sociocultural, behavioural, and biological factors work together to influence ageing, where race is a sociocultural factor rather than a biological one,^3,26-28^ emphasising biological and cultural interactions.^14,23,29-32^ Similarly, allostasis models have evidenced the effect of the physical and social exposomes across pathophysiological pathways.^20,30,32-34^ This multifactorial framework can be applied to the new definitions proposed by the NIA and the Alzheimer’s Association, contributing to refining the AT(N) binary scheme in Alzheimer’s disease. Systematic inclusion of regions in the Global South in biomarker research offers the best opportunity to examine this framework in a more complex but realistic way.

This Series paper considers representative research conducted on fluid, cognitive, neuroimaging, and genetic biomarkers in Latin America, Africa, and countries in south, east, and southeast Asia (hereafter referred to as South-East Asia), to contextualise biomarker research in the Global South. Rather than a narrow focus, the aim of this selective review is to provide an overview of the state of biomarker research in the Global South, including gaps and potential opportunities. The challenges and unique opportunities for bidirectional learning between the Global South and Global North are then discussed with recommendations for research and policy initiatives to boost biomarker research in the Global South.

## Overview of biomarker findings across the Global South

**Figure 1:**
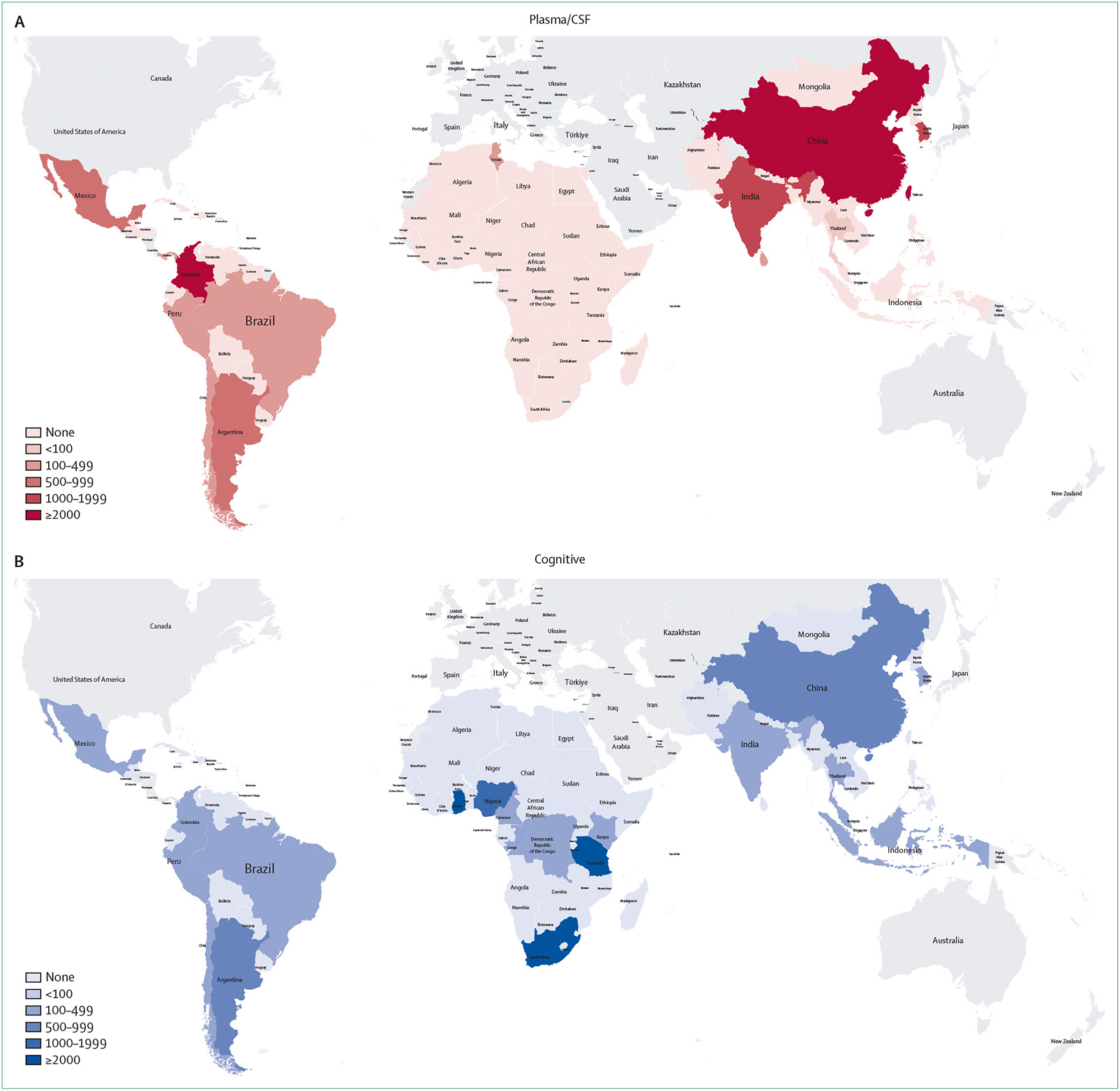

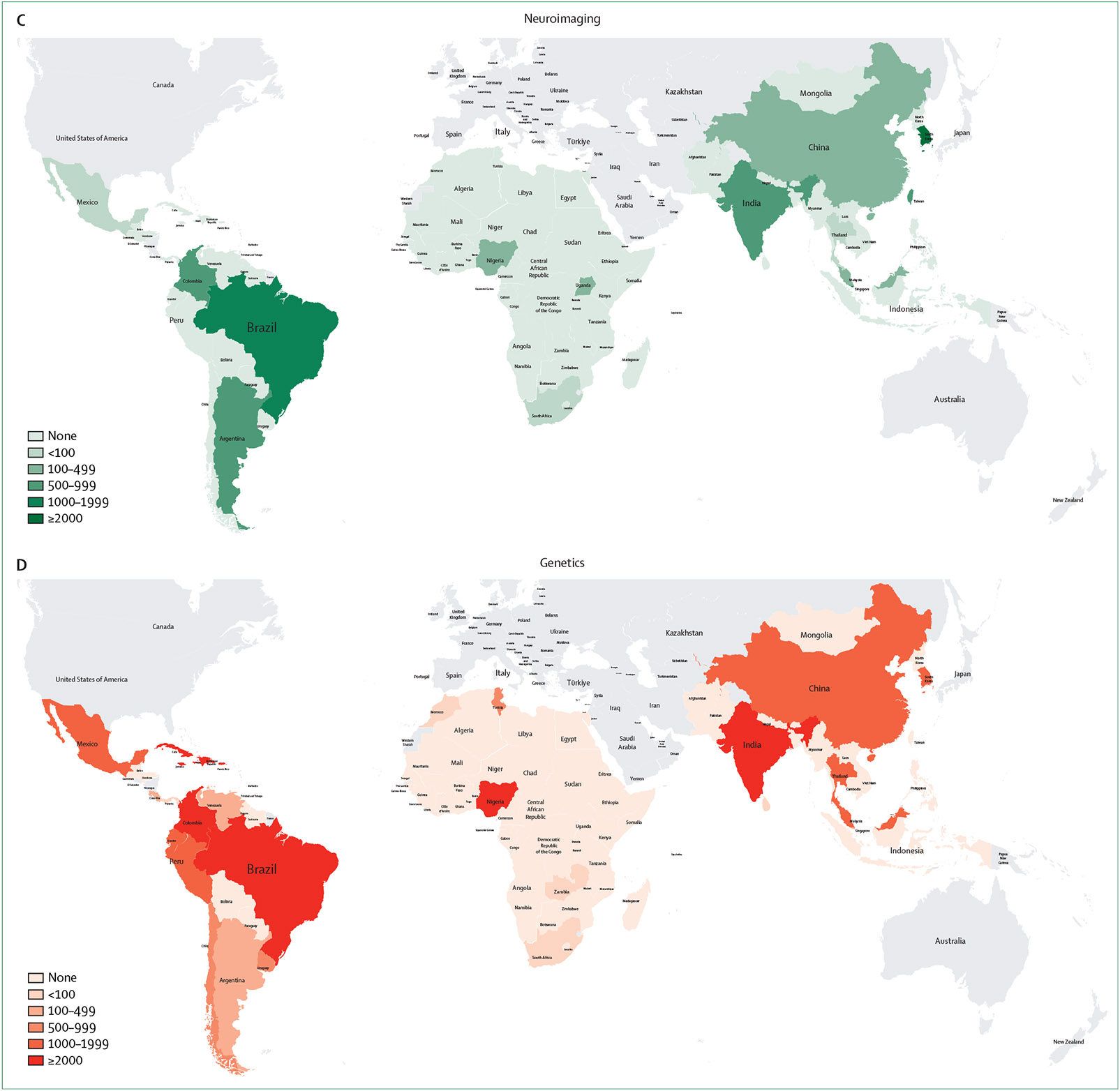
Participant distribution in studies included in the selective review per biomarker type across the Global South Heatmap illustrating the number of participants included in the selected biomarker studies across countries in the Global South. The figure illustrates the included studies across all types of neurodegenerative diseases across the different biomarker types: fluid biomarkers including plasma and CSF **(A)**, cognitive studies **(B)**, neuroimaging studies **(C)**, and genetic studies **(D)**. Due to the large number of controls in some genetic studies, figures for genetic studies exclude number of controls. CSF=cerebrospinal fluid.

The search and identification strategies are summarised in the appendix p 2. Following screening, 455 studies were included and summarised in tables for the review (table 1). Details of the studies are provided in the appendix pp 5-140. Differences in the areas of focus of the studies included were observed across regions and biomarker types (figure 1). Additional summary information on the studies is included in the appendix p 4.

## Plasma and CSF

### Latin America

The study of fluid biomarkers is progressing in Latin America, with particular focus on Alzheimer’s disease and related dementia. Research in Colombia is focused mostly on carriers of the *PSEN1* E280A mutation, related to familial Alzheimer’s disease. Argentina, Brazil, Peru, and Chile are actively involved in CSF biomarker research, although data from several countries in Latin America were notably absent from the reviewed studies. Across studies, classic CSF biomarkers (amyloid β [A β]1–42, total tau, and phosphorylated tau181) consistently differentiated individuals with Alzheimer’s disease from controls.^35-38^ Individuals with Alzheimer’s disease generally had lower levels of Aβ1–42 and higher levels of total tau and phosphorylated tau181 than did the healthy controls^35,39-41^ and individuals presenting with MCI;^35,39,41-43^ in addition, combined biomarker ratios (eg, Aβ1–42 to phosphorylated tau181) showed potential as diagnostic tools. Elevated CSF neurofilament light levels were observed in individuals with Alzheimer’s disease and behavioural variant frontotemporal dementia, compared with those in controls and individuals with MCI.^36^ With respect to blood (plasma or serum) biomarkers for Alzheimer’s disease, CTACK, MIG, and SDF-1α had discriminative power, with AnxA1 and LX4A capable of distin-guishing Alzheimer’s disease from behavioural variant frontotemporal dementia.^37^ To the best of our knowledge, no plasma studies with Aβ, tau, or neurodegeneration biomarkers related to sporadic cases of Alzheimer’s disease or FTLD have been performed in Latin America. The incorporation of novel plasma biomarkers (AnxA1, LX4A, and SNCA mRNA) and cytokines showed the potential for expanding research beyond traditional markers.^38^

### Africa

A study related to Alzheimer’s disease carried out in plasma samples from participants from the Democratic Republic of the Congo^44^ found that plasma Aβ40/42 was significantly associated with lower scores on the Community Screening Interview for Dementia (CSID) and higher scores on the Alzheimer’s Questionnaire in healthy controls, but not in individuals with dementia. However, phosphorylated tau181 displayed no significant associations with either measure in this study. A study in Tunisia that examined CSF biomarkers in 103 participants with Alzheimer’s disease and non-Alzheimer’s disease dementia^45^ found that the average folate levels in the CSF were lower in participants with Alzheimer’s disease than in controls. No correlation was found between Aβ1–42 or total tau and folate or homocysteine in the CSF. A significant inverse correlation between homocysteine and folate in the CSF was observed in the Alzheimer’s disease group, whereas a significant correlation for homocysteine between the plasma and CSF was found in the non-Alzheimer’s disease group. No other relevant studies were identified.

### South-East Asia

India and China were the most active countries in this region for both plasma and CSF research in Alzheimer’s disease and related dementia, with China also having a strong focus on Parkinson’s disease. Taiwan had presence in both plasma and CSF research, whereas Thailand and Singapore primarily focused on CSF biomarker research. Studies from India focused on apolipoprotein E (*APOE*), interleukin-6, brain-derived neurotrophic factor, clusterin, and others, which identified associations between genetic markers (eg, *APOE* ε4 allele) and the risk of Alzheimer’s disease or vascular dementia.^46-49^ Studies in China investigated multiple markers such as Aβ42, total tau, phosphorylated tau, neurofilament light,^50-52^ and glial fibrillary acidic protein and identified that elevated levels of phosphorylated tau and glial fibrillary acidic protein are associated with cortical thickness.^53^ Neurofilament light levels were significantly associated with Alzheimer’s disease and inversely correlated with cognitive function. Research carried out in Taiwan examined plasma biomarkers such as Aβ1–42, Aβ1–40, total tau, and phosphorylated tau, with one study highlighting the potential role of *SORL1* in Alzheimer’s disease and MCI.^54^ Although South-East Asia had a larger set of plasma and CSF studies in this review, multiple countries such as Indonesia, the Philippines, Malaysia, and Sri Lanka presented scarce research.

## Cognition

### Latin America

Most of the research from Latin America originated from countries that have developed multicentric collaboration, such as Argentina, Brazil, Chile, Colombia, Mexico, and Peru. The studies used various cognitive assessments (Mini-Mental State Examination [MMSE], Rowland Universal Dementia Assessment Scale, Montreal Cognitive Assessment [MoCA], Addenbrooke’s Cognitive Examination III, Ineco Frontal Screening, Pfeffer Functional Activity Questionnaire, Neuropsychiatric Inventory, or Mini-Social Cognition & Emotional Assessment) and disease types (Alzheimer’s disease, FTLD, MCI, and Parkinson’s disease), with some studies suggesting that the cognitive assessments allowed for potential disease discrimination.^27^ Although the level of education of participants^27,55^ had some influence, these cognitive tests were sensitive and partly specific to disease discrimination. Despite the fact that computational developments have started to control for the substantial sources of variability between studies,^27^ harmonisation and standardisation of assessments are urgently needed.

### Africa

Most research was concentrated in east Africa (Tanzania and Kenya), west Africa (Nigeria), and South Africa, with few reports from north and central Africa. Many African countries were not represented in this research. Studies used various cognitive assessments (Identification of Elderly Africans Instrumental Activities of Daily Living, Alzheimer’s Disease Assessment Scale-Cognitive Subscale, Kiswahili version of the MoCA, India Human Development Survey, or CSID), which include instruments adapted for Africa.^56^ The CSID, developed by the Ibadan–Indianapolis Dementia Project, was the first key cognitive assessment tool developed for cognitive assessment in Africa.^56^ The CSID was subsequently translated into multiple local African languages and used for dementia screening in rural populations in Kenya and Tanzania in east Africa. The Identification of Elderly Africans Instrumental Activities of Daily Living Cognitive Screen is a derivative of the CSID, which, at a cutoff of 7 or less, presented sensitivity in the range from 59% for participants in Tanzania^57^ to 100% for those in Nigeria,^58^ with the level of education of participants affecting scores.^59^ In Ibadan, Nigeria, an adequate association was found between the Clinician Home-based Interview to assess Function and Blessed Dementia Scale.^60^

Adapted cognitive tests such as the Kiswahili version of the MoCA showed acceptable reliability, concurrent validity, sensitivity, and specificity in diagnosing MCI and dementia.^61^ The strength of these tests flowed from development of culturally adapted cognitive assessments and use of multimodal approaches. However, low representation of African countries in cognitive studies of neurodegeneration underscored the need for further validation in larger and more diverse populations.

### South-East Asia

Most studies came from South Korea, Singapore, India, Thailand, Indonesia, and the Philippines using MMSE, MoCA, Visual Cognitive Assessment Test, Boston Naming Test, and Clinical Dementia Rating tools. MMSE, MoCA, and Visual Cognitive Assessment Test were effective in detecting cognitive impairment in Alzheimer’s disease, with MMSE and MoCA useful in detecting cognitive changes in MCI. The Hindi version of MMSE and the Informant Questionnaire on Cognitive Decline in the Elderly were adapted for Hindi-speaking populations, and the Addenbrooke’s Cognitive Examination III was validated in seven Indian languages.^62^ Cognitive functions in rural Indonesian populations were influenced by the level of education.^63^ One study combined cognitive assessments with biomarkers and identified significant associations between the Thai version of the Boston Naming Test and the *APOE* ε4 allele,^64^ with another study addressing the cultural and educational factors influencing the detection of cognitive impairment using the Hindi version of the MMSE in a comparison between illiterate and literate participants.^65^ Virtual reality testing also showed potential promise for detecting MCI.^66^

## Neuroimaging

### Latin America

A large number of studies included MRI, diffusion tensor imaging, and functional MRI sequences focusing on different aspects, including diagnosis classification, disease stages, and subtype characterisation,^67-73^ whereas a few studies also conducted PET imaging for assessing brain glucose metabolism, and amyloid and tau load.^74-79^ The most frequent studies provided associations with clinical and cognitive measures but did not evaluate the association with fluid biomarkers. PET imaging showcased associations between phosphorylated tau217, amplified future amyloid and tau pathologies, and impaired future memory performance.^80^ Notably, the carriers of the Colombian *PSEN1* E280A mutation presented heightened amyloid and tau PET loads.^80-82^ Distinct correlations were found between sleep patterns, amyloid loads, and cognitive impairments.^83^ Specific associations between subicular volumes, Aβ deposition, and hippocampal subfield volume reductions emphasised the higher potential of early-phase ^11^C-Pittsburgh compound B ([^11^C]PiB)-PET in offering insights into neurodegeneration than [^18^F]fluorodeoxyglucose ([^18^F]FDG)-PET.^74^ Brazilian studies successfully implemented the amyloid and [^18^F]FDG-PET in the context of the 2018 NIA-Alzheimer’s Association research framework but highlighted clinical biomarker mismatches with sociodemographic effects.

### Africa

Overall, studies interrogating neuroimaging markers of neurodegeneration were sparse in Africa, which might be due to the paucity and high cost of relevant neuroimaging modalities. However, a study that evaluated neuroimaging correlates of cognition among survivors of stroke in Nigeria used a range of cognitive measures to map cognitive functionalities against brain measures. The potential vascular basis of neurodegeneration rooted in cerebral hypoperfusion was a relevant insight from that study.^84^ Additionally, a significant association was reported between carotid artery plaque and abnormal cognitive functions.^85^ A subsequent study in South Africa conducted analyses of glucose metabolism patterns using [^18^F]FDG-PET scans to observe association with cognitive imbalances in Parkinson’s disease and parkinsonian-plus disorders, including multiple system atrophy and dementia with Lewy bodies.^86^ The analyses presented encouraging results regarding sensitivity, specificity, and agreement percentages in diagnoses. A few of the missing focus areas include a substantial gap in the fundamental assessments of neuroimaging for the characterisation of neurodegenerative disease subtypes, stages, and severity. The region had few multicentric studies and comparisons with other regions, and systematically reported publications, indicating a pressing need for a more structured approach to researching neurodegenerative diseases in Africa.

### South-East Asia

A comprehensive range of results, primarily from MRI, provided clear delineations about biomarkers between different conditions (Huntington’s disease, MCI, and Alzheimer’s disease) across studies, with correlations with cognition, protein accumulations, and atrophy,^87-92^ where few assessed functional brain activity.^93^ Ethnic variations in neuroimaging markers were observed alongside potential markers offering high sensitivity and specificity in detecting cerebrovascular disease and neurodegeneration.^90^ Multimodal approaches have explored a spectrum of recognised biomarkers, representing a key strength of these studies—the ability to offer a rich and detailed view of the disease processes. However, the vast array of metrics and variables introduced complexity that could overshadow clear conclusions.

## Genetics

### Latin America

Over 140 genetic studies across Latin America have been conducted, with over 90 focused on Alzheimer’s disease. The rich admixture of Indigenous, African, and European ancestries^14-16,70,75^ in the region influenced genetic mutations and risk across diseases. For Alzheimer’s disease, the most substantial kindred of *PSEN1* was in Colombia, with multiple other genetic presentations of familial Alzheimer’s disease.^94-102^ Different alleles and mutations in genes such as *PSEN1, PSEN2, PICALM,* and *BIN1,* among others, were associated with Alzheimer’s disease to varying degrees.^103,104^ Strong associations between the *APOE* ε4 allele and an increased risk of Alzheimer’s disease were accompanied by a protective effect of *APOE* ε2. Indigenous ancestry and Alzheimer’s disease risk associations suggested some protective benefits,^105^ whereas African ancestry was associated with increased risk.^103^ Single-nucleotide polymorphisms and haplotypes in various genes were associated with Alzheimer’s disease risk and protection, with differences noted between Europeans and a Brazilian cohort.^104^ Although the *APOE* ε4 is a key risk factor for Alzheimer’s disease in various populations, the role of other genetic factors remains unclear. The detection of G4C2 expansions in the *C9orf72* gene was observed in both familial and sporadic FTLD and ALS cases, with a higher prevalence in ALS-FTLD.^106-108^ Novel mutations and prevalent genetic variations included novel *GRN* mutations in FTLD cases and significant associations of *GRN* and *MAPT* mutations with familial FTLD.^109^ Genetic variations associated with Parkinson’s disease across ethnic and geographical groups included the recessive model of rs35479735, the variable prevalence of *LRRK2* p.G2019S (European ancestry), and risk stratifications of *NR4A2, GBA,* and *MTHFR* variations. Other genetic kindreds of additional diseases, such as Cerebral Autosomal Dominant Arteriopathy with Subcortical Infarcts and Leukoencephalopathy and Huntington’s disease, were also observed in the region. Leveraging the rich genetic admixture offers unique insights into risk and protective factors.

### Africa

Studies identified associations between genetic markers and biomarkers with disease risk and cognitive impairments (Alzheimer’s disease, Parkinson’s disease, ALS, and Huntington’s disease). In Zambia, novel disease-causing variants and deletions have been observed for early-onset Parkinson’s disease.^110^ A novel genetic risk factor associated with Parkinson’s disease in African and African admixed populations recruited from the International Parkinson’s Disease Genomics Consortium Africa, which has not been seen in the European population, could be a mechanistic basis for Parkinson’s disease in the African population.^111^ *APOE* genotypes were associated with cognitive impairment in individuals with Parkinson’s diseas.^112^ The mutational spectrum in ALS included *TARDBP, C9orf72,* and *SOD1* in Tunisia.^113^ Carriers of mutations in *PSN1* have also been identified. African Americans from Indiana, USA, and the Yoruba population in Nigeria presented links between *APOE* ε4 homozygosity and increased risk of Alzheimer’s disease and late-onset Alzheimer’s disease; however, this remains a controversial finding in African populations, as the reason for this association remains unclear.^114^ Protective loci against *APOE* ε4-associated risk for Alzheimer’s disease in African ancestry populations in Nigeria have also been reported.^115^ Some participants presented synergistic effects of multiple alleles on dementia risk.^116^ High prevalence of Huntington’s disease was associated with CAG repeat length in the *IT15* gene in a Moroccan population.^117^ Various African and admixed populations allowed for a rich analysis from diverse geographical and demographic sources, with novel variants identified, in addition to risk and protective factors; however, the small sample sizes in the studies identified limited the robustness of the findings. The cognitive and brain measures used varied substantially across studies, thus making direct comparison of the results challenging.

### South-East Asia

A diverse set of insights from research from this region identified well established associations, such as the one between the *APOE* ε4 allele and Alzheimer’s disease, and novel insights including previously unidentified genetic variations from India that are involved in amyloid signalling.^118^ Variations and mutations in *APOE*,^119-122^ *PGRN, MAPT*,^123,124^ and others were associated with disease risk, with strong association between *APOE* ε4 allele and Alzheimer’s disease and vascular dementia. Conversely, ε3 and ε2 alleles appeared to be protective and ε4 alleles were rare in some cohorts. A small proportion of FTLD cases had *GRN* mutations.^123,125^ These include potentially pathogenic gene mutations such as *GRN, SQSTM1, LRRK2, NOTCH3,* and *HTRA1.* New *MAPT* variants increased tau phosphorylation.^126^ A large study from South Korea identified novel variants of Alzheimer’s disease based on whole-genome sequencing of *APOE* ε4 carriers.^127^ Serum brain-derived neurotrophic factor levels were higher in participants with Alzheimer’s disease and amnestic MCI than in controls, although no substantial effects of the Val66Met polymorphism were found on these levels.^48^ Multiple genetic analysis techniques included whole-genome sequencing, and some studies integrated data types (such as customised Alzheimer’s disease chip data, which focus on specific genetic markers associated with Alzheimer’s disease, and whole-genome sequencing data).^127^ In Parkinson’s disease, the largest study and a multiethnic cohort on *GBA* variation from South-East Asia, encompassing Chinese, Malay, and Indian ethnicities, detected three novel variants but no common European risk variants, highlighting the need to include these populations in clinical trials targeting *GBA* pathways.^128^

## Summary of biomarkers in the Global South

The strengths of the reviewed studies are in the diversity of population sampling, integration of varied data types, and identification of novel genetic variants. Although emerging research provides promising agendas, most of these initiatives were not integrated with one another, did not have multicentric comparisons, and were substantially lower in number, as compared with those carried out in the Global North.^1,3,6,8^ In Latin America,^70,75,129^ fluid biomarkers are emerging with a notable focus across the Alzheimer’s disease continuum.^129^ Cognitive assessments revealed robust disease discrimination, with a need for more harmonised methods. Neuroimaging studies, especially those involving MRI and PET, provided insights into diagnosis, disease stages, and subtype characterisations. Genetic studies offered insights into diverse mutations and risk associations, with the *PSEN1* mutation being crucial in Alzheimer’s disease research. In Africa, although the research landscape^111^ is sparse in terms of plasma and neuroimaging studies, genetic studies presented relevant associations and novel mutations. Cognitive research, largely centred in east and west Africa, emphasised the need for adapted cognitive tests. In South-East Asia, India, China, and Taiwan^130,131^ conduct most research on plasma and CSF biomarkers in Alzheimer’s disease and Parkinson’s disease, also emphasising the effectiveness of various cognitive tools in detecting impairment. Neuroimaging studies provided a comprehensive understanding of cognitive decline and dementia with genetic research emerging, covering both well established and novel associations. Although not a focus of this study, notably, brain banks have been established in Brazil, Argentina, India, Mexico, China, and Nigeria^132,133^ (although they are still less common than in the Global North), offering the potential for unique discoveries across neurodegenerative diseases.

## Challenges

The Global South is characterised by large heterogeneity within and between regions,^1,3,4,8,17-19^ with areas of expertise, disparities, and gaps, suggesting the need for different approaches across different regions to support the development of biomarkers. In Latin America and South-East Asia, hubs of expertise in plasma and CSF research are emerging, but plasma research is notably absent in Africa. Evidence of risk associated with the *APOE* ε4 allele among Africans is conflicting,^134^ with less than 2% of genome-wide association studies comprising African data.^21^ As less than 3% of studies originate from African, Indigenous, or Latin American participants,^135^ little is known about the genetic diversity,^14,17,19^ polygenic risk scores, socioeconomic disparities,^8^ or the biology of ageing in these under-represented populations.

At the structural level, funding scarcity, inadequate capacity building, and scarcity of infrastructure create challenges across the regions.^10,136-140^ PET has been identified as highly limited, prohibitively expensive, and available only in advanced research centres (<1% of the population have access to PET in Latin America).^10^ Robust computational frameworks to assess multimodal neuroimaging,^141^ especially new techniques involving whole-brain modeling,^20^ generative biophysical models,^142^ and deep learning, are absent.^68^ Lumbar puncture for CSF collection has been highlighted as a challenge in Brazil, as the technique is available only in large cities and not covered by the public health system.^136^ Sociocultural stigma is tied to traditional spiritual beliefs, linking behaviours related to dementia to witchcraft in some parts of sub-Saharan Africa,^143-145^ but without sufficient investigation in understanding how dementia is conceptualised within Indigenous knowledge systems. The introduction of low-cost biomarkers alone might not be sufficient to facilitate dementia diagnosis in the Global South. Although cost is a key factor, logistical, technical, and cultural factors also pose barriers.

## A bidirectional approach

A bidirectional approach with the Global North is essential for fostering biomarker research in the Global South, in terms of its goals, methods, benefits, and effects. Limitations of cognitive assessments even within the context of the Global North are acknowledged, with factors such as language of administration, sex or gender, urbanicity, and race or ethnicity possibly affecting cognitive test scores.^146^ Therefore, although neuropsychological testing is a challenge in the Global South because of the scarcity of standard tools and culturally adapted cutoffs,^147^ standardisation of instruments might not be sufficient for valid measurement, but might additionally need to be combined with harmonised procedures^148^ to assess the degree of socioeconomic and cultural heterogeneity. In addition, the limitations and biases of applying methods and findings of the Global North in the context of the Global South should be understood. Risk factor models and brain phenotype associations with stereotypical populations in the Global North do not necessarily apply to the Global South.^1-3,16,17,149^ Additionally, cardiometabolic factors (such as cardiovascular disease, stroke, and diabetes) that influence some biomarkers affect countries in the Global South^3,24^ to a substantially greater extent than those in the Global North. Although mortality owing to cardiovascular disease has decreased in the Global North, it has increased in the Global South due to environmental, social, political, and commercial determinants of health.^24^ Complex interactions between pathophysiological pathways (inflammatory, stress-related, microbiome, and immune) and social and physical exposomes^8,33,34,150^ represent a challenge for biomarker research. These differences highlight the need to consider adaptations of the Global North strategies for the Global South carefully and the need for capacity building across its regions for localised research, to identify region-specific factors and solutions.

## Unique opportunities

The validation of plasma biomarkers of neurodegeneration, including Aβ42/40, phosphorylated tau epitopes, glial fibrillary acidic protein, neurofilament light, and others, in diverse settings will allow for a robust assessment of cognitive health of under-represented populations. Additionally, the bidirectional benefit and effect of advancing biomarker research in the Global South will create opportunities globally. The inclusion of people from regions in the Global South in genomic research on dementia, including genome-wide association study and whole-exome approaches or whole-genome approaches, could provide novel insights into the biology of Alzheimer’s disease and other phenotypes,^21^ thereby increasing the external validity of results across ethnicities, racial origins, and multicultural backgrounds.^151^ Such a step will also improve the understanding of polygenic risk factors and interactions with the exposome in the characterisation of biomarkers.

Dementia research at a global level calls for more diversity, where universal and generalisable findings should not be the desired findings across diverse contexts. Insights across brain health, cognitive neuroscience, genetics, and dementia research highlight the inadequacy of universal models for healthy ageing and dementia. Incorporating the multimodal diversity from the Global South can transform the understanding of brain health and dementia globally towards more customised, personalised, and efficient characterisation of biomarkers.

## Future prospects

**Figure 2:**
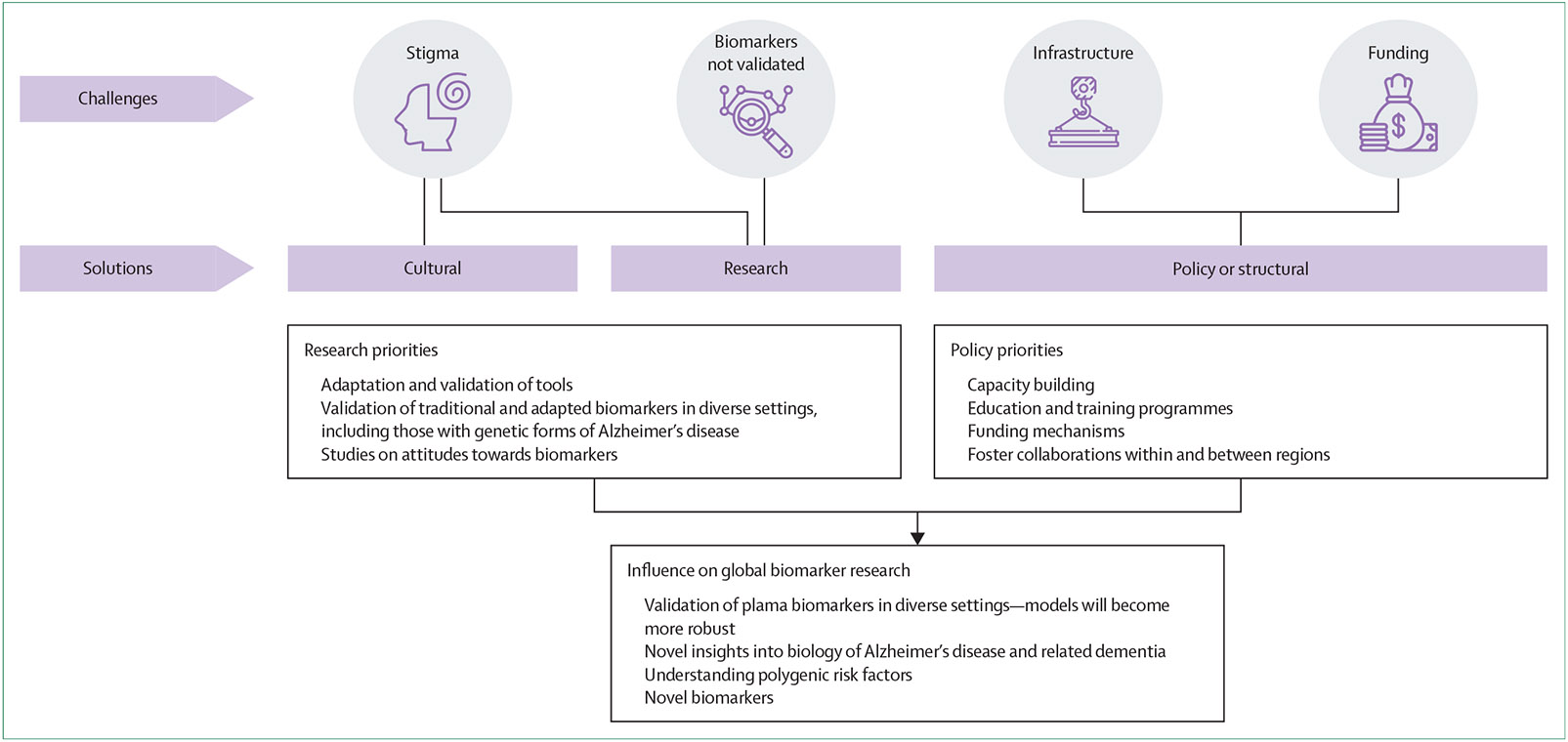
Multisectoral responses to challenges in biomarker research and potential effects in the Global South Challenges faced by biomarker research in the Global South and potential solutions are illustrated. Identified challenges such as stigma, validation of biomarkers, infrastructure, and funding require multisectoral solutions across cultural, research, and policy sectors. Suggested research and policy priorities to address these challenges are described. The effects of and potential for biomarker research at a global scale should multiple sectors work together to actualise these priorities are highlighted.

Coordinated efforts across research, policy, and cultural realms (figure 2), including short-term, medium-term, and long-term strategies (table 2), are needed. Although commonalities exist, strategies should be tailored and adapted at the local and regional levels, considering the specific needs of different cultures. Some strategies focus on the development of infrastructure and environment, whereas others focus on ways in which developments in biomarkers could be speedily implemented. For example, the measurement of neurofilament light protein, glial fibrillary acidic protein, phosphorylated tau, and Aβ in dried blood spots^152,153^ has the advantage of being unaffected by pre-analytical factors, such as time to centrifugation, temperature of freezing, and tube type.

From a cultural perspective, collaborations with local communities, health-care providers, and policy makers are needed to create a more comprehensive, context-specific approach to mitigate cultural and geographical barriers.^138-140,148^ Including diverse populations in research on biomarkers should be handled ethically and responsibly, to avoid exploitation. Ethical guidelines and robust community engagement should be integral to this process, with the long-term goal of multisectoral coordination. Global North–Global South partnerships need to redress regional imbalances,^154^ guided by the principles in documents such as the Africa Charter on Transformative Research Collaborations.^155^ A pluralistic approach to health care and dementia education and awareness are required to overcome stigma and challenge cultural beliefs of dementia as a prejudiced condition^156^ and an adscription of witchcraft.^144^ As a long-term strategy, mobilising resources across regions and disciplines using a brain health diplomacy model^156,157^ can inform policy around biomarkers and health care.^156^

One such example of this type of model is the Global Brain Health Institute (GBHI),^158^ an interdisciplinary training programme based at University of California San Francisco and Trinity College Dublin, which is committed to advancing equity in brain health by training brain health leaders. Similarly, the Alzheimer’s Association supports collaborative research between regions. The largest Latin American consortium on dementia, the Latin American and Caribbean Consortium on Dementia, which includes the Multi-Partner Consortium to Expand Dementia Research in Latin America (ReDLat)^15,16,27,68^ and the first regional centre (BrainLat),^159^ was originally supported by GBHI and Alzheimer’s Association. ReDLat studies genetic factors and socioeconomic disparities, such as inequality measures, influencing Alzheimer’s disease and FTLD, involving more than 4000 participants from 13 centres across six countries (Chile, Argentina, Brazil, Peru, Colombia, and Mexico) in Latin America as well as the USA. The study investigates genetic–environmental interactions^29,30,160^ using harmonised protocols in clinical, cognitive, genomic, and socioeconomic areas across varied Latin American populations. Initially funded in the USA, Argentina, Brazil, Colombia, and Peru, ReDLat later included Mexico and Chile, with additional support from the Alzheimer’s Association, Rainwater Charitable Foundation, and GBHI. Partnerships with National Institutes of Health-Center for Alzheimer’sand Related Dementias, Alector, Takeda Pharmaceuticals, and the Bluefield Foundation have facilitated research on large families with genetic Alzheimer’s disease and FTLD variants. In addition, new National Institutes of Health grants support ecological assessments^161^ involving epigenomics, speech biomarkers, and circadian imbalances.

In Africa, the African Dementia Consortium brings together over 100 researchers in a multidisciplinary framework to generate clinical and socioeconomic datasets to improve the characterisation of dementia in Africa,^162^ and the Brain Research Africa Initiative is focused on the translation of brain health evidence for policy and development.^163^ Each of these approaches illustrates the importance of customised local and regional models that can facilitate translational genomics and improve the understanding of global dementia phenotypes, with potential to identify causal genetic variants.^162^ The transdisciplinary nature of these stakeholders has the potential for effects across cultural, policy, and research realms to advance research and use of biomarkers, both in the Global South and Global North.

A pressing need for harmonisation across methodologies and regions exists. Establishing a common research agenda on the basis of Global South–Global South and Global South–Global North comparisons could gain richer insights and identify discrepancies. Systematic research protocols would ensure more accurate and reproducible findings, and multicentric, multiregional characterisations would enhance the depth and breadth of studies. Unique regional characteristics, such as the inclusion of Indigenous populations in Latin America, diverse genetic variations highlighted in South-East Asia, and the rich genetic diversity observed in Africa, underscore the need for cross-regional collaborations. Leveraging these unique attributes in a harmonised research framework could accelerate and enhance our understanding of neurodegenerative diseases on a global scale.

Latest advances in research can enhance feasibility in the measurement of neurodegeneration in remote settings, for instance with the use of dried blood spots.^152^ Similarly, cognitive digital biomarkers in the Global South should be systematically examined to establish their specificity and generalisability across different populations and their potential to be more accessible^21^ to people in rural areas and outside capital cities.

## Conclusions

The biomarker agenda of the Global South needs to overcome both global and region-specific challenges, in addition to recognising its existing strengths and fostering bidirectional collaboration with the Global North. The challenges in infrastructural, financial, technical, cultural, and logistical domains make integrative solutions indispensable. The influence of sociocultural factors on health underscores the importance of localised and context-specific research to suit the uniqueness of different populations, and to better understand the landscape of neurodegeneration in the Global South. However, the silver lining in this landscape is the unique opportunities that emerge from these challenges, with mutual benefits of reducing biases and advancing scientific knowledge at the global level. If approached with an inclusive and ethical mindset, then biomarker research in the Global South can pave the way for groundbreaking discoveries that are globally relevant and locally sensitive. Integration with the multifaceted diversity inherent in these regions will redefine our approaches towards research on brain health, dementia, and biomarkers. For this, the global community should come together to foster collaborations and champion a shared, comprehensive roadmap that is rooted in equity, inclusivity, and innovation.

## Supplementary Material

1

## Figures and Tables

**Table 1:** Overview of the biomarker studies reviewed in this work

	Number of studies			
	Plasma or CSF	Cognitive	Neuroimaging	Genetics
**Latin America**				
Argentina	7	3	11	9
Brazil	6	2	27	60
Mexico	5	2	1	17
Chile	2	2	⋅⋅	5
Bolivia	⋅⋅	⋅⋅	⋅⋅	⋅⋅
Peru	1	2	⋅⋅	1
Colombia	1	1	16	23
Panama	1	⋅⋅	⋅⋅	1
Venezuela	⋅⋅	⋅⋅	⋅⋅	3
Multiple countries in the Caribbean	1	⋅⋅	1	25
Ecuador	⋅⋅	⋅⋅	⋅⋅	4
Costa Rica	⋅⋅	⋅⋅	⋅⋅	1
**Total Latin America**	24	10*	58†	149
**Africa**				
Nigeria	⋅⋅	4	2	4
Tunisia	1	⋅⋅	⋅⋅	4
Morocco	⋅⋅	⋅⋅	⋅⋅	2
South Africa	⋅⋅	2	1	1
Uganda	⋅⋅	⋅⋅	1	⋅⋅
Tanzania	⋅⋅	9	⋅⋅	⋅⋅
Kenya	⋅⋅	1	⋅⋅	⋅⋅
Republic of the Congo	⋅⋅	1	⋅⋅	⋅⋅
Ghana	⋅⋅	1	⋅⋅	⋅⋅
Cameroon	⋅⋅	1	⋅⋅	⋅⋅
Zambia	⋅⋅	⋅⋅	⋅⋅	1
Democratic Republic of the Congo	1	⋅⋅	⋅⋅	⋅⋅
**Total Africa**	2	18*	4	12
**South-East Asia**				
India	13	3	14	17
China	28	1	2	7
Taiwan	10	⋅⋅	4	⋅⋅
Thailand	2	1	1	4
South Korea	8	3	23	7
Singapore	10	3	7	3
Indonesia	⋅⋅	1	⋅⋅	⋅⋅
Philippines	⋅⋅	⋅⋅	⋅⋅	⋅⋅
Malaysia	⋅⋅	3	1	4
Sri Lanka	1	⋅⋅	⋅⋅	1
Multiple countries	⋅⋅	⋅⋅	⋅⋅	5
**Total South-East Asia**	72	15	52	48

CSF=cerebrospinal fluid. *Some studies included multiple countries. †In two studies from the Latin American Consortium, individual countries were not described.

**Table 2:** Strategies in different domains for biomarker development across the Global South

	Short-term strategies	Medium-term strategies	Long-term strategies
Cultural	Culturally sensitive outreach Develop regional ethical guidelines for biomarker research Awareness and education about dementia, including faith and traditional healers and health-care professionals	Raise awareness among policy makers, health-care professionals, and the public about the importance of neurodegenerative disease research Governmental campaigns to address stigma Local organisation plans	Multisectoral coordination Brain health diplomacy actions Work regulations
Policy or structural	Expand education and training through targeted biomarker training programmes for researchers Identify strengths within countries (eg, genetic biomarkers in Colombia) to develop hubs of expertise	Develop partnerships between countries on the basis of regional experience (hubs) for data exchange and collaborative research Foster strong regional and international collaborations	Develop multiregional initiatives with global stakeholders
Research	Preliminary studies in diverse populations and comparison across regions Validate blood biomarkers and explore lower cost techniques such as dried blood spots for validated markers (eg, neurofilament light) Adaptation and validation of international tools Explore digital biomarkers Harmonise protocols across regions	Training and combined centralised and distributed analyses Develop local sensitive tools compatible with international tools Develop longitudinal studies and large datasets Incorporation of Global South data into global studies	Develop customised models of biomarker generalisation Translation of biomarkers into clinic Use region-sensitive brain-phenotype predictive models

## Key messages

- The prevalence of dementia is projected to increase substantially in low-income and middle-income countries in the Global South in the coming years, with an estimated 200% rise by the year 2050, highlighting the urgent need for targeted research and care strategies.
- Emerging research on fluid biomarkers in Latin America shows promise in differentiating Alzheimer’s disease cases from controls. Yet, similar studies in Africa are absent, indicating crucial gaps in biomarker research across the Global South.
- Cognitive assessments in the Global South indicate robust disease discrimination; however, harmonised methods and culturally adapted tools are needed to improve the accuracy and relevance of patient care across diverse populations.
- The Global South faces major challenges in advancing biomarker research in the form of funding scarcity, infrastructure limitations, and sociocultural barriers, necessitating tailored, context-specific strategies and robust community engagement.
- Bidirectional collaboration between the Global North and Global South is essential for uncovering new insights into neurodegenerative diseases, improving external validity, and fostering global advancements in biomarker research.
- Advancing biomarker research in the Global South offers unique opportunities to validate plasma biomarkers in diverse settings, potentially leading to groundbreaking discoveries in the biology of Alzheimer’s disease and other neurodegenerative conditions.
- Coordinated efforts across research, policy, and cultural realms are needed to customise a comprehensive roadmap rooted in equity, inclusivity, and innovation. Concerted efforts should focus on developing infrastructure, implementing culturally sensitive research methods, and ensuring sustainable funding to advance the global understanding and management of neurodegenerative diseases.
