# Supplementary material for: Biomarkers of neurodegeneration across the Global South
Source: Lancet Healthy Longev. Author manuscript; Available in PMC 2024 Nov 6. (PMC11540104; doi:10.1016/S2666-7568(24)00132-6)
Supplement: 1 [file NIHMS2031032-supplement-1.pdf]

THE LANCET
Healthy Longevity

Supplementary appendix

This appendix formed part of the original submission and has been peer reviewed. We post it as supplied by the authors.

Supplement to: McGlinchey E, Duran-Aniotz C, Akinyemi R, et al. Biomarkers of neurodegeneration across the Global South. *Lancet Healthy Longev* 2024. https://doi.org/10.1016/S2666-7568(24)00132-6

Appendix
Biomarkers of neurodegeneration across the Global South

Index
1. Search and Identification Strategy
2. Summary
3. Supplementary Figure 1
4. Supplementary Tables 1-12

1. Search and Identification Strategy

Searches were conducted in PubMed, Google Scholar, Scopus, Scielo across four biomarker types of interest: Fluid, Cognitive, Neuroimaging and Genetics. Variants and combinations for each biomarker type were included using the following criteria: Fluid (Aβ 40, Aβ42, p(all forms) total Tau, Neurofilament light protein (NfL), progranulin, TDP-43, alpha-synuclein, SOD, growth factors proteins (BDNF, VEGF), cognitive ((MMSE, ACE, MOCA) + Social Cognition + functionality scales), neuroimaging (PET, MRI/fMRI/Vascular/multimodal integration + EEG/MEG) and inflammatory proteins) and genetics (Criteria causative/candidate (i.e., PSN1, TARDBP) and + risk variants (i.e., tau Haplotypes H1 and H2 and APOE variants ε2, ε3, ε4) + Parkinson genetic mutations. Neurodegeneration (including AD, MCI, VAD, FTLD, PD, DLB, HD and ALS), was included as the outcome of interest in all searches. Searches were conducted by region, by co-authors with expert knowledge of regional biomarker research; Latin America (EZ, CD), Africa (RA, BA) and South-East Asia (FA, HC), where region of interest was added as an additional search term. Publication format was limited to peer review original research articles. The regions selected for this review (Latin America, Africa, and South-East Asia) were chosen due to their underrepresentation in neurodegenerative research and their unique demographic, genetic, and socio-economic profiles which offer distinct opportunities and challenges for biomarker research. This review aims to provide a representation of studies across the Global South, focusing primarily on those areas with the most research activity, but acknowledges that not every country classified as part of the Global South is included in the review. Regions such as the Middle East or Central Asia, which could also be considered part of the Global South, were not included due to limited available literature within the scope of our study.

To provide a comprehensive overview, strict criteria were not applied to the selection of articles due to significant variability in the volume of literature across regions and biomarker types. In cases where a large number of articles were retrieved, such as cognition in Latin America, articles were screened for relevance. Preference was given to studies with the highest impact factors that offered new insights and represented diverse populations. The studies ultimately included in this review offer a selected yet representative overview of literature on biomarkers in the Global South. To ensure a comprehensive and inclusive approach, the collaboration among all authors was structured. The authors performed the revision based on their specific geographical expertise. They collectively formulated criteria to guide the selection and assessment of relevant literature. The team then divided the responsibilities to cover various aspects of the review, including the structure and key areas of focus. Throughout the process, regular meetings were held to discuss findings, and evaluate the relevance of studies. Special attention was given to incorporating studies that represented the diversity of the populations in the Global South, including those that provided new insights into regional variations in biomarkers and those employing innovative methodologies in particular regions, such as culturally adapted cognitive assessments in Africa, the application of amyloid and tau PET imaging in Latin America, and the integration of genetic data with clinical measures in South-East Asia.This collaborative effort ensured that the review was not only thorough but also balanced, reflecting a wide range of perspectives and contributions to the field. While this review does not include a formal meta-analysis, it does provide a qualitative assessment of the selected studies. Many studies from these regions are observational and cross-sectional, with varying sample sizes and methodologies. The heterogeneity in study design and quality reflects the broader variability in research infrastructure and funding across the Global South. However,

studies were selected based on their methodological rigor, relevance, and contribution to the field, with a preference for those published in high-impact journals.

2. Summary

This article explores biomarker research for neurodegeneration across various regions of the Global South, including Latin America, Africa, and South-East Asia, highlighting the stark disparities in neurodegenerative disease research, which has historically centered on high-income countries in the Global North. Latin America is advancing in fluid biomarker and neuroimaging studies, particularly in genetics, while Africa focuses on genetics and cognition but lacks in other biomarker research areas. South-East Asia, notably India and China, shows significant progress in plasma, neuroimaging, and genetic studies.

The review points out several challenges faced by these regions, including lack of funding, inadequate infrastructure, and the need for better harmonization of studies. There is an emphasis on the vast genetic, cultural, and socio-economic diversity in these regions as a unique opportunity for bi-directional learning that could enhance global understanding of dementia and brain health.

A global collaboration is emphasized as necessary to improve research frameworks, integrate diverse genetic and cultural contexts, and build capacities to address and understand neurodegenerative diseases more effectively worldwide. The key message is the critical need for a more inclusive research approach that considers the unique circumstances of the Global South to enrich the overall understanding and treatment of neurodegenerative diseases globally.

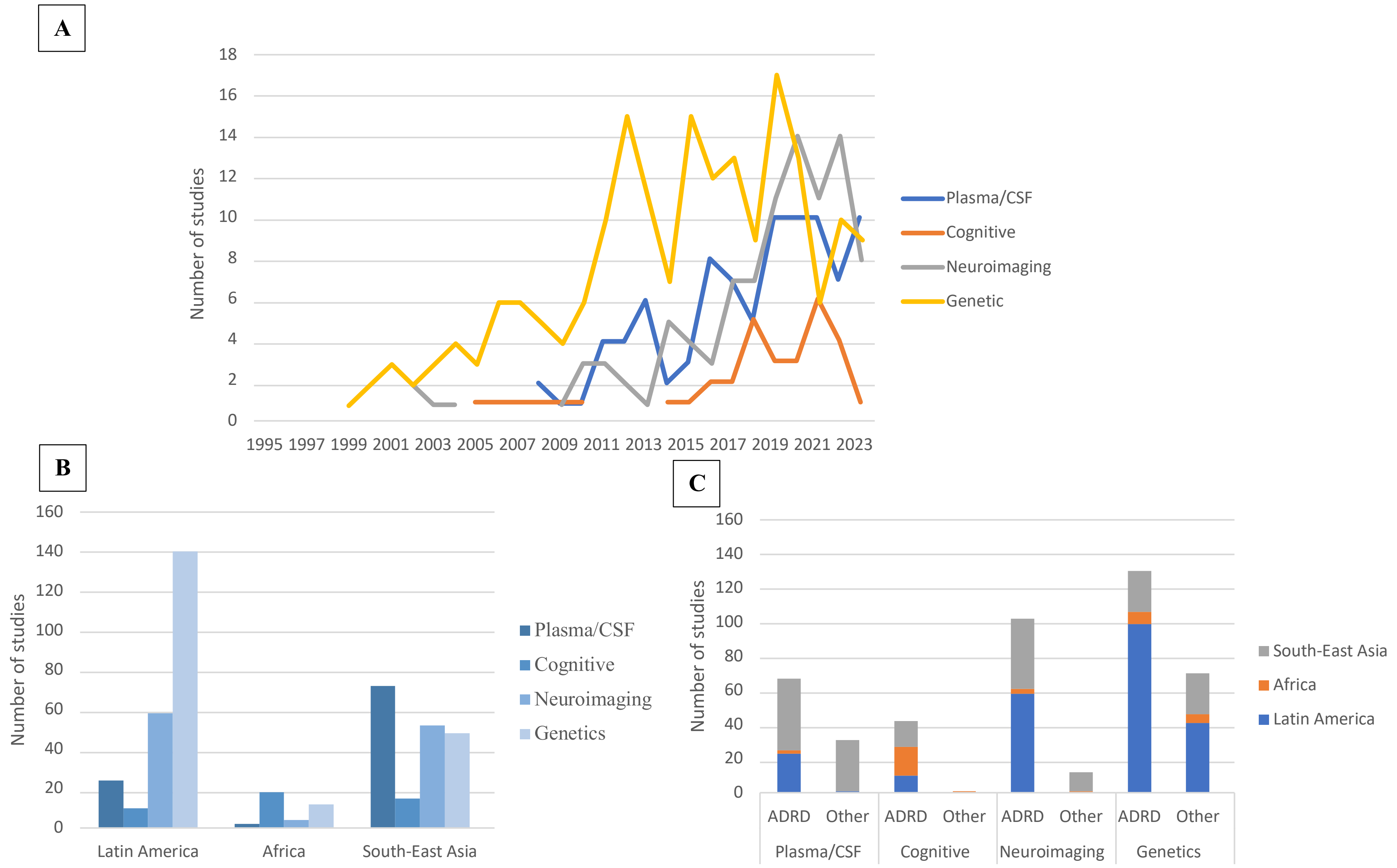

Supplementary Figure 1
Descriptive summary of the studies included in the selective review. (A) Trend in number of studies published by biomarker type across the Global South. (B) Bar graph shows number of studies published by biomarker type, broken down by regions in the Global South. (C) Stacked bar graph shows the breakdown of ADRD studies compared to other neurodegenerative conditions per biomarker type across three regions in the Global South. ADRD studies include AD, MCI, VAD, FTLD, DLB. Other conditions include PD, HD, ALS.

**Supplementary Table 1: Fluid (Plasma/CSF) Studies in Latin America**							
**Study**	**Regions/ Country/ Population**	**Sample disease (mean age)**	**Biomarker** (type, markers)	**Cognitive measures**	**Brain measures**	**Biomarker results**	**Multimodal results**
				**CSF**			
Allegri et al (2021)	Latam/ Argentina/ Argentinean	43 AD (72.8) 18 HC (69.2) 89 MCI (72.8) Follow up 30 months	CSF (Aβ1-42, t-Tau, p-Tau181)	MMSE RAVLT - free recall and recognition BNT SVF PVF Digit span TMT A and B ROCFT - copy and delay FAQ	MRI FDG-PET PiB-PET	AD = MCI < HC in $A\beta_{1-42}$ AD > MCI = HC in t-Tau AD > HC in p-Tau181 AD < MCI < HC in $A\beta_{1-42}$/p-Tau181 18% HC, 64% MCI and 92% AD were amyloid positive AD < MCI < HC Right hippocampal volumen 20% HC, 53% MCI and 80% AD abnormal FDG-PET 7% HC, 56% MCI and 78% AD positive PiB-PET AD < MCI < HC altered RAVLT free recall, BNT and SVF	10% of patients with abnormally low CSF Aβ1-42 levels show normal amyloid PET results After a 30-month follow-up, MCI progressed to dementia in 34.7% of patients. Progression to dementia was observed in 45% of amyloid-positive patients versus 20% of non-amyloid-positive patients.
Custodio et al (2022)	Latam/ Peru/ Peruvian	69 AD (74) 63 HC (76) 53 MCI (75)	CSF (Aβ1-42, t-Tau, p-Tau181)	RUDAS M@T FAQ	MRI		All CSF biomarkers correlated with medial temporal and parietal lobe atrophy and white matter hyperintensity.
Mendez et al (2018)	Latam/	14 AD (75.25) 15 HC (66.7) 27 MCI (73.12)	CSF ($A\beta_{1-42}$, t-Tau, p-Tau181)	MMSE ADAS MoCA Clock Drawing Logical Memory - immediate and delayed recall	MRI PiB-PET	18.2% HC, 50% MCI and 88.9% AD were CSF biomarkers positive 13.3% HC, 45.5% MCI and 84.6% AD were PiB-PET positive 20% HC, 48% MCI and 92.3% AD were biomarker positive	One-year conversion rate to dementia was 20% in the MCI cohort At the 1-year follow-up, dividing each group and conducting cognitive testing based on biomarkers (positive vs. negative) had no significant impact.

	Argentina/ Argentinean			RAVLT - immediate recall, delayed recall and recognition<br>BNT<br>SVF<br>PVF<br>TMT A and B<br>FAQ		AD < MCI < HC altered RAVLT immediate recall, delayed recall and recognition, SVF and MoCA<br>The FAQ and CDR functional scales revealed significant deterioration in AD, with no notable decline observed in other tests, except for SVF	
Niikado et al (2018)	Latam/ Argentina/ Argentinean	11 HC (63)<br>12 MCI (69.5)<br>14 AD (71)<br>13 bvFTD (58)<br>4 nfvPPA (60)<br>2 svPPA (61)<br>6 lvPPA (62)	CSF (A$\beta_{1\text{-}42}$, t-Tau, p-Tau181, NfL)	MMSE	MRI	MCI < HC and AD < MCI in A$\beta_{1\text{-}42}$<br>HC < AD in t-Tau<br>HC < MCI = AD in p-Tau181<br>HC < MCI < AD < bvFTD in NfL<br>ROC curve NfL<br>HC vs bvFTD: AUC=0.944, 84.62% sensitivity, 90.91% specificity<br>HC vs AD: AUC=0.922<br>bvFTD vs AD: AUC= 0.736	A marginally significant negative correlation between NfL levels and left orbitofrontal cortical thickness in the FTD group
Radanovic et al (2019)	Latam/ Brazil/ Brazilian	54 HC (71.3)<br>82 MCI (72.8)<br>46 AD (73.4)<br>26 OD (70.8)	CSF (A$\beta_{1\text{-}42}$, t-Tau, p-Tau181)	MMSE<br>FOME<br>RBMT<br>TMT A and B<br>Revised Wechsler Adult Intelligence Scale Vocabulary<br>Block Design subtests<br>IQCODE	-	AD < MCI = HC in A$\beta_{1\text{-}42}$<br>HC = MCI < AD in t-Tau<br>AD > HC, AD > MCI and AD > FTD in p-Tau181<br>AD < MCI = HC in A$\beta_{1\text{-}42}$/p-Tau181	MMSE correlated weakly with all CSF biomarkers in the total sample<br>A$\beta_{1\text{-}42}$ and MMSE correlated weakly in MCI, and moderately in OD<br>t-Tau showed a mild inverse correlation with MMSE in HC and MCI, and a moderate/strong correlation in OD<br>p-Tau181 correlated with MMSE in AD (weak correlation) and OD (moderate correlation)<br>A$\beta_{1\text{-}42}$/p-Tau181 ratio had a moderate to strong correlation with MMSE in OD

Reis et al (2012)	Latam/ Brazil/ Brazilian	8 HC (70.7)<br>12 AD (72.1)	CSF (Aβ_{1-42}, t-Tau, p-Tau181)	MMSE	-	AD < HC in Aβ_{1-42}<br>AD > HC in t-Tau<br>AD < HC in Aβ_{1-42}/p-Tau181<br>AD < HC in Aβ_{1-42}/t-Tau<br>AD < HC in MMSE	A significant and moderate correlation was found for the Aβ42 and MMSE (positive correlation)<br>A significant and moderate negative correlation was found between the t-Tau and MMSE.
Ruso et al (2016)	Latam/ Argentina/ Argentinean	15 HC (64.9)<br>28 MCI (70.1)<br>13 AD (73.1)	CSF (Aβ_{1-42}, t-Tau, p-Tau181)<br>APOE genotyping	MMSE<br>Logical Memory - immediate and delayed recall<br>MoCA<br>Clock Drawing and Coping<br>RAVLT - immediate recall, delayed recall and recognition<br>False Alarms<br>BNT<br>SVF<br>PVF<br>TMT A and B<br>FAQ	MRI<br>FDG-PET<br>PiB-PET	AD < HC and AD < MCI in Aβ_{1-42}<br>AD > HC and AD > MCI in t-Tau<br>14.2% HC, 50% MCI and 83.3% AD were PiB-PET positive<br>AD < MCI < HC in MoCA<br>AD < HC = MCI in Clock Drawing and Copying<br>AD < HC = MCI in FAQ<br>AD = MCI < HC in volumetric MRI	Neuropsychology, structural, and molecular measures showed a continuum between the three groups, with AD being the worst group, HC better, and MCI showing an intermediate status (long-delay recall, hippocampal volume, PiB-PET and CSF)<br>Amyloid deposition and hippocampal atrophy data highly correlated with the memory performance
Surace et al (2012)	Latam/ Argentina/ Argentinean	10 MCI (69)<br>7 AD (69)<br>3 FTD (60)	CSF (Aβ_{1-42}, t-Tau, p-Tau181)	MMSE<br>BNT<br>RAVLT<br>Signoret's logical memory	-	AD < FTD in Aβ_{1-42}<br>AD > FTD in t-Tau<br>FTD < AD in p-Tau181<br>AD < FTD in Aβ_{1-42}/p-Tau181<br>MCI < AD = FTD in MMSE	-
Fraga et al (2019)	Latam/	36 HC (68.1)<br>32 AD (70.7)<br>30 bvFTD (68.7)	CSF (AnxA1, LX4A)<br>Plasma or serum (hsCRP, IL-1β, TNF,	MMSE	-	AD < HC and AD < bvFTD in plasma hsCRP and TNF<br>AD > HC in plasma TGF-β1<br>AD > bvFTD in plasma LXA4	-

	Brazil/ Brazilian		TGF-β1, AnxA1, LXA4)			No difference for AnxA1 and LAX4 was observed between AD and bvFTD in CSF	
Clarens et al (2020)	Latam/ Argentina/ Argentinean	141 MCI<br>58 Aβ- (68.5)<br>83 Aβ+ (69.4)	CSF (Aβ_{1-42}, t-Tau, p-Tau181)	MMSE<br>Logical Memory - immediate and delayed recall<br>RAVLT - immediate recall, delayed recall and recognition<br>ROCFT - copy and delay<br>BNT<br>SVF<br>PVF<br>Digit span - forward and backward<br>Digit symbol<br>TMT A and B<br>FAB	FDG-PET<br>PiB-PET	MCI was separated according to presence or absence of amyloid, identified by positive PIB-PET biomarker findings, or low CSF Aβ_{1-42} levels	Worse performance in memory tests in MCI with positive amyloid markers.
Harris et al (2015)	Latam/ Argentina/ Argentinean	8 HC (60.75)<br>23 MCI (65.88)<br>2 AD (81)	CSF (Aβ_{1-42}, t-Tau, p-Tau181)	MMSE<br>TAP-BA<br>CRQ<br>Logical Memory<br>ADAS-Cog<br>RAVLT<br>MoCA<br>Clock Drawing<br>PVF<br>BNT<br>TMT A and B	-	-	The CRQ had a positive correlation with TAP-BA, education, and Aβ_{1-42}<br>Inverse correlations were found between CRQ and TMT A and TMT B<br>Aβ_{1-42} significantly correlated with t-Tau, PVF and RAVLT<br>After dividing the MCI according to Aβ_{1-42} levels, higher levels of CSF Aβ_{1-42}, were associated with higher cognitive reserve

Mirandez et al (2017)	Latam/ Brazil/ Brazilian	37 HC (72.5) 30 MCI (74.5)	CSF ($A\beta_{1-42}$, t-Tau, p-Tau181)	VF - animal, fruits, and means of transportation FAS FAS-COWA		MCI: 57.9% abnormal $A\beta_{1-42}$, 42.1% abnormal p-Tau181, 47.3% abnormal t-Tau and 42.1% abnormal $A\beta_{1-42}$/p-Tau181	MCI: a significant negative correlation between t-Tau and animal fluency. $A\beta_{1-42}$, P-tau, and $A\beta_{1-42}$/p-Tau181 levels did not correlate with performance in VF tasks
Rizzi et al (2018)	Latam/ Brazil/ Brazilian	12 SCI (63.5) 33 MCI (68)	CSF ($A\beta_{1-42}$, p-Tau181)	VM-CERAD Clock Drawing	-	SCI > MCI in $A\beta_{1-42}$ SCI < MCI in p-Tau181/$A\beta_{1-42}$ SCI > MCI in VM-CERAD $A\beta_{1-42}$ ROC: AUC= 0.768, 66.7% sensibility, 75% specificity p-Tau181/$A\beta_{1-42}$ ROC: AUC= 0.742 60.6% sensibility, 75% specificity	
**Plasma/Serum/PBMC**							
Castillo-Mendieta et al (2021)	Latam/ Mexico/ Mexican	51 HC (80.5) 24 SCI (78.0) 26 MCI (82.08)	Plasma ($A\beta_{1-42}$, t-Tau, p-Tau181)	MMSE	-	HC < MCI in $A\beta_{1-40}$ HC > SCI = MCI in $A\beta_{1-42}$ HC > SCI = MCI in $A\beta_{1-42}$/$A\beta_{1-40}$ HC > SCI = MCI in t-Tau HC < SCI = MCI in p-Tau181	A significant correlation between the $A\beta_{1-42}$/$A\beta_{1-40}$, p-Tau and p-Tau/t-Tau and both MCI and SCI
Gongora-Rivera et al (2020)	Latam/ Mexico/ Mexican	49 HC (72.85) 29 AD (75.34)	Plasma (CTACK, MIG, SDF-1α)	MMSE	-	HC < AD in CTACK, MIG, SDF-1α levels	Levels of CTACK, MIG and SDF-1α were negatively correlated with MMSE
Quiroz et al (2020)	Latam/ Colombia/ Colombian	1070 carriers PSEN1 E280A mutation (30, 8-73)	Plasma (NfL)	MMSE CERAD word list delayed recall Functional Assessment Staging Test		Carriers > Non-carriers in NfL	

		1074 non-carriers (29, 8-75)					
Ramirez-Cuapio et al (2021)	Latam/ Mexico/ Mexican	19 HC (73.6)<br>28 AD (74.7)	Serum (REST)	MMSE	MRI	Mean serum REST levels did not differ between HC and AD	
Rocha et al (2012)	Latam/ Brazil/ Brazilian	19 HC (72.47)<br>19 AD (77.05)	PBMC<br>(IL-1β, IL-6, IL-10, TNFα, IFNγ)	MMSE	-	HC showed increased baseline secretion of all cytokines, but these concentrations remained statistically unchanged after treatment with βA peptide.<br>AD > HC in cytokine levels	
Sekler et al (2008)	Latam/ Chile/ Chilean	29 HC (70.7)<br>59 AD (76.4)	Plasma<br>(FRAP, MDA)	MMSE<br>FCSRT<br>BNT<br>FAB<br>SVF<br>PVF	-	No differences between HC and AD in FRAP and MDA	FRAP was positively correlated with MMSE
Slachevsky et al (2017)	Latam/ Chile/ Chilean	37 HC (71.3)<br>53 AD (73.62)	Platelet<br>(HMWtau, LMWtau)	MMSE<br>ACE-R<br>MoCA<br>BNT<br>PVF<br>SVF<br>Digit Span<br>FCSRT<br>Short Recognition Memory Test for Faces of the Camden Memory Tests	MRI	HC < AD in HMW/LMW tau ratio<br>LMW tau and HMW tau levels were not statistically different between HC and AD<br>HC > AD in MMSE, MoCA and ACE-R	The HMW/LMW tau ratio correlated with performances in ACE-R, MOCA, and MMSE<br>There were no associations between HMW and LMW tau and MRI structures

				FAB<br>MCST<br>TMT A and B<br>T-ADLQ			
Villarreal et al (2016)	Latam/<br>Panama/<br>Panamanian	77 HC (76.5)<br>30 MCI (81.2)<br>28 AD (81.9)	Serum<br>(24 proteins)	MMSE<br>Clock Drawing	-	HC > AD in IL-18<br>HC < AD and HC < MCI in I309<br>HC > MCI > AD in MMSE and Clock Drawing	Random forest analysis revealed that within the AD, the biomarker profile ROC curve with AUC of 0.94, 86% sensitivity and 90% specificity
O'Bryant et al (2013)	Latam/<br>Mexico/<br>Mexican	314 HC (65)<br>49 AD (76.5)	Serum<br>(100 proteins)	MMSE	-	Random forest predicted 30 markers, 22 overexpressed and 8 under-expressed.<br>HC > AD in MMSE	ROC curve: AUC= 0.77, 92% sensitivity and 64% specificity
Honig et al (2023)	Latam/<br>Caribbean/<br>Dominican Republic	592 HC (69.7)<br>154 AD (76.4)	CSF and Plasma<br>($A\beta_{1-42}$, t-Tau, p-Tau181,GFAP, NfL)			Associations between plasma and CSF levels for p-Tau181, NfL, and p-Tau181/$A\beta_{1-42}$<br>Clinical AD had plasma biomarkers in 54.6% p-Tau181 and 41.1% in pTau181/$A\beta_{1-42}$<br>Increased plasma GFAP among individuals with and without dementia who had plasma p-Tau181 or p-Tau181/$A\beta_{1-42}$ positive<br>Increased plasma NfL among individuals with and without dementia who had plasma p-Tau181 positive	ROC curve p-Tau181: AUC= 0.86, 89% sensitivity,78% specificity<br>ROC curve p-Tau181/$A\beta_{1-42}$: AUC= 0.86, 94% sensitivity, 75% specificity
Salas-Leal et al (2021)	Latam/<br>Mexico/<br>Mexican	88 HC (70.14)<br>88 PD (70.47	Plasma<br>(α-syn)<br>SNCA rs356219	MMSE		HC > PD in SNCA mRNA levels<br>PD SNCA alelle G had lower mRNA level<br>HC < PD in plasma α-syn<br>PD SNCA allele G had higher plasma α-syn	ROC curve α-syn: AUC= 0.693, 66.7% sensitivity, 63.9% specificity

Supplementary Table 2: Cognitive Studies in Latin America							
**Study**	**Regions/ Country/ Population**	**Sample disease (mean age)**	**Biomarker** (type, markers)	**Cognitive measures**	**Brain measures**	**Biomarker results**	**Multimodal results**
de Araujo et al (2018)	Latam/ Brazil/ Brazilian	65 HC (72.83)<br>70 AD (78.87		MMSE<br>RUDAS		HC < AD in age<br>HC > AD in MMSE and RUDAS	ROC curve RUDAS: AUC= 0.87, 81.54% specificity, 76.1% specificity<br>ROC curve MMSE: AUC= 0.84, 72.3% specificity, 78.9% specificity<br>Low education < 8 years<br>ROC curve RUDAS: AUC= 0.82, 67.7% specificity, 79% specificity<br>ROC curve MMSE: AUC= 0.75, 74.19% specificity, 60.53% specificity<br>High education ≥ 8 years<br>ROC curve RUDAS: AUC= 0.92, 91.2% specificity, 81.8% specificity<br>ROC curve MMSE: AUC= 0.97, 94.12% specificity, 87.88% specificity
Maito et al (2023)	Latam/ Argentina, Chile, Colombia, Peru, Mexico	Argentina: 192 HC, 257 AD, 53 FTD<br>Chile: 145 HC, 197 AD, 59 FTD<br>Colombia: 232 HC, 320 AD, 155 FTD<br>Mexico: 21 HC, 30 AD, 7 FTD<br>Peru: 16 HC, 100 AD, 8 FTD		MMSE<br>MoCA<br>ACE-III<br>IFS<br>FAQ<br>NPI<br>Mini-SEA		The best discriminators of AD vs. HC were Mini-SEA, NPI, MMSE and IFS and, to a lesser degree, age, education and sex<br>The best discriminators of FTD vs. HC were NPI, Mini-SEA, IFS, age, education, MMSE and sex	The best model for discriminating between AD and FTD patients was the Random Forest model using the following variables: Mini-SEA, NPI, MMSE, age, IFS, education, and sex<br>ROC curve: AUC=0.965, 75% sensitivity, 97.2% specificity

Núñez-Fernández et al (2022)	Latam/<br>Mexico/<br>Mexican	117 HC (78)<br>117 AD (78)		ROCF- Copy and Recall<br>Stroop - word, color, word-color, interference<br>M-WCST - categories, perseveration errors, total errors<br>TMT A and B<br>BTA<br>VFT - letters F, A, S and M, animals, fruits, occupations<br>BNT<br>SDMT<br>HVLT-R - total learning, delayed recall, recognition		There were significant differences between AD and HC in all direct scores of the neuropsychological tests except for M-WCST Total Error scores	AUCs for each cognitive domain showed a moderate–high degree of accuracy in discriminating between AD and HC in each cognitive domain<br>Executive Function: AUC= 0.738<br>Attention and Processing Speed: AUC= 0.880<br>Language: AUC= 0.895<br>Learning and Memory: AUC= 0.944
Russo et al (2018)	Latam/<br>Argentina/<br>Argentinean	15 HC (64.9)<br>28 MCI (70.1)<br>13 AD (73.1)		MMSE<br>CDR<br>RAVLT - delayed recall<br>BNT<br>TMT A and B<br>ECog<br>FAQ		There were no statistically significant difference on MMSE, BNT or TMT A among HC, MCI and AD<br>HC, MCI and AD differed on RAVLT and TMT B<br>HC < MCI < AD in ECog<br>HC < MCI and HC < AD in FAQ	The ECog scale showed a strong correlation with the FAQ score<br>ROC curve ECog MCI vs AD: AUC= 0.81, 84% sensitivity, 60% specificity<br>ROC curve ECog HC vs AD: AUC= 0.99, 99% sensibility, 82% specificity<br>ROC curve ECog HC vs MCI: AUC= 0.96, 90% sensitivity, 80% sensitivity
Custodio et al (2021)	Latam/<br>Peru/<br>Peruvian	33 AD (72.21)<br>bvFTD (64.28)		CDR<br>ACE-III<br>IFS<br>Mini-SEA<br>FBI<br>IRI-EC		bvFTD < AD in ACE-III and IFS<br>bvFTD < AD in Mini-SEA, FBI, IRI-EC, IRI-PT, and r-SMS	ROC curve ACE-III: AUC= 0.85, 67% sensitivity, 94% specificity<br>ROC curve IFS: AUC= 0.78, 76% sensitivity, 67% specificity<br>ROC curve Mini-SEA: AUC= 0.96, 100% sensitivity, 83% specificity

				IRI-PT<br>r-SMS			ROC curve combined ACE-III, IFS and Mini-SEA: AUC= 0.96, 89% sensitivity, 100% specificity
Custodio et al (2020)	Latam/<br>Peru/<br>Peruvian	64 HC (68.92)<br>60 MCI (68.77)<br>63 Dem (72.69)		MMSE<br>IFS<br>RUDAS		HC > MCI > Dem in MMSE, IFS and RUDAS	ROC curve RUDAS HC vs MCI: AUC= 0.98, 89% sensitivity, 93% specificity<br>ROC curve IFS HC vs MCI: AUC= 0.99, 100% sensitivity, 93% specificity<br>ROC curve MMSE HC vs MCI: AUC= 0.85, 87.5% sensitivity, 65% specificity
Rodriguez-Salgado et al (2022)	Latam/<br>Cuba/<br>Cuban	53 HC (70.4)<br>46 MCI (72.7)<br>47 Dem (74.1)		CDR<br>MoCA<br>Fluency - animal<br>BHA - favorites, match, line orientation threshold		Significant differences among HC, MCI and Dem groups in CDR, MoCA, fluency and BHA	ROC curves BHA HC vs MCI and Dem: AUC= 0.949, 91% sensitivity, 85% specificity<br>ROC curves BHA HC vs Dem: AUC= 0.977, 96% sensitivity, 98% specificity<br>ROC curves BHA HC vs MCI: AUC= 0.942, 92% sensitivity, 83% specificity
Bruno et al (2017)	Latam/<br>Argentina and Chile	139 HC (68.03)<br>70 AD (76.79)<br>31 bvFTD (68.06)		ACE-III		HC > bvFTD > AD in ACE-III<br>HC > bvFTD > AD in attention, memory and verbal fluency domains<br>HC > AD and HC > bvFTD in language domain<br>HC > AD and bvFTD > AD in visuospatial ability	ROC curves ACE-III HC vs AD: AUC= 0.98, 98.5% sensitivity, 82% specificity<br>ROC curves ACE-III HC vs bvFTD: AUC= 0.90, 93.5% sensitivity, 77.7% specificity<br>With a cutoff point of 86, 98.6% AD, 83.9% bvFTD and 84.2% HC were correctly classified.
Gil et al (2014)	Latam/<br>Colombia/<br>Colombian	84 HC (68)<br>26 MCI (65)<br>83 Dem (73)		MoCA<br>MMSE<br>VFT<br>BNT<br>Word list - memory, recall, recognition<br>Constructional Praxis<br>FCSRT<br>Digit Symbol		HC > MCI > Dem in MoCA	ROC curves MoCA HC vs MCI and Dem: AUC= 0.85, 89% sensitivity, 79.8% specificity

				IFS			
Bahia et al (2018)	Latam/<br>Brazil/<br>Brazilian	15 HC (66.27)<br>20 AD (71.8)<br>18 bvFTD (70.17)		MMSE<br>ACE-R<br>CDR<br>NPI<br>IFS<br>FAB<br>RAVLT<br>VR<br>VFT<br>Stroop test		AD and bvFTD presented worse performance than HC in episodic memory, verbal fluency, and inhibitory control.<br>AD had lower scores than bvFTD in the delayed recall of both episodic memory tests.<br>HC > AD = bvFTD in IFS<br>HC > bvFTD in FAB	IFS strongly correlated with MMSE, ACE-R, Stroop, semantic VF, phonemic VF, RAVLT total and delayed recall.<br>ROC curves IFS HC vs AD: AUC= 0.82, 80% sensitivity, 70% specificity<br>ROC curves IFS HC vs bvFTD: AUC= 0.71, 73% sensitivity, 61% specificity<br>ROC curves FAB HC vs AD: AUC= 0.71, 67% sensitivity, 70% specificity<br>ROC curves FAB HC vs bvFTD: AUC= 0.72, 67% sensitivity, 67% specificity

Supplementary Table 3: Neuroimaging Studies in Latin America							
**Study (doi)**	**Regions/ Country/ population**	**Sample disease (mean age)**	**Biomarker (type, markers)**	**Cognitive measures**	**Brain measures**	**Biomarker results**	**Multimodal results**
10.1176/appi.neuropsych.17120355	Argentina	27 offSpring of LOAD (55)<br>18 unrelated HC (51)	**Imaging**<br>MRI<br>PET	WAT-BA<br>BDI-II RAVLT Delayed<br>LASSI-LB1 cued recall<br>Cued intrusions<br>B1 cued recall, intrusions<br>B2 cued recall, intrusions<br>A3 intrusions, cued recall, intrusions<br>Delayed Recall<br>Delayed Intrusions	Volume<br>Amyloid load	-	Recovery from proactive semantic interference (frPSI)and RAVLT delayed recall were lower in LOAD cases;<br><br>Cortical Thickness and PIB load associations were different in HC and OLOAD, involving brain regions concerned with autonomic, motor, and motivational control AD-related regions.
10.1002/alz.12906	Colombian	24 Non-demented PSEN-1 E280A carriers<br><br>20 non-carrier family members	**Imaging**<br>MRI<br>PET<br><br>Plasma<br>Ptau217	word list delayed recall	Volume<br>Amyloid load<br>Tau load	-	PSEN1 E280A carriers have higher plasma p-tau217;<br><br>Higher baseline p-tau217 is associated with greater future amyloid and tau (PET) pathologies;<br><br>Higher baseline p-tau217 is associated with worse future memory performance.
10.3233/JAD-210185	Colombia	33 CU PSEN-1 E280A mutation carriers (32.56); 41 non-carriers(32.18)	**Imaging**<br>PET	CDR<br>MMSE FAST<br>GAI	Amyloid load<br>Tau load	CU PSEN-1 E280A mutation carriers have higher amyloid and tau PET load;	Neuroticism was positively correlated with entorhinal tau levels only in carriers, but not with amyloid levels.
10.1017/s1355617720000673	Colombia	Colombian kindred with the Presenilin 1 (PSEN1) E280A ADAD mutation (19 carriers and 26 noncarriers) c	**Imaging**<br>PET	Rey–Osterrieth Complex Figure immediate recall test(ViM);	Amyloid and Tau LoAD;	CU carriers have higher load of PIB and FTP;	CU carriers and noncarrierdod not differ on ViM performance.<br><br>Moderate correlation between regional tau and ViM performance;<br><br>No correlations were observed in noncarriers;

							AD pathology and greater age are associated with worse ViM performance in ADAD before the onset of clinical symptoms
10.1590/1516-4446-2020-1503	Brazil	30 CU (> 60)	**Imaging**<br>PET	MMSE;<br><br>Pittsburgh Sleep Quality Inventory;<br><br>Epworth Sleep Scale	Amyloid Load		Longer times in bed ($p = 0.024$) and reduced sleep efficiency ($p = 0.05$) in individuals with positive amyloid;<br><br>multiple-domain mild cognitive impairment (MCI) had shorter self-reported total sleep times ($p = 0.034$) and worse overall sleep quality ($p = 0.027$) compared to those with single-domain MCI.
10.47626/1516-4446-2021-2374	Brazil	35 AD (74.08)<br>43 MCI (72.58)<br>26 HC (32)	**Imaging**<br>PET<br>MRI	MMSE<br>AVLT<br>SKT<br>Blessed<br>IQ-CODE	Amyloid load<br><br>Glucose metabolism<br><br>Volume	Amyloid load AD > MCI > CU	Significant HSV reductions were found in A+(N)+ subjects in the presubiculum/subiculum complex and molecular layer, related to worse memory performance;<br><br>A+(N)+ and A+(N)− categories, subicular volumes were inversely correlated with the degree of Aβ deposition;<br><br>HSV reductions are larger both in A+(N)+ and A+(N)− subjects in direct proportion to the degree of Aβ deposition.
10.47626/1516-4446-2021-2374	Brazil	27 AD (74)<br>39 MCI (73)<br>24 HC (71)	**Imaging**<br>PET<br>MRI	Blessed Scale<br>MMSE	Amyloid load<br>Glucose metabolism<br>Volume	PIB positive associated to AD<br><br>FDG positive (hypometabolism) associated AD<br><br>lower early-phase 11C-PiB uptake in limbic structures than 18F-FDG uptake.	Early-phase 11C-PiB appears to provide different information from 18F-FDG about neurodegeneration.

10.1016/j.bandc.2021.105749	Brazil	18 CU<br>30 MCI<br>23 AD When compared with the analyses.	**Imaging**<br>PET<br>MRI	RAVLT and SKT memory tests, the STMB was the only cognitive task that differentiated groupsdivided by ATN status. regression	Volume<br>Amyloid load<br>Glucose metabolism	Higher proportion of A+ in AD and MCI;<br><br>Higher proportion of (N+) in AD	The STMB test showed to be sensitive to the signs of AD pathology and may represent a cognitive marker within the AD continuum.
10.1007/s11307-021-01660-7	Brazil	10 AD (78.2)<br><br>10 SuperAgers-SA (82.3)<br><br>10 HC (83.5)<br>10 middle-aged controls- MC(58.7)		RAVLT DR MMSE		AD group exhibited a increased Aβ load compared to the MC, but not HC.	SuperAgers exhibited a similar Aβ load to AC and MC, differing in cognitive performance.
10.1016/j.pscychresns.2021.111342	Argentina	30 Offspring of LOAD – (O-LOAD- 54.4)<br><br>25 HC (51)	**Imaging**<br>PET<br>MRI	Design Fluency<br><br>Stroop Color Test<br><br>Word Test<br><br>Trails Making Test-B<br><br>Tower of London	Cortical thickness<br><br>Amyloid Load	SUVRs and Volumes not disclosed, only correlations	Greater orbitofrontal thickness correlated with better design fluency in O-LOAD.<br><br>Amyloid load in precuneus correlated with worse executive performance in O-LOAD.<br><br>Greater right rostral midfrontal cortex thickness correlated with stroop in O-LOAD.<br><br>Lower thickness in cingulate and orbitofrontal areas correlated with TMTB in O-LOAD.
10.1016/j.jpsychires.2018.10.008	Argentina	30 Offspring of LOAD – (O-LOAD- 54.4)<br><br>25 HC (51)	**Imaging**<br>PET<br>MRI		Cortical thickness<br><br>Amyloid load<br><br>Glucos Metabolism	O-LOAD has:<br>isocortical thinning;<br><br>FDGhypermetabolismoverlapping with the areas of cortical thinning;<br><br>Higher amyloid load in the temporoparietal cortex, postcentral gyrus, fusiform inferior and middle temporal and lingual gyri	

10.1007/s00259-007-0523-1	Argentina	10 FTD (66)<br>17 AD (64)<br>8 HC ma (50)<br>5 HC (67)<br>3 HC Young (21)	**Imaging**<br>PET	MMSE<br><br>Verbal fluency<br>Categories<br>Letters (FAS)<br>Visuospatial function<br>Clock drawing<br>Blockdesign<br><br>Verbal episodic memory<br>Immediate recall<br>Delayed recall	Amyloid load	FTD patients showed significantly lower PIB retention compared to AD in frontal, parietal temporal and occipital cortices as well as in putamina (p < 0.0001).	FTD patients showed significantly lower PIB retention compared to AD in frontal, parietal temporal and occipital cortices as well as in putamina (p < 0.0001).
10.1590/1516-4446-2017-0002	Brazil	17 AD (74.9)<br>19 HC (73.5)	**Imaging**<br>PET	MMSE<br>CDR<br>Blessed	Amyloid load	Voxel-based analysis was performed with statistical parametric mapping.<br><br>Higher PIB uptake in the AD group relative to controls throughout the cerebral cortex;<br><br>Decreased PIB uptake in white-matter	Higher PIB uptake in the AD group relative to controls throughout the cerebral cortex;<br><br>Decreased PIB uptake in white-matter
10.1590/1516-4446-2016-2083	Brazil	20 AD (75.5)<br>18 HC (72.7)	Imaging<br>MRI<br>PET<br>SPECT	BNT<br>DRS<br>FAQ<br>HVOT MMSE<br>Mini-Mental<br>RAVLT ROCF<br>Trail-A and Trail-B<br>WCST<br>WMS-R-LM WMS-R-VR	Volume<br>Amyloid load<br>CBF		FDG-PET and rCBF-SPECT more accurately identified patients with AD than MR

10.1016/s1474-4422(12)70227-2	Colombia	11 symptomatic PNSE1 E280A mutation (47.5)<br><br>19 presymptomatic PNSE1 E280A mutation carriers (32.6)<br><br>20 asymptomatic non-carriers(33.9)	**Imaging**<br>PET	MMSE<br>CDR	Amyloid load	Greater florbetapir binding in asymptomatic PSEN1 E280A mutation carriers than in age matched non-carriers;<br><br>Aβ began to accumulate mutation carriers at a mean age of 28·2 years, about 16 years and 21 years before the predicted median ages at mild cognitive impairment and dementia onset, respectively.	Greater florbetapir binding in asymptomatic PSEN1 E280A mutation carriers than in age matched non-carriers;<br><br>Aβ began to accumulate mutation carriers at a mean age of 28·2 years, about 16 years and 21 years before the predicted median ages at mild cognitive impairment and dementia onset, respectively.
10.1093/brain/awz150	Argentina	22 Young Control (37)<br>22 Old Control(73)<br>18 EOAD (63)<br>18 LOAD (70.6).	**Imaging**<br>PET	-	Amyloid load<br>Glucose metabolism	OC had a significant reduction of R1 and glucose uptake compared to YC predominantly at the dorsolateral and mesial frontal cortex;<br><br>EOAD and LOAD vs. OC showed a decreased R1 and glucose uptake at the posterior parietal cortex, precuneus, and posterior cingulum.<br><br>EOAD vs. LOAD showed a reduction in glucose uptake and R1 at the occipital and parietal cortex and an increased at the mesial frontal and temporal cortex. There was a mild increase in an amyloid deposition at the frontal cortex in LOAD vs. EOAD;<br><br>YC presented higherconnectivity than OC in R1 but lower connectivity considering glucose uptake.<br><br>EOAD and LOAD showed a decreased connectivity compared to controls that were more pronounced in glucose uptake than R1.	

10.1016/j.nicl.2021.102749	Colombian	11 symptomatic PNSE1 E280A mutation (47.5)<br><br>19 presymptomatic PNSE1 E280A mutation carriers (32.6)<br><br>20 asymptomatic non-carriers(33.9)	**Imaging**<br>PET	-	Amyloid load	Florbetapir and PiB cerebellar were significantly higher in carriers than non-carriers ($p < 0.0001$);<br>Cerebellar SUVR ponsbegan to distinguish carriers from non-carriers at age 34, 10 years before the carriers' estimated age at mild cognitive impairment onset;<br><br>Florbetapir and PiB cerebellar SUVR pons in carriers were positively correlated with age ($r = 0.44$ & 0.69, $p < 0.001$), cortical SUVR_pons ($r = 0.55$ & 0.69, $p < 0.001$), and negatively correlated with delayed recall memory ($r = -0.21$ & -0.50, $p < 0.05$, unadjusted for cortical SUVR_pons) and API ADAD composite ($r = -0.25$, $p < 0.01$, unadjusted for cortical SUVR_pons in florbetapir API ADAD cohort).	This PET study provides evidence of cerebellar Aβ plaque deposition in CU carriers starting about a decade before the clinical onset of ADAD.
10.1073/pnas.2113641119	Colombia	21 PSEN1 E280A mutation Carriers (35.99)<br><br>31 Noncarriers (35.59)	**Imaging**<br>MRI<br>PET	FAST<br>MMSE<br>CERAD delayed recall	Connectivity<br>Amyloid load<br>Tau load		Compared to non-carriers, mutation carriers exhibited less functional segregation and integration in poste-rior default-mode network (DMN) regions, particularly the precuneus, and in theretrospenial cortex, which has been shown to link medial temporal regions and corticalregions of the DMN. Mutation carriers also showed greater functional segregation andintegration in regions connected to the salience network, including the striatum andthalamus. Greater tau burden was associated with lower segregated and integrated func-tional connectivity of DMN regions, particularly the precuneus and medial prefrontalcortex. In turn, greater tau pathology was related to higher segregated and integratedfunctional connectivity in the retrospenial cortex and the anterior cingulate cortex, ahub of the salience network. Thesefindings enlighten our understanding of

							howAD-related pathology distinctly alters the brain's functional architecture in the preclini-cal stage, possibly contributing to pathology propagation and ultimately resulting in dementia.
10.1212/wnl.0000000000010177	Colombia	24 CU PSEN-1 E280A carriers (36.81)<br><br>28 noncarriers (37)<br><br>5 MCI (44)	**Imaging** PET	FCSRT	Amyloid load<br>Tau load	Free and total recall scores did not differ between cognitively unimpaired mutation carriers and noncarriers. Greater age predicted lower free recall and delayed free and total recall scores in carriers. In cognitively impaired carriers, delayed free recall predicted greater amyloid burden and entorhinal tau, while worse immediate free recall scores predicted greater tau in the inferior temporal and entorhinal cortices. In turn, in all carriers, lower free and total recall scores predicted greater amyloid and regional tau pathology.	FCSRT scores were associated with in vivo markers of AD-related pathology in cognitively unimpaired individuals genetically determined to develop dementia. Difficulties on free recall, particularly delayed recall, were evident earlier in the disease trajectory, while difficulties on cued recall were seen only as carriers neared the onset of dementia, consistent with the pathologic progression of the disease. Findings suggest that the FCSRT can be a useful measure to track disease progression in AD.
10.1002/alz.12248	Colombia	25 mutation PSEN-1 E280A carriers (19 CU/6 MCI)<br><br>19 non-carriers (age range: 28 to 49 years)	**Imaging** PET	MMSE<br>CERAD<br>FAST	Amyloid load<br>Tau load	Higher PIB in MCs<br><br>Higher FTP in MCs	NfL was related to tau pathology burden in preclinical AD;<br>Plasma NfL levels did not predict amyloid beta load in preclinical AD;

10.1186/s13195-019-0468-1	Colombia	Fourteen carriers (age = 28–42, Mini-Mental State Examination = 26–30) and 20 age-matched non-carriers	PET	CERAD Word List Learning	Amyloid load<br>Tau Load	Compared to non-carriers, mutation carriers had age-related elevations in both neocortical and striatal PiB binding;<br><br>n mutation carriers, PiB binding in both the neocortex and the striatum is related to entorhinal FTP;	Striatal PiB-PET measurements may offer particular value in the detection and tracking of preclinical ADAD.
10.1002/alz.12656	Colombia	27 carriers and 27 non-carriers of the presenilin-1 (PSEN1) E280A mutation	MRI<br>PET	CERAD) Word-List-Learning	Volume<br>Amyloid load<br>Tau Load	Locus coeruleus (LC) integrity declines 12 years before clinical onset in ADAD<br>LC integrity declines earlier and (ADAD) as compared to sporadic AD;<br><br>Reductions in LC integrity predict expansion of cortical tau in presenilin-1 (PSEN1) carriers<br>Lower LC integrity is associated with worse memory performance in PSEN1 carriers<br>Lower LC integrity is a promising early prognostic indicator in preclinical ADAD	LC integrity started to decline at age 32 in carriers, 12 years before clinical onset, and 20 years earlier than in sporadic AD. LC integrity was negatively associated with cortical tau, independent of amyloid beta, and predicted precuneus tau increases. LC integrity was positively associated with memory

10.1590/0004-282x20180025	Argentina	14 AD (75)<br>27 MCI (27)<br>15 HC (66)	**Imaging**<br>PET<br><br>**Fluid**<br>Aβ1-42<br>total tau<br>T181	MMSE<br>Logical<br>Logical delayed<br>CDR<br>ADAS –<br>ADAS –<br>Rey – immediate<br>Rey – delayed<br>Rey – delayed – recognition<br>MoCA<br>Fluency –<br>Fluency – Sem<br>Clock Design<br>Clock – Copy<br>TMT-A/B<br>FAQ<br>GDS<br>Boston	Amyloid load	PIB positivity at basline:<br>HC: 2/15<br>MCI:10/22<br>AD:11/13<br><br>High prevalence of positive AD biomarkers in the AD group, 92.3% (12/13);<br><br>low prevalence in the normal controls, 20%; almost half (48%) of the patients diagnosed with MCI had positive amyloid;<br><br>Follow-up (one year), the significant differences found at baseline on neuropsychological testing were similar at the follow-up assessment even though the AD group had significantly altered its functional performance (FAQ and CDR);<br><br>Semantic fluency showed greater impairment between the AD group and MCI and HC, respectively;<br>20% one-year conversion from MCI to dementia;	High prevalence of positive AD biomarkers in the AD group, 92.3% (12/13);<br><br>low prevalence in the normal controls, 20%; almost half (48%) of the patients diagnosed with MCI had positive amyloid;<br><br>Follow-up (one year), the significant differences found at baseline on neuropsychological testing were similar at the follow-up assessment even though the AD group had significantly altered its functional performance (FAQ and CDR);<br><br>Semantic fluency showed greater impairment between the AD group and MCI and HC, respectively;<br>20% one-year conversionfrom MCI to dementia;

http://doi.org/10.1002/mds.28373	Brazil	45 CBS (63)<br>Distributed into groups: likely related to AD (CBS FDG-AD) or likely non-AD (CBS FDG-nonAD) pathology	**Imaging**<br>PET	ACE-R<br>MMSE<br>BCSB<br>Verbal Fluency<br>Digit Span<br>NPI	Amyloid load<br><br>Glucose metabolism	The classification according to FDG-PET patterns in AD versus non-AD demonstrated 76.92% of sensitivity (confidence interval [CI] 46.19%–94.16%) and 100% of specificity (CI 80.49%–100%) to detect amyloid deposition on PIB-PET;<br><br>All patients classified as CBS FDG-AD tested positive at PIB-PET compared to 3 of 20 in the non-AD group;<br><br>The individual FDG-PET classification demonstrated 76.92% of sensitivity, 100% of specificity and positive predictive value and 88.5% of balanced accuracy to detect positive PIB-PET scans. Individuals with positive and negative;<br><br>PIB-PET showed hypometabolism in posterior temporoparietal areas and in thalamus and brainstem, respectively, mainly contralateral to most affected side, disclosing possible metabolic signatures of CBS variants.	CBS FDG-AD group demonstrated worse cognitive performances, mostly concerning attention, memory, visuospatial domains, and displayed more myoclonus and hallucinations;<br>The non-AD metabolic group presented more often limb dystonia, ocular motor dysfunction, motor perseveration, and dysarthria
10.1177/1533317515576387	Argentina	17 HC (63)<br>32 aMCI (69)<br>20 amMCI (70)<br>18 Non-memory MCI (67)<br>21 DA (70)<br>6 bvFTD (61)<br>11 PPA (61)<br>4 lPPA (67)<br>5 PCA (65)<br>2 AA (66)<br>7 Mixed Dem (70)<br>1 CBD (70)	**Imaging**<br>PET	MMSE<br>WMT<br>RAVLT<br>Boston naming test<br>Semantic and phonological fluency for language evaluation<br><br>Direct and reverse digit span<br><br>TMT<br><br>FAB	Amyloid load	Percentage of concordance between clinical diagnosis:<br><br>aMCI 68%<br>amMCI 60%<br>Non-memory MCI (66%)<br>DA (76%)<br>bvFTD (66%)<br>PPA (54%)<br>lPPA (100%)<br>5 PCA (100%)<br>AA (100%)<br>Mixed Dem (71%)<br>CBD (100%)	This study demonstrates that detecting in vivo amyloid plaques by molecular imaging is considerably frequent in most of the dementia syndromes and shows that there are frequent discordance between molecular diagnosis and clinical assumption.
10.1001/jamaneurol.2017.4907	Colombia	24 PSEN1 E280A kindred members (age range, 28-55 years), including 12 carriers, 9 of	**Imaging**<br>PET	MMSE<br>CERAD	Amyloid load<br>Tau load	β-Amyloid uptake levels were diffusely elevated in unimpaired carriers approximately 15 years prior to expected onset of MCIt.	β-Amyloid uptake levels were diffusely elevated in unimpaired carriers approximately 15 years prior to expected onset of MCIt.

		whom were cognitively unimpaired and 3 of whom had mild cognitive impairment, and 12 cognitively unimpaired noncarriers.				In carriers, tau load were associated with worse performance on the MMSE and CERAD .<br><br>medial temporal lobe load CU PSEN1 E280A mutation carriers in their late 30s;<br><br>Significant tau load in neocortical regions was observed in 1 cognitively unimpaired carrier as well as in those with mild cognitive impairment.	In carriers, tau load were associated with worse performance on the MMSE and CERAD .<br><br>medial temporal lobe load CU PSEN1 E280A mutation carriers in their late 30s;<br><br>Significant tau load in neocortical regions was observed in 1 cognitively unimpaired carrier as well as in those with mild cognitive impairment.
10.3233/jad-220075	Colombia	16 non-demented PSEN1 E280A ADAD mutation carries (40.1)<br><br>19 non-carrier family members (36.0)	Imaging<br>PET	OPID18<br>MMSE	Amyloid load<br>Tau load	Worse olfactory identification performance was related to worse MMSE scores (r = 0.55, p = 0.024) and CERAD delayed recall (r = 0.63, p = 0.007) and greater cortical amyloid (r = -0.53, p = 0.042) and tau load burden (entorhinal: r = -0.59, p = 0.016; inferior temporal: r = -0.52, p = 0.038).	Worse performance on olfactory identification was associated to AD pathology in ADAD mutation carriers
10.1016/j.nicl.2017.10.026	Brazil	20 AD (75)<br>18 HC (72	Imaging<br>MRI<br>PET<br>SPECT	-	Volume<br>Glucose metabolism<br>CBF<br><br>Multiple kernel learning (MKL), a sparse machine learning method, to identify of the most relevant ROIs for the classification.	the ROI-MKL approach resulted in better accuracies (with either atlas) for classification using 18F-FDG-PET (92.5% accuracy for ROI-MKL versus 84% for whole-brain), but not when using rCBF-SPECT or MRI.	The MKL-ROI approach highlights the high discriminative weight of a subset of brain regions

10.1186/s13195-020-00765-5	Colombia	14 PSEN1 E280A ADAD mutation carriers ()<br>15 non-carriers ()	Longitudinal Imaging<br>PET<br>MRI		Amyloid load<br>Tau load<br>Volume	Aβ load (16 years prior to expected symptom onset, EYO);<br><br>Tau load in entorhinal cortex (EC) tau (9 EYO), neocortical tau (6 EYO);<br><br>Hippocampal atrophy (6 EYO)<br><br>cognitive decline (4 EYO).	Rates of tau accumulation among carriers were most rapid in parietal neocortex (~ 9%/year). EC tau PET signal at baseline was a significant predictor of subsequent neocortical tau accumulation and cognitive decline within carriers.
10.1016/j.jpsychires.2019.12.019	Argentina	23 CU offspring of patients with Late-Onset Alzheimer's Disease (O-LOAD-)<br><br>22 HC ( )	**Imaging**<br>MRI<br>PET	MMSE<br><br>Semantic fluency (“animals” category)<br><br>phonetic fluency (words starting with letter P)<br><br>TMT A and B<br><br>Stroop Test<br><br>BDI-II<br><br>WAT-BA	Amyloid load<br>Glucose metabolism<br>Volume<br>Tractography	O-LOAD exhibited lower fiber density and fractional anisotropy in the posterior portion of the corpus callosum and right fornix;<br><br>In O-LOAD, reduced fiber density was associated with lower amyloid deposition in the right hippocampus, and greater cortical thickness in the left precuneus; higher mean diffusivity was related with greater cortical thickness of the right superior temporal gyrus.	Healthy offspring of late-onset AD (O-LOAD) patients show decreased connectivit;<br><br>Gray matter anomalies are associated to altered white matter integrity among O-LOAD;<br><br>White matter dysconnectivity involves right fornix and splenium of corpus callosum;<br><br>O-LOAD exhibited compromised DTI measures; Compromised white matter microstructure was associated with poorer semantic fluency.
10.1186/s13195-022-01030-7	Colombia	20 CU PSEN1 E280A mutation carriers<br><br>9 CI PSEN1 E280A mutation carriers<br><br>21 non-carriers<br><br>Mean age of participants was 35.8	**Imaging**<br>MRI<br>PET<br><br>**CSF**<br>Aβ42<br>p-tau	MMSE<br><br>FAST<br><br>CERAD<br><br>Word List Learning subtests	white matter hyperintensities (WMH)<br><br>Amyloid load	CU carriers had increased volume of WMH;<br><br>In mutation carriers, the volume of WMH strongly correlated with cognition;	WMH is increased among individuals with symptomatic autosomal-dominant Alzheimer’s disease, begins to increase prior to clinical symptom onset, and is an independent determinant of cognitive performance in this group.

10.3233/jad-200758	Brazil	38 AD<br>43 aMCI<br>27 CU	**Imaging**<br>PET	MMSE<br>Verbal and visual episodic memory tests	Amyloid load<br>FDG metabolism	A–(N)+ showed lower (p<0.043) verbal memory scores relative to A–(N)–;<br><br>Continuous Aβ measures were correlated with visual memory scores in the overall sample and when analyses were restricted to dementia or (N)+, but not in non-dementia or (N)–;	These results demonstrate that significant Aβ-cognition relationships are highly salient at disease stages involving neurodegeneration;<br><br>However, Aβ-cognition relationships during early AD stages may remain undetectable unless substantially large samples are evaluated.
10.1167/iovs.63.5.20	Brazil	11 AD (72.4)<br>21 MCI (72.5)<br>6 HC ()	**Imaging**<br>PET	RAVLT	Amyloid load<br>FDG metabolism	Diffuse color vision loss was found in individuals with signs of neurodegeneration (protan P = 0.002, deutan P = 0.003 and tritan P = 0.01), but not in individuals with signs of β-amyloid deposition only (protan P = 0.39, deutan P = 0.48, tritan P = 0.63), regardless of their clinical classification.	AD and MCI patients present acquired color vision deficiency that may be linked with impaired brain metabolism.
10.1186/s13195-020-00671-w	Colombia	35 CU PSEN1 mutation carriers (age range 26-41);<br><br>19 symptomatic carriers; 48 HC non-carriers (age range 27-44)	**Imaging**<br>PET	LAS-FNAME performance	Amyloid load<br>Tau load	CU mutation carriers had lower scores on the LAS-FNAME Total Scores (p = .040) compar to non-carriers;<br><br>Across all carriers higher amyloid load and regional tau in the entorhinal and inferior temporal cortex were associated with lower LAS-FNAME Total Scores.	Performance on the LAS-FNAME differentiated CU mutation carriers from non-carriers and was associated with greater amyloid and tau load when examining all carriers.
10.1371/journal.pone.0052859	Brazil	23 AD (76.3)<br>18 MCI (72.8)<br>17 HC (71.1)	**Imaging**<br>MRI	MMSE<br>Pfeffer<br>CDR<br>Clock Drawing Test<br>Verbal Fluency<br>CAMCOG<br>Boston Naming Test	Volume<br>DTI	After controlling for the gray matter atrophy, corpus callosum and uncinate fasciculus presented significantly lower anisotropic fraction (FA) in AD relative to HC	MCI and AD present microstructural WH damage.

10.3233/jad-221131	Argentina/Colombia	31 bvFTD<br>30 AD<br>37 HC	**Imaging**<br>MRI	Modified version of the Moral Sentiment Task	Volume	bvFTD patients exhibited greater impairments in self-conscious and other-oriented moral emotions as compared with AD patients and healthy controls;<br><br>In bvFTD patients, lower moral emotions scores were associated with lower gray matter volumes in caudate nucleus and inferior and middle temporal gyri;<br><br>In AD, these scores were associated with lower gray matter volumes in superior and middle frontal gyri, middle temporal gyrus, inferior parietal lobule and supramarginal gyrus.	bvFTD patients exhibited greater impairments in self-conscious and other-oriented moral emotions as compared with AD patients and healthy controls;<br><br>In bvFTD patients, lower moral emotions scores were associated with lower gray matter volumes in caudate nucleus and inferior and middle temporal gyri;<br><br>In AD, these scores were associated with lower gray matter volumes in superior and middle frontal gyri, middle temporal gyrus, inferior parietal lobule and supramarginal gyrus.
10.1590/s1516-44462011000200006	Brazil	70 AD (42)<br>16 MCI (42)<br>10 HC (10)	**Imaging**<br>MRI	MMSE<br>FAQ<br>CDR	Volume	Volumes of the cerebellar hemispheres, posterior cerebellar lobe, vermis and temporal lobe were found to be reduced as a function of the severity of the disease;<br><br>Positive correlations between the volume of the temporal lobe and cerebellum and the language, attention, and total scores	Reduced temporal lobe, posterior cerebellar lobe and vermal volume at baseline is a risk factor for the onset of dementia.

10.1016/j.pscychresns.2013.10.010	Brazil	22 AD (73.40)<br>26 HC (71.03)	**Imaging**<br>MRI	MMSE<br><br>RAVLT<br><br>Boston Naming test<br><br>Semantic verbal fluency<br><br>FAS<br><br>LNI<br><br>Forward digit span<br><br>Backward digit span<br><br>Tail making test-B<br><br>Stroop test<br><br>Clock Drawing test<br><br>Rey Complex figure	DMN, seed-based FC of the posterior cingulate was calculated;<br><br>Connectivity	We found a significant difference between patients with mild AD and controls in average z-scores: DMN, whole cortical positive (WCP) and absolute values. DMN individual values showed a sensitivity of 77.3% and specificity of 70%. DMN and WCP values were correlated to global cognition and episodic memory performance.	We showed that individual measures of DMN connectivity could be considered a promising method to differentiate AD, even at an early phase, from normal aging
10.1002/hbm.22248	Brazil	20 AD (73.8)<br>17 HC (72.3)	**Imaging**	MMSE<br>Pfeffer (FAQ)<br>CDR<br>RAVLT<br>NPI<br>VSF-LNI<br>BNT<br>Semantic VF<br>FAS<br>FAS<br>BDS<br>FDS<br>WAIS-R<br>TMT A-B<br>Stroop test<br>Clock drawing test	VBM<br>Connectivity	AD showed increased functional connectivity within the salience network (SN) compared with HC (right anterior cingulate cortex and left medial frontal gyrus), along with reduced functional connectivity in the DMN (bilateral precuneus);<br><br>Correlation between increased connectivity in Association between Anterior cingulate cortex and right insula areas of the SN and hyperactivity syndrome;	Association between specific network changes in AD and particular neuropsychiatric symptom types.

10.1017/s1355617709990956	Brazil	15 AD (74.2)<br>17 aMCI (68.2)<br>16 HC (69.1)	**Imaging**<br>MRI	RAVLT	VBM	Memory processes were compromised in aMCI and mild AD;<br><br>the same cerebral structures were involved in all RAVLT stages;<br><br>Learning and delayed recall were more related to the medial prefrontal cortex and hippocampi;<br><br>Recognition was more related to the thalamic nuclei and caudate nucleus, particularly in the left side.	Brain structures act as a complex functional system
10.1017/S1355617710000998	Brazil	15 AD (74.26)<br>17 aMCI (68.29)<br>16 HC (69.12)	**Imaging**<br>MRI	Boston Naming Test<br><br>MMSE<br><br>RAVLT<br><br>LNI<br><br>FDS<br><br>BDS<br><br>CSDD	VBM	Minimal gradient of temporal lobe atrophy in AD or MCI;	Coordinate and circumlocutory semantic error production on the Boston Naming Test was weakly correlated with anterior temporal lobe (ATL) gray matter density
10.1111/j.1468-1331.2008.02408.x	Brazil	15 AD (74.26)<br>17 aMCI (68.29)<br>16 HC (69.12)	**Imaging**<br>MRI	MMSE<br><br>RAVLT<br><br>LNI<br><br>FDS<br><br>BDS	VBM	aMCI GM atrophy was similarly distributed but less intense than that of AD group, mainly in thalami and parahippocampal gyri;<br><br>No diferences between aMCI and HC in atrophy;<br><br>AD group has WM atrophy in periventricular areas, corpus callosum and WM adjacent to associative cortices.;	GM atrophy is similar between aMCI and AD<br><br>WM atrophy is specific to AD;

10.4103/0028-3886.284376	Mexico	11 AD (74.8)<br>11 mild (74.4)<br>12 HC (70.4)	**Imaging**<br>MRI	neuropsychological evaluation	DTI	Global Radial diffusivity has an overall accuracy was 64.5%, with areas under the curve of 0.81, 0.73 and 0.66 to diagnose AzD, MCI, and healthy brains, respectively.	Global Radial diffusivity has an overall accuracy was 64.5%, with areas under the curve of 0.81, 0.73 and 0.66 to diagnose AzD, MCI, and healthy brains, respectively.
10.3233/JAD-170771	Unable to access	35 bvFTD<br>34 AD<br>29 HC	Imaging<br>MRI	FCSRT	Visual rating<br>VBM	VBM results in bvFTD (pFWE<0.05) showed similar prefrontal and hippocampal regions in addition to striatal and lateral temporal involvement.	43% of bvFTD patients presented with a genuine amnesia.<br><br>Memory recall & storage performances were predicted by atrophy in rostral prefrontal and hippocampal/perihippocampal regions in AD and bvFTD;<br><br>medial/lateral temporal atrophy is associated with memory deficits in bvFTD patients.<br><br>.
10.1093/cercor/bhab421	Latin Americans (RedLat centers)	MRI dataset<br>21 PD (70.47)<br>20 bvFTD (67)<br>18 AD (73.05)<br>33 HC (71.69)<br><br>EEG dataset<br>12 PD (73)<br>15 bvFTD (69.68)<br>15 AD (73.46)<br>21 HC (73.48)	Imaging<br>MRI<br>fMRI<br><br>Other<br>EEG	MOCA<br>IFS<br>UPDRS-II<br>CDR	VBM<br>Connectivity	Volume atrophy and hypoconnectivity in disease associated regions.	PD patients presented action–text deficits related to the volume of action–observation regions, connectivity across motor-related and multimodal-semantic hubs, and frontal hd-EEG hypoconnectivity;<br><br>BvFTD patients exhibited social–text deficits, associated with atrophy and spatial connectivity patterns along social-network hubs, alongside right frontotemporal hd-EEG hypoconnectivity;<br><br>AD patients showed impairments in all stories, widespread atrophy and spatial connectivity patterns, and heightened occipitotemporal hd-EEG connectivity.

10.1016/j.biopsych.2022.02.955	Not described	19 bvFTD (68.5)<br>33 AD (69.8)<br>42 HC (74.6)	Imaging<br>MRI<br><br>Other<br>ECG	Moca<br>IFS<br>Facial emotion recognition	Connectivity<br>Volume	Increased rsHEP modulation in bvFTD was associated with decreased brain volume and connectivity of the AIN.<br><br>Machine learning results confirmed AIN specificity in predicting the bvFTD group.	bvFTD presented more negative rsHEP amplitudes with sources in critical hubs of the AIN (insula, amygdala, somatosensory cortex, hippocampus, anterior cingulate cortex) than AD and HC;<br><br>Exacerbated rsHEP modulation selectively predicted the patients' cognitive profile (including cognitive decline, executive dysfunction, and emotional impairments)
10.1017/s1041610202008281	Brazil	39 AD (73.08)<br>21 MCI (69.52)<br>20 HC (69.15)	Imaging<br>MRI	MMSE<br>CAMCOG<br>CAMCOG memory subscale	Volume	Amygdala, hippocampus, and parahippocampal gyrus were reduced ($p < .005$) in AD patients compared to MCI subjects and controls;<br><br>Discriminant function classified 88.14% of the AD patients and controls, 81.67% of AD patients and MCI subjects, and 80.49% of the MCI and HC	Measures of MTL regions are useful to identify mild to moderate AD patients and MCI subjects, separating them from normal elderly individuals.
10.1089/brain.2021.0154	Brazil	32 MCI-AD<br>25 MCI-SNAP<br>35 HC	Imaging<br>fMRI<br><br>Fluid<br>CSF	Unable to access	Connectivity	The ROI-to-ROI analysis of MCI-AD versus MCI-SNAP showed no differences;<br><br>MCI-AD versus controls showed decreased FC between ROIs of the SN and between ROIs from SN and VN;<br><br>MCI-SNAP versus controls showed increased FC between an ROI of DMN and VN.	no differences in neuropsychological data between the MCI groups;<br><br>SN, DMN, and VN are multimodal networks with high value/high cost and may be more vulnerable to AD pathogenic processes;
10.1016/s0197-4580(02)00084-2	Brazil	14 AD (72.2)<br>14 HC (69.4)	**Imaging**<br>MRI	CAMDEX<br>MMSE<br>MADRS<br>CAMCOG<br>FOME delayed recall<br>Word list recall	VBM<br>Volume	Reduced GM volume in AD patients were detected in medial and lateral temporal regions, most significantly in the right and left posterior parahippocampal gyri and the left posterior inferior temporal gyrus/fusiform gyrus.	VBM results confirm previous findings of temporal lobe atrophic changes in AD,

10.1136/jnnp.2003.019273	Brazil	27 Very mild AD (68.2)<br>39 Mild AD (72.8)<br>Moderate to Severe AD (72.7)<br>19 HC (67.7)	**Imaging**<br>MRI	CDR<br>Token<br>BNT<br>COWAT<br>MMSE<br>UPDRS	Volume<br>WMH	Mild and moderate to severe AD patients had significantly more WMH than controls (p>0.05);<br><br>WMH preferentially involved the frontal lobes , were inversely correlated with grey matter cortical volume	WMH were significantly associated with vascular risk factors and with a worse performance on memory tasks.
10.1016/j.pscychresns.2006.04.003	Brazil	14 AD (72.2)<br>14 HC (69.4)	**Imaging**<br>MRI	CAMDEX<br>MMSE<br>MADRS<br>CAMCOG<br>FOME delayed recall	VBM<br>Volume	Corpus callosum (CC) atrophy was detected in the antero-superior portion of the splenium, the isthmus, the anterior and posterior portions of the CC body, and the rostral portion of the genu.	Clusters of positive correlation with MMSE scores were seen on the left anterior CC body.
10.1186/1742-2094-7-6	Brazil	54 AD<br>66 HC	**Imaging**<br>MRI<br><br>CSF<br>Serum NSE and	MMSE<br>CDR	Volume	NSE levels decreased in AD patients with higher levels of brain atrophy.	Serum S100B levels were lower in AD group;<br><br>S100B levels were positively correlated with CDR scores and MMSE;
10.1523/jneurosci.1312-22.2022	Chile<br>Argentina	42 AD (76.85)<br>65 HC (69.9)	**Imaging**<br>MRI<br><br>**Other**<br>EEG	Moca	resting-state high density EEG (hdEEG)<br><br>Connectivity<br><br>Volume	Local level: temporoparietal and frontal regions were affected by AD;<br><br>Network level: The limbic, frontoparietal, default mode, and salience networks were affected by AD;<br><br>The temporal reversibility was associated with cognitive decline in AD and gray matter volume in HCs.	AD was associated with a breakdown of temporal irreversibility at the global, local, and network levels, and at multiple oscillatory frequency bands.
10.1007/s11682-018-0002-2	Brazil	15 AD (74.5)<br>15 MCI (74.3)<br>15 HC (74.6)	**Imaging**<br>MRI	-	DTI	-	this multilevel approach achieved an average classification accuracy of 90% between AD and HC, 83% between AD and MCI, and 83% between MCI and HC.

10.3174/ajnr.A2232	Brazil	16 AD<br>17 MCI<br>16 HC	**Imaging**<br>MRI	CDR	Texture Analysis (TA)<br>DTI	TA on CC and thalamus diffrenetiated groups.	A pair-wise comparison among groups showed differences for: 1) AD-control aMCI-AD for the CC; 2) AD-control, aMCI-AD, and aMCI-control for the thalamus.
10.1016/j.jpsychires.2020.01.018	Argentina	31 offspring of patients with late-onset AD (O-LOAD) (50.36)<br>28 HC (53.90)	**Imaging**<br>MRI<br>PET	RAVLT<br>MMSE<br>CDR<br>TMT-A/B<br>TOL<br>BNT<br>WAIS-IV	Functional connectivity<br>Volume<br>Amyloid load	O-LOAD individuals showed decreased global connectivity in a cluster encompassing the left LC;<br><br>Seed-to-voxel analyses revealed this finding was largely explained by decreased connectivity between the LC and the cerebellar cortex;	FC between the LC and the left cerebellum correlated positively with delayed recall scores;<br><br>FC between the LC and the cerebellar cortex is decreased in the healthy offspring of patients with LOAD, such connectivity measurements being associated with delayed memory scores.
10.1038/s41598-019-50599-x	Latin America (argentina or colombia?)	18 Fronto-insular stroke (FIS - 62)<br><br>21 bvFTD (69.8)<br><br>16 AD patients (74.19)<br><br>20 HC (68.05)	**Imaging**<br>MRI<br>SPECT	Visual perception task (confidence and wagering)	Volume<br>CBF	bvFTD presented frontotemporo-insular atrophy on MRI and frontal hypoperfusion in SPECT;<br><br>Lesion (VBM) in FIS and atrophy of bvFTD and AD patients overlad wehn compared to HC;	For bvFTD, the GM volume from fronto-temporo-insular areas was negatively correlated with wagering;<br><br>For AD, GM volumes from ventromedial and fronto-temporo-insular regions were significantly correlated with the confidence and wagering indexes;<br><br>Ventromedial compromise was related to overconfidence, whereas fronto-temporo-insular damage was associated with excessive wagering.

10.1136/jnnp.73.5.508	Brazil	9 AD (70)<br>10 HC (70.1)	Imaging<br>MRI<br>Spect	CAMCOG<br>Category fluency<br>FOME delayed recall	Volume<br>CBF	Measures of hippocampal atrophy were negatively correlated with rCBF values in voxel clusters located in the frontal lobes, involving the right and left inferior frontal gyri and the insula	Direct correlations were detected between the hippocampal grey matter density and rCBF values in voxel clusters located bilaterally in the temporal neocortex, in the left medial temporal region, and in the left posterior cingulate cortex during the memory task in the Alzheimer's disease group (p < 0.001).

Supplementary Table 4: Genetic Studies in Latin America							
**Study**	**Regions/ Country/ Population**	**Sample disease (mean age)**	**Biomarker** (type, markers)	**Cognitive measures**	**Brain measures**	**Biomarker results**	**Multimodal results**
Ruiz- Sánchez et al (2017)	Latam/ Mexico/ Mexican	454 HC (62.63) 227 PD (62.01)	NR42A polymorphisms(rs34884856, rs35479735) PBMC (NR4A2 mRNA)	-	-	HC < PD in genotype and allelic frequency of alelle 3G rs35479735 Recessive model of the rs35479735 polymorphism was associated with an increased risk of PD (OR=1.53) mRNA levels of NR4A2 in PD were significantly lower than for HC	
Romero-Gutiérrez et al (2021)	Latam/ Mexico/ Mexican Mestizo	193 HC (69.8) 118 PD (69.92)	LRRK2 (rs1491942) MTHFR (rs1801133) USP24 (rs13312) PARK7 (rs3766606) NUCKS1 (rs823128) SLC41A1 (rs823156) GSK3B (rs334558) DRD3 (rs6280) FAM47E/ SCARB2 (rs6812193) SNCA (rs356219) PARK2 (rs1801474) PARK2 (rs1801582) ANKK1 (rs1800497) LRRK2 (rs1994090) MAPT (rs242562) RAIL/ SREBF1 (rs11868035)	MMSE		Stratification by ancestry: a risk effect for MTHFR rs1801133 was observed only in the group with the highest percentage of European ancestry. PD risk effect for LRRK2 rs1491942 was significant in subjects with a higher ratio of Native American ancestry.	

Torrealba-Acosta et al (2021)	Latam/<br>Costa Rica/<br>Costa Rican	97 HC<br>118 PD (62.12)	GBA, SNCA, VPS35, LRRK2, GCH1, PRKN, PINK1, DJ-1, VPS13C, ATP13A2	MoCA		One pathogenic GCH1 variant, p.K224R<br>Rare variants in LRRK2 were nominally associated with PD; six were located between amino acids p.1620 and 1623 in the C-terminal-of-ROC (COR) domain of Lrrk2	
Tipton et al (2020)	Latam/<br>Colombia/<br>Colombian	58 HC (58.9)<br>142 PD (62.7)	GBA (p.K198E)			Three carriers (2.1%) of the GBA p.K198E substitution in the Colombian PD cohort, and one carrier (1.7%) in the Colombian control cohort<br>p.K198E was not significantly associated with risk of PD in Colombians	
Tipton et al (2020)	Latam/<br>Colombia and Ecuador/<br>Colombian and Ecuadorian	26 HC Colombia (56)<br>55 PD Colombia 26 EOPD (53), 29 LOPD (67)<br>426 PD Ecuador 71 EOPD (55), 355 LOPD (73)	LRRK2 (p.G2019S, p.R1441G)<br>VPS35 (p.D620 N)<br>MAPT (p.A152T)<br>GBA (p.E326K, p.N370S)<br>APOE (p.C130R, p.R176C)<br>PARKIN, PINK1, SNCA, DJ1			In Ecuador, pathogenic mutations in MAPT (p.A152T) were identified. GBA genotyping identified p.E326K in three PD cases and p.N370S in one PD case; APOE genotyping identified five ε4ε4 carriers<br>In Colombia, pathological variants were identified in PARKIN, PINK1, LRRK2, GBA, and MAPT. APOE genotyping identified two ε4ε4 carriers	
Miranda-Morales et al (2019)	Latam/<br>Mexico/<br>Mexican Mestizo	108 HC<br>108 PD<br>(age 70.1)	H1/H2 MAPT haplotype			No statistically significant differences were observed between groups in both, allelic and genotypic frequencies.	
Velez- Pardo et al (2019)	Latam/<br>Colombia and Peru/<br>Colombian and Peruvian	Colombia<br>164 HC (53.8)<br>131 PD (64.6)<br>Peru<br>155 HC (54)<br>471 PD (62.1)	GBA			Thirty-eight participants carried pathogenic mutations<br>The frequency of GBA pathogenic mutation carriers in the Colombian PD cohort (9.9%) was more than double that observed among Peruvian PD patients (4.2%)	
Amaral et al (2019)	Latam/<br>Brazil/	81 HC (67.3)<br>81 PD (69.5)	GBA			7.4% of the PD patients presented one of the two most common mutations of the GBA gene, p.N370S and p.L444P	

	Brazilian						
Voigt et al (2019)	Latam/<br>Brazil/<br>Brazilian	122 PD (60.5)	CHCHD2			No pathogenic or risk variants in the CHCHD2 gene in Brazilian families with PD	
Cornejo- Olivas et al (2017)	Latam/<br>Argentina, Brazil, Colombia, Peru, Uruguay, Ecuador	Argentina<br>188 PD (60.5)<br>Brazil<br>352 HC (58.8)<br>433 PD (63.5)<br>Colombia<br>184 HC (53.1)<br>197 PD (66.2)<br>Peru<br>255 HC (58.2)<br>543 PD (62.3)<br>Uruguay<br>306 HC (60.9)<br>288 PD (63.3)<br>Ecuador<br>85 PD (67.6)	LRRK2			29 Parkinson's disease patients who carried p.G2019S and the frequency ranged from 0.2% in Peru to 4.2% in Uruguay<br>Only two Parkinson's disease patients carried p.R1441G and one patient carried p.R1441C<br>The frequency of LRRK2-p.G2019S varied greatly between different Latin American countries and was directly correlated with the amount of European ancestry observed	
Abreu et al (2016)	Latam/<br>Brazil/<br>Brazilian	141 familial PD (60.7)	LRRK2<br>SNCA<br>VPS35<br>GBA			Heterozygous mutations in 7.0% PD<br>The LRRK2 G2019S mutation was found in 4.2% and GBA mutations, L444P substitution in 2.1% and N370S substitution in 0.7%<br>No mutations were found in SNCA or VPS35 genes	
Longo et al (2015)	Latam/<br>Brazil/	140 HC (51.0)<br>154 PD (67.9)	SNCA (p.A53T)			SNCA p.A53T mutation was not identified in PD patients.	

	Brazilian						
Pimentel et al (2015)	Latam/ Brazil/ Brazilian	592 PD (61.1)	SNCA			p.E46K mutation in a 60-year-old man, born in Bolivia, with a familial history of autosomal dominant PD	
Spitz et al (2015)	Latam/ Brazil/ Brazilian	8 PD (1 family)	LRRK2 GBA	MMSE		6 subjects analyzed were found to have both LRRK2 G2019S and GBA L444P mutations, only one has developed clinical parkinsonism, reinforcing the variable penetrance of each gene	
Duque et al (2015)	Latam/ Colombia/ Colombian	162 HC (74.9) 154 PD 24 familial (48.7) 130 sporadic (53.4)	LRRK2 (p.G2019S)			The frequency of LRRK2 p.G2019S mutation was 1.3% in patients and 0.6% in controls The p.G2019S mutation is not an important causal factor of Parkinson Disease in Colombia	
Chien et al (2014)	Latam/ Brazil/ Brazilian	100 HC (71.2) 100 PD (73.3)	LRRK2 gene (p.G2019S)			No G2019S mutations were found in both patients with sporadic PD and controls.	
García et al (2014)	Latam/ Mexico/ Mexican Mestizo	208 HC (68.3) 173 PD (62.8)	LRRK2 (Gly2019Ser, p.Gly2385Arg) Gross gene deletions/duplications in 6 PARK genes: PARKIN (PARK2), PINK1 (PARK6), DJ-1 (PARK7), LRRK2 (PARK8), SNCA (PARK1/4) and ATP13A2 (PARK9)			LRRK2 p.G2019S was found in 2 PD patients. Heterozygous deletion of exon 2 in PARK2 (PARKIN) was found in 1 PD patient	

Monroy-Jaramillo et al (2014)	Latam/<br>Mexico/<br>Mexican Mestizo	120 HC (45-82)<br>127 EOPD (AOO 34.9)	PARK2, PINK1, and DJ-1			The mutation frequencies were 20.5% in PARK2, 9.0% in PINK1, and 0.8% in DJ-1<br><br>The presence of two mutations in compound heterozygous or homozygous genotypes was found in 16 unrelated patients, 10 had mutations in PARK2, six in PINK1, and none in DJ-1.<br><br>Two PARK2-PINK1 and one PARK2- LRRK2 digenic cases were observed.<br><br>Seventeen different copy number variants (CNVs) were observed in PARK2, including deletions and one compound heterozygous harboring exon 12 duplication.<br><br>In PINK1, five different CNVs and two-point mutations were observed<br><br>Novel mutations were identified in PARK2 and PINK1 genes, including PINK1 duplication for the first time.	
Gatto et al (2014)	Latam/<br>Argentina/<br>Argentinian	55 PD (68.8)<br>16 familial<br>9 Ashkenazi- Jewish ancestry<br>7 Basque ancestry	LRRK2 (p.G2019S)	MMSE<br>ToM		Heterozygous LRRK2 G2019S mutation was found in 3 PD unrelated<br><br>It represents the 18.75% of familial cases, 33.33% among patients with Ashkenazi- Jewish ancestry and the 5.45% of the overall analyzed population<br><br>The prevalence of LRRK2 G2019S mutation in this Argentinean cohort was similar to other international series, with a higher prevalence in Ashkenazi Jewish.	Executive functions and Theory of Mind (ToM) abilities were evaluated exclusively in LRRK2 G2019S mutation carriers: MMSE was abnormal in one patient (MMSE < 24/30). In the other two patients, the extensive neuropsychological battery showed ToM impairment.
Moura et al (2013)	Latam/<br>Brazil/<br>Brazilian	136 PD (49.8)	PARK2<br>PINK1			Six missense variants (2.9%) in PARK2 gene were found. No pathogenic mutation was identified in PINK1 gene.<br><br>All putative pathogenic variants found were in heterozygous state.	

Kumar et al (2012)	Latam/<br>Chile/<br>Chilean	223 PD (67.3)<br>192 family PD	VPS35 (p.D620N)	MMSE<br>MoCA		No mutation was identified in Chilean patients	
Guimarães et al (2012)	Latam/<br>Brazil/<br>Brazilian	186 HC (60.7)<br>347 PD (60.7)<br>60 family PD	GBA (p.N370S, p.L444P)			p.N370S and p.L444P mutations in GBA was found in 3.7% of PD	
Moura et al (2012)	Latam/<br>Brazil/<br>Brazilian	102 PD (51.9)<br>24 family PD<br>78 sporadic PD	Gene dosage PARKIN, SNCA, DJ-1 and PINK1			3.9% with copy number variations of specific exons in genes PARKIN and PINK1<br>No SNCA or DJ-1 dosage alterations were observed	
Guerrero et al (2012)	Latam/<br>Mexico/<br>Mexican Mestizo	120 HC (45-82)<br>63 EOPD (AOO 16-45)	Gene dosage PARK2			Exon rearrangements were present in 32 patients, with 17.5% carrying simple heterozygous and 25.4% carrying compound heterozygous PARK2 mutations.	
Dávila-Ortiz et al (2011)	Latam/<br>Mexico/<br>Mexican Mestizo	205 HC (24.7)<br>127 PD (59)	H1/H2 MAPT haplotype			In control, 88.78% H1 and 11.22% H2 were found. Haplotypes were found in 80.49% H1/H1, 16.59% H1/H2 and 2.93% H2/H2<br>InPD patients, 91.73% H1 and 8.27% H2 were found. Haplotypes were found in 83.46% H1/H1, 16.54% H1/H2 and 0% H2/H2<br>Increased frequency of H1 alleles, as well as H1/H1 genotype, and absence of H2/H2 genotype in patients with PD	

Mata et al (2011)	Latam/<br><br>Peru, Chile, Uruguay, Argentina	Peru<br><br>148 HC (53.5)<br><br>492 PD (62.4)<br><br>Chile<br><br>162 HC (67.6)<br><br>358 PD (71.8)<br><br>Uruguay<br><br>205 PD (63.1)<br><br>Argentina<br><br>95 PD (62.2)	LRRK2 (p.Q1111H)			p.Q1111H in LRRK2 gene was found in 10.3% Peruvian PD, 4.6% Chilean PD, 1% Uruguay PD and 1.1% Argentinian PD	
Ramirez et al (2011)	Latam/<br><br>Chile/<br><br>Chilean	195 HC (66)<br><br>169 PD (68)	PARK16			A lower minor allele frequency (MAF) in the Chilean PD patients (9.3%) than in controls (16.5%)<br><br>PARK16 showed no association with PD in the Chilean sample.	
Dos Santos et al (2010)	Latam/<br><br>Brazil/<br><br>Brazilian	110 PD (52.6)	GIGYF2<br><br>ATP13A2<br><br>GBA			No clearly pathogenic mutations were identified in ATP13A2 and GIGYF2.<br><br>A higher frequency of known pathogenic mutations in GBA gene was identified in PD cases (5.4%)	
Abdalla-Carvalho et al (2010)	Latam/		LRRK2			Four polymorphisms, a novel silent variant p.R1398R and four substitutions: p.T1410M, p.G2019S, p.Y2189C and the novel variant p.C2139S were identified	

	Brazil/<br>Brazilian	210 HC (65.4)<br>204 PD (62.0)				The most prevalent mutation was the p.G2019S (2.4%)	
Yescas et al (2010)	Latam/<br>Mexico/<br>Mexican	319 PD (AOO 52.4)	LRRK2			0.94% patients, two with sporadic PD and one with familial PD, were heterozygous for LRRK2 mutations.	
Marder et al (2010)	Latam/<br>Dominican Republic, Puerto Rico, Mexico, Cuba, Peru, Colombia, Chile, and Ecuador	956 PD (52.4)<br>77 PD from Latam	PARKIN (PARK2)			6.7% PD had PARKIN mutations (3.9% heterozygous, 0.6% homozygous, and 2.2% compound heterozygous)<br>Deletions in exons 3 and 4 and 255delA were common among Hispanics.<br>Hispanic race/ethnicity was associated with carrying a heterozygous mutation<br>The 12 Hispanic PARKIN carriers included 7 Puerto Ricans, 2 Mexicans, 1 Cuban, 1 Dominican, and 1 Peruvian.<br>Six patients (5 Puerto Rican and 1 Mexican) carried deletions in exons 3 and 4. Both homozygous carriers with deletions in exons 3 and 4 were of Puerto Rican descent	Compared with white non-Hispanic race/ethnicity, Hispanic race/ethnicity was associated with carrying a parkin mutation in the model examining the presence of any parkin mutation (OR= 2.7) and in the model examining heterozygous carriers compared with noncarriers (OR=2.8)
Mata et al (2009)	Latam/<br>Peru, Uruguay	Peru<br>240 PD (64.2)<br>Uruguay<br>125 PD (63.4)	LRRK2 (p.R1441C, p.R1441G, p.G2019S)			Seven patients identified with mutations, one with p.R1441G, and six with p.G2019S.<br>The carrier frequency was significantly greater in the Uruguayan cohort (4.8%) than in the Peruvian cohort (0.4%)	
Socal et al (2009)	Latam/<br>Brazil/<br>Brazilian	27 PD (51.55)	SCA2<br>SCA3	MMSE<br>ADL		SCA2 and SCA3 mutations were detected in 13% of patients.	
Camargos et al (2009)	Latam/<br>Brazil/<br>Brazilian	226 PD<br>EOPD (AOO 34.8)<br>LOPD (AOO 52.3).	LRRK2 and PINK1			Seven PD patients with mutation, 5 had mutation in PKRN gene, 1 patient had a novel LRRK2 variant, p.Q923H and identification of a novel mutation in PINK1 (homozygous deletion of exon 7).	

Pineda–Trujillo et al (2009)	Latam/<br>Colombia/<br>Colombian	4 PD patients from 1 family (AOO 14-25)	LRRK2			mutation c.255delA, at exon 2 of PARK2	
Pimentel et al (2008)	Latam/<br>Brazil/<br>Brazilian	154 PD (62.6)	LRRK2			The LRRK2 p.G2019S mutation was present in heterozygous state in three index cases (~2%), and in three additional relatives.	
Aguiar et al (2008)	Latam/<br>Brazil/<br>Brazilian	81 HC<br>72 PD (45.65)	PARK2 and PARK8			A novel PARK2 mutation (p.D53X) was identified in 2 patients	
Spitz et al (2008)	Latam/<br>Brazil/<br>Brazilian	267 HC (30-76)<br>65 PD (30-76)	GBA			GBA mutations were detected at a significantly higher frequency among PD patients (3%), when compared to the control group (0%).<br>1 patient with L444P mutation and the other with p.L444P+p.E326K mutations.	
López et al (2007)	Latam/<br>Mexico/<br>Mexican	229 HC (63.97)<br>229 PD (62.28)	APOE			APOE-4 allele and APOE 4/3 genotype are associated with PD	
Perez-Pastene et al (2007)	Latam/<br>Chile/<br>Amerindian	611 HC (62)<br>349 PD (66.84)	GST M1		MRI or CT	A significant association of the null mutation in GST M1 (GSTM1*0/0) with PD was found. The association was strongest in the earlier age range.	
Perez-Pastene et al (2007)	Latam/<br>Chile/<br>Chilean	153 HC (61)<br>137 sporadic and 29 familial PD (69)	LRRK2<br>(PARK8)		MRI or CT	LRRK2 p.G2019S was detected in one familial and four sporadic PD patients	
Cintra et al (2018)	Latam/<br>Brazil/<br>Brazilian	63 HC<br>443 ALS<br>67 FTD	C9orf72			Repeat expansion in 1 (7.1%) of the 14 pure familial FTD cases was detected.<br>None of the 11 pure sporadic FTD cases in our cohort carried the expansion.<br>Among the 35 ALS carriers of the C9orf72 mutation, 25.7% had concomitant FTD and among the 15 FTD mutation carriers, 20% had ALS.	

						The highest mutation frequencies were detected in the 39 patients with ALS and FTD overlapping disorders, reaching 50% of familial and 17.6% of sporadic cases.	
Fernández et al (2016)	Latam/ Argentina/ Argentinian	1 bvFTD (51)	C9orf72		MRI SPECT	The index case presented one allele with two replicates G4C2 and another allele with pathological expansion	Brain magnetic resonance imaging (MRI) was performed; diffuse brain and cerebellar atrophy was detected. Brain Tc99 SPECT showed hypoperfusion in anterobasal, polar, and lateral temporal lobe in the left hemisphere; in the right hemisphere, a frontal-orbitary hypoperfusion was observed.
Gatto et al (2017)	Latam/ Argentina/ Argentinian	9 bvFTD and CBS affected from 1 family	MAPT	MMSE<br>Digit span<br>Memory logical<br>RAVLT<br>RCFO<br>BNT<br>SVF<br>PVF<br>TMT A and B		Mutation p.P301L in MAPT gene	An initial cognitive assessment in index patient, performed at age 56, showed some impairment in attention and executive functions.The other cognitive domains were normal. At age 59 the index patient showed marked cognitive impairment involving memory, language, attention, and executive domains.<br><br>Brain MRI showed bilateral and symmetric putaminal hyperintense T1 signals. FDG PET: diffuse left hemisphere, thalamic, mesencephalic, and basal ganglia hypometabolism as well as left motor cortex. 18F-DOPA PET scan left striatal dopaminergic degeneration.
Itzcovich et al (2016)	Latam/ Argentina/ Argentinian	33 FTD (59)<br>50 ALS (61)	C9orf72			The most common alleles were 2, 5, and 8 repeats<br>18.2% of FTD had G4C2 expansion in C9orf72.<br>A G4C2 expansion in C9orf72 explained 37.5% of familial cases	
Miranda et al (2017)	Latam/ Chile/ Chilean	1 FTD (77)	C9orf72			A G4C2 expansion in C9orf72 was found	

Riudavets et al (2013)	Latam/ Argentina/ Argentinian	1 FTD (50)	PSEN1	MMSE<br>Clock drawing<br>TMT A and B<br>BNT<br>Signoret memory<br>Digit span		Mutation p.M146V in PSEN1 gene	
**Study**	**Regions/ Country/ population**	**Sample disease (mean age)**	**Biomarker (type, markers)**	**Cognitive measures**	**Brain measures**	**Biomarker results**	**Multimodal results**
(Abdala, Dos Santos et al. 2017)	Latam/ Brazil/ Brazilian	120 sAD (77)<br>149 HC (71)	**Genetic**<br>PSEN1<br>APOe	-	-	Risk association for rs17125721 in familial AD cases (OR=6.0; IC95%=1.06-33.79; p=0.042).	Risk association for rs17125721 in familial AD cases
(Arango, Cruts et al. 2001)	Latam/ Colombia/ Colombian	11 ADAD (58.3)<br>23 fAD (69.4)<br>42 sAD (70.1	**Genetic**<br>PSEN1 mutations (I143T; E280A; V94M; E318G); PSEN2 (P129).	-	-	No APP mutations; PSEN1 mutations (I143T; E280A; V94M; E318G); PSEN2 (P129).	Most of the Colombian AD cases, predominantly late-onset, were negative for PSEN and APP mutations
(Arboleda-Bustos, Ortega-Rojas et al. 2018)	Latam/ Colombia/ Colombian	358 LOAD (68.80)<br>329 HC (67.93)	Genetic<br>APOE<br>TREM2<br>The p.Q33* (rs104894002), p.R47H (rs75932628), p. R62H (rs143332484), and p.D87N (rs142232675)	-	-	The minor allele frequency of rs75932628-T was 0.009 in AD and was not found in any HC, which suggests a significant association between rs75932628-T and LOAD risk in our sample (P=0.010).<br>The rs143332484-T variant did not exhibit a significant association (P=0.160), whereas rs104894002 and rs142232675 were not found.	The rs75932628-T variant of TREM2 is an important risk factor for LOAD in the Colombian population.
(Bahia, Kok et al. 2008)	Latam/ Brazill/ Brazilian	120 AD (75.2)<br>120 HC (71.1)	**Genetic**<br>APOe<br>APOE-491<br>APOE-219<br>LRP	-	-	AD > e4 alleles;<br>2 e4 alleles higher risk for AD;<br>1 e2 allele protective.<br>T allele of the -219 polymorphism associated with increased risk of AD.	E4 allele and T -219 polymorphism were associated with an increased risk for AD
(Barral, Cheng et al. 2015)	Caribean/ Dominican Republic and Puerto Rico/ Caribbean Hispani	Late Onset families<br>**67 families**<br>354 Affected (68)<br>114 Unaffected (68)<br>**215 additional families**<br>434 Affected (68)<br>156 Unaffected (68)	**Genetics**<br>MS4A	-	-	26 regions linked to LOAD. The strongest signal was at 11q12.3 (rs2232932: w2 Mb upstream of the membrane-spanning 4A gene cluster. We additionally identified a locus at 7p14.3 (rs10255835), a region harboring genes associated with the nervous system (GARS, GHRHR, and NEUROD6).	These regions may harbor familial LOAD causative mutations.

(Belcavello, Camporez et al. 2015)	Latam/ Brazil/ Brazilian	82 LO AD (81.16) 161 HC (79.36)	**Genetic** MTHFR PICALM CLU CR1	-	-	MTHFR (rs1801133) CT > AD; PICALM (rs3851179) GG > AD; e4 carriers > AD	Significant association of the MTHFR rs1801133 and PICALM rs3851179 with AD
(Benedet, Moraes et al. 2012)	Latam/ Brazil/ Brazilian	120 LOAD (72.3) 412 HC (70.8)	**Genetic** 12 ancestry informative markers - European and Native American frequencies (rs730570, rs3796384, rs1426654, rs734780, rs1129038, rs2278354, rs2065160, rs4305737), five between European and African frequencies (rs803733, rs1426654, rs2814778, rs1129038, rs1240709), and two between African and Native American frequencies (rs2814778, rs3138521)	-	-	3-fold greater genetic Amerindian content among control subjects compared to AD patients APOe4 carriers > AD;	Allelic architecture of Native Americans can confer protection against the onset of the disease
(Bicalho, Pimenta et al. 2013)	Latam/ Brazil/ Brazilians	169 sAD (78.9) 97 HC (78.1)	**Genetic** ApoE BDNF COMT, 5-HTTLPR; genomic ancestry	-	-	ApoE rs429358 > AD ApoE e3 > HC ApoE e4 > AD	ApoE was the only polymorphism associated with AD
(Blue, Horimoto et al. 2019)	Caribbean/ Multiple countries/ Caribbean Hispanics	LOAD (73.28) HC (74.84)	**Genetic** APOe	-	-	Caribbean Hispanics individuals with African-derived ancestry at APOE had 39% lower odds of AD than individuals with European-derived APOE; APOE E2 and E4 effects on AD risk and age at onset were significant in the Caribbean Hispanics	Additional genetic variation in the APOE region influences AD risk beyond APOE E2/E3/E4.
(Braga, Silva et al. 2015)	Latam/ Brazil/ Brazilian	MMSE < 20 CHEIs Responders 25 AD (82.9) Non-responders	**Genetic** APOE CHRNA7	MMSE	-	Analysis of CHRNA7 rs6494223 showed that 30.9% of patients were C/C wild type, 52.1% C/T heterozygotes (n ¼ 86), and 17.0% T/T homozygotes (n ¼ 28). No differences were found between the observed frequencies and the expected	CHRNA7 T allele and a better response to treatment with ChEIs in patients with mild AD (MMSE 20)

		60 AD (80.26)<br>MMSE > 20<br>Responders 39 AD (79.79)<br>Non-responders<br>53 AD (79.88)				HWE frequencies. The analysis of the APOE polymorphisms showed that 2.4% had the APOE e2/3, 1.2% had the e2/4, 43.6% had the e3/3, 45.5% had the e3/4, and 7.3% had the e4/4 genotypes	e4 allele worse response to CHEIs;<br>SNP rs6494223 of CHRNA7 better response to CHEIs
(Camelo, Arboleda et al. 2004)	Latam/<br>Colombia/<br>Colombian	83 AD (69)<br>69 (71.4)	**Genetic**<br>APOe4<br>ACE<br>A2M	-	-	No association between the I/D polymorphisms of ACE and A2M with AD	APOe4 as a risk factor
(Campos, Edland et al. 2013)	Mexico/<br>Mexican	-	**Genetic**<br>APOe4	-	-	APOE-e4 allele frequency was lower in Mexican Hispanic and not associated to AD	APOE-e4 allele offers lower risk in Mexican Hispanic
(Cardoso, Ong et al. 2012)	Latam/<br>Brazil/<br>Brazilian	28 AD (80.6)<br>29 HC (71.2)	**Genetic**<br>GPx1 Pro198Leu	-	-	No differences between AD and HC	No differences between AD and HC
(Cordeiro, Noguti et al. 2010)	Latam/<br>Brazil/<br>Brazilian	58 AD (73.56)<br>107 HC (72.18)	**Genetic**<br>PLA2 enzymes	-	-	Genotypic association between the BanI cPLA2 polymorphism and LOAD ($\chi 2=6.25$, 2df, $p=0.04$), however there was no allelic association.	Genotypic association between the BanI cPLA2 polymorphism and LOAD.
(da Silva, Ramos et al. 2006)	Latam/<br>Brazil/<br>Brazilian	42 AD (73.8)<br>50 HC (73.9)	**Genetic**<br>C677T<br>MTHFR	-	-	No differences in the C677T and A1298C MTHFR polymorphisms	No differences in the C677T and A1298C MTHFR polymorphisms
(de Bem, Pezzi et al. 2016)	Latam/<br>Brazil/<br>Brazilian	104 AD (76.67)<br>108 HC (74.96)	**Genetic**<br>APOE<br>DNMT3B	MMSE	-	Interaction of DNMT3B gene with the APOEe4 in this sample of AD patients	DNMT3B gene interacts with the APOEe4
(De Luca, Spalletta et al. 2019)	Latam/<br>Brazil/<br>Brazilian	135 AD (79)	**Genetic**<br>APOE	-	-	APOE e4 showed a anticipatory effect specific for LOAD	APOE e4 anticipatory effect for LOAD in the Brazilian Sample.
(de Mendonca, Salazar Alcala et al. 2016)	Latam/<br>Venezula/<br>Venezuelan	79 AD (70)<br>100 HC (71)	**Genetic**<br>APOE<br>GSTT1	-	-	Ala/Ala genotype increases the risk provided by the APOE e4 allele AlaAla/ε3ε4 (OR = 3.47, P = .03), AlaAla/ε4ε4 (OR = 6.3, P = .01)	Presence of the Ala/Ala genotype increases the risk provided by the 4 allele of the APOE gene: AlaAla/ε3ε4.
(de-Almada, de-Almeida et al. 2012)	Latam/<br>Brazil/<br>Brazilian	82 AD (82.2)<br>182 HC (78.3)	**Genetic**<br>APOE	MMSE<br>CDR	-	ApoE3 allele protective ((OR = 0.46, 95%CI = 0.30-0.67; P < 0.0001)	ApoE3 allele - protective

(de-Andrade, Larrandaburu et al. 2000)	Latam/ Brazil/ Brazilian (Afro-Brazilians)	23 AD<br>343 HC-Causasian (57)<br>100 HC-Afro-Brazilian (35)	**Genetic**<br>APOE	-	-	Association between the APOE4 allele and AD. APOE*4 frequency was higher in AD.	Association between the APOE4 allele and AD;<br>APOEe4 frequency was higher in AD.
(Dumois-Petersen, Gallegos-Arreola et al. 2020)	Latam/ Brazil/ Brazilian	79 AD (81.6)<br>145 HC (80.1)	**Genetic**<br>CD33<br>ABCA7<br>CR1<br>MS4A6A	MMSE	-	No association between AD and rs3764650 ABCA7, rs6656401 CR1, and rs610932 MS4A6A was observed; rs3865444 CD33 exhibited a significant association with AD, whereas the GG genotype exhibited a 1.938-fold risk (1.084–3.465; p = 0.027) of developing AD, and the GT genotype exhibited a 0.530-fold risk (95% CI 0.299–0.942; p = 0.042) for AD protection.	rs3865444 CD33 acts as a protective factor against LOAD
(Dumois-Petersen, Gallegos-Arreola et al. 2020)	Latam/ Mexico/ Mexican	39 EOAD (42.5)	**Genetic**<br>PSEN1	MMSE	-	High prevalence of the A431E mutation PSEN1	High prevalence of the A431E mutation PSEN1
(Forero, Arboleda et al. 2006)	Latam/ Colombia/ Colombian	106 AD (73.3)<br>97 HC (72.2)	**Genetic**<br>APOE<br>LRP1<br>MAPT<br>5-HTT	MMSE	-	No association for LRP1, Tau and 5-HTT polymorphisms in male, female or APOE4 patients and in patients with or without family history of AD	No association for LRP1, Tau and 5-HTT polymorphisms
(Forero, Benitez et al. 2006)	Latam/ Colombia/ Colombian	102 AD (73.3)<br>168 HC (68.8)	**Genetic**<br>BDNF<br>COMT<br>UCHL1	-	-	No associations among AD and polymorphisms in BDNFVal66Met; COMTVal158; UCHL1S18Y	No associations among AD and polymorphisms in BDNFVal66Met; COMTVal158; UCHL1S18Y
(Fox-Fuller, Martinez et al. 2023)	Latam/ Colombia/ Colombian	35 ADAD (30.4)<br>26 HC<br>(47.6)	**Genetic**<br>PSEN1 E280A	Memory for Semantically Related Objects test (MESERO)<br>MMSE	-	CU ADAD had significantly worse MESERO performance	CU ADAD had significantly worse MESERO performance
(Fullerton, Clark et al. 2000)	Latam/ Mexico/ Mexican-Mayans	(?) 48 (?)	**Genetic**<br>APOE	-	-	8 polymorphic sites (560, 832, 1163, 1998, 2440, 3937, 4075, and 5229B)	8 polymorphic sites (560, 832, 1163, 1998, 2440, 3937, 4075, and 5229B)
(Granot-Hershkovitz,	LAC/ Multiple/ Multiple	875 Cuban (62.6)<br>424 Dominican (61.6)	**Genetic**<br>APOE	-	-	APOEε4 was associated with an increased risk of significant cognitive decline (odds ratio [OR] = 1.15, P-value = 0.03), with the strongest association in Cubans. APOEε2 was associated	APOEε4 offers increased risk for cognitive decline and APOEε2

Tarraf et al. 2021)		1411 Mexican (61.7)<br>734 Puerto Rican (62.9)<br>313 South American (61.96)<br>417 Central American (61.3)				with decreased risk of MCI (OR = 0.37, P-value = 0.04) in Puerto Ricans.	was associated with decreased risk of MCI.
(Itzcovich, Chrem-Mendez et al. 2020)	Latam/<br>Argetina/<br>Argentinian	2 mutation carriers (49 and 54)	**Genetic**<br>PSEN1<br>**Imaging**<br>FDG<br>PIB<br>**CSF**<br>Ab42<br>Ptau181	MMSE<br>Verbal Logical Memory test<br>CDR<br>Serial Verbal Learning tests and Logical Memory "A" Trail tes	Positive imaging and fluid AD biomar kers	Pathological CSF ab42 in one; and p-tau181 in the other; positive FDG and PIB,	Novel variant in PSEN1, p.T119I; Pathological CSF ab42 in one; and p-tau181 in the other; positive FDG and PIB,
(Jacquier, Arango et al. 2001)	Latam/<br>Colombia/<br>Colombian	83 AD (73.3)<br>44 HC<br>(65.8)	**Genetic**<br>APOE	MMSE	-	Positive association between APOEe4; (OR= 5.1 95%CI 1.9 - 13.6); negative association between epsilon2 and AD (OR= 0.2 95% CI 0.05-0.75).	APOE4 is as risk factor and APOE2 is protective.
(Kretzschmar, Alencar et al. 2021)	Latam/<br>Brazil/<br>Brazilian	150 AD (70.8)<br>114 HC (75.6)	**Genetic**<br>APOE<br>CELF1<br>BIN1–CYP27C1.	MMSE	-	rs769449 (APOE), rs10838725 (CELF1), rs6733839, and rs744373 (BIN1–CYP27C1).	From 19 only 4 SNPs associated with AD in Europeans were associated in the Brazilian cohort.
(Kretzschmar, Antoniazzi et al. 2020)	Latam/<br>Brazil/<br>Brazilian	162 AD (?)<br>137 HC (?)	**Genetic**<br>CR1	MMSE	-	Homozygosity for haplotype rs3849266*C_rs2274567*A (CA/CA genotype) was associated with susceptibility to AD (OR = 2.94, p = 0.018;	CR1<br>haplotype rs3849266*C_rs2274567*A (CA/CA genotype) patienets were more susceptible to AD.
(Lee, Cheng et al. 2011)	Caribbean/<br>Caribbean Hispanic	549 AD (78.87)<br>544 HC (78.94)	**Genetic**<br>APOE	-	-	rs9945493 on 18q23 and 22 additional SNPs were significant under 3 different analyses: unadjusted and stratified by the presence or absence of the APOE ε4 allele.	rs9945493 on 18q23 and 22 additional SNPs were significant under 3 different analyses: unadjusted and stratified by the presence or absence of the APOE ε4 allele.
(Lee, Cheng et al. 2008)	Caribbean/<br>Caribbean Hispanic	589 AD (589)<br>499 at-risk relatives (499)	**Genetic**<br>NCAPD2<br>GAPDH			rs740850 in NCAPD2 (p = 0.0097) and rs1060620 in GAPDH (p = 0.042)were associated with AD in Caribbean Hispanics:	rs740850 in NCAPD2 and rs1060620 in GAPDH were associated with AD Caribbean Hispanics

(Lee, Cheng et al. 2007)	Caribbean/ Caribbean Hispanic	178 AD (80.9) 194 HC (79.1)	**Genetic** APOe SORL1	-	-	SNP 12, near the 5' region, was associated with AD in African American and Hispanic individuals. A single haplotype in the 3' region was associated with AD in Hispanic individuals.	There is an association between genetic variants in SORL1 and AD.
(Li, Rowland et al. 2005, Livney, Clark et al. 2011)	Caribbean/ Caribbean Hispanic	154 AD (68)	**Genetic** APOE	MMSE DSRS CDR GDS	-	The apolipoprotein E-4 genotype was not associated with AD in the Latino cohort.	The apolipoprotein E-4 genotype was not associated with AD in the Latino cohort.
(Llibre-Guerra, Li et al. 2023)	LAC/ Multiple/ Cubans, Dominican republicans, Puerto Ricans, Venezuelan	5,947 AD (74.8)	**Genetic** APOE	CSI-D Verbal fluency task TICS-M Delayed recall	-	APOEε4 carriers had higher dementia prevalence.	APOEε4 was associated with increased relative risk of dementia and lower memory performance.
(Lopera, Marino et al. 2023)	Latam/ Colombia/ Colombian	1 ADAD (72)	**Genetic** RELN **Imaging** PIB, FTP and FDG	MMSE Naming CERAD word list learning/recall/recognition/Praxis Recall Semantic fluency FAS Raven's GDS EDG	PIB, FTP and FDG	RELN (H3447R)	Amyloid and tau positive but FDG negative<br>RELN (H3447R) confers resilience to ADAD dementia
(Lozano-Tovar, Rodriguez-Agudelo et al. 2023)	Latam/ Mexico/ Mexican	31 AD (73) 31 HC (69)	**Genetic** APOe PERIOD genes	MOCA NPI, PHQ-9 sleeping disorders questionnaire	-	APOE_ε4 allele is an AD risk variant;<br>PER3_rs228697 variant showed a increased risk for circadian rhythm sleep-wake disorders in Mexican AD;<br>gene-gene interaction analysis identified a novel interaction between PERIOD and APOE gene variants.	APOE_ε4 allele is an AD risk variant and interacts with PERIOD genes
(Lucatelli, Barros et al. 2011)	Latam/ Brazil/ Brazilian	35 AD (74) 85 HC (68)	**Genetic** APOE APOC1	MMSE	-	The haplotypes E*3/-317*ins and E*4/-317*ins of APOE/APOC1 genes were significantly more frequent in the groups with AD. Genotype frequencies were only different in groups without the E*4/-317*ins haplotype (P = 0.012 for AD)	APOE/APOC1 genes were more frequent in the AD groups
(Marca-Ysabel, Rajabli et al. 2021)	Latam/ Peru/ Peruvian	79 AD (72.3) 128 HC (75.0)	**Genetic** APOE	-	-	ε4 allele is associated with increased risk of AD in the Peruvian population (odds ratio = 5.02, confidence interval: 2.3-12.5, p-value = 2e-4)	Risk for AD from ApoE ε4 in Peruvians is higher than we have observed in non-Hispanic white populations.

(Martinez, Ochoa et al. 2022)	Latam/ Venezuela/ Venezuelan	108 AD (83)<br>40 MCI (70)<br>96 HC (68)<br>23 AD indigenous (68, Kamarata-Kanaimö - KK)	**Genetic** APOE	-	-	Frequencies:<br>HC and MCI were ε3/ε3> ε3/ε4> ε2/ε4> ε3/ε2> ε4/ε4;<br><br>AD ε3/ε3> ε3/ε4> ε4/ε4> ε2/ε4> ε3/ε2;<br><br>KK, ε3/ε3> ε3/ε4> ε4/ε4; no allele ε2	APOE allelic and genotypic frequencies showed a similar distribution to other studies in Venezuela and the Americas. the ε2 allele was absent in the the indigenous community
(Molero, Pino-Ramirez et al. 2001)	Latam/ Venezuela/ Venezuelan	121 AD (83)<br>VD (34)<br>HC (66)	**Genetic** APOe	-	-	ApoE4 was higher in AD (P = 0.008), but not VD (P = 0.469) compared to HC;<br><br>Carriers of at least one e4 allele were at higher risk for AD, but not for VD;<br><br>Homozygous for e4 had a higher risk of developing AD (OR = 4.23) than were heterzygous	Carriers of at least one ε4 allele were at higher risk for AD, not for VD;<br><br>The risk of AD conferred by APOE-ε4, adjusted for age and stratified by gender, was significant only for women.<br><br>No association was found between the ε-2 allele and AD or VD.
(Montufar, Calero et al. 2017)	Latam/ Ecuador/ Ecuadorian	56 AD (78)<br>58 HC (76)	**Genetic** Apoe	MMSE	-	Allelic and genotypic frequencies follow the trends observed in most worldwide populations, with APOE ε4 allele being greater forAD (33.9%) than for HC (6.0%). They also found a high-risk association between APOE ε4 allele carriers and LOAD (OR = 7.286; 95% CI = 2.824-18.799; p < 0.001).	APOE ε4 is a genetic risk for LOAD in the Ecuadorian Mestizo population
(Moraes, Benedet et al. 2013)	Latam/ Brazil/ Brazilian	120 AD (72)<br>412 HC (70)	**Genetic**<br>ApoE<br>*L1-α*<br>*IL1-β*,<br>*IL6*,<br>*IL8*,<br>*IL10*,<br>*IL12-β*,<br>*IL18*,<br>transforming growth factor (*TGF*)-β_1,<br>toll-like receptor (*TLR*)-4<br>*TNF*	MMSE<br>CDR	-	40% lower chance of AD (p = 0.004) among homozygotes of the *IL10* -1082A allele (rs1800896).	The *IL10* locus seems to affect the onset of AD in a context sensitive to the genetic ancestry of Brazilian older adults.

(Morelli, Leoni et al. 1996)	Latam/ Argentina/ Argentinian	45 AD (74)<br>45 HC (71)<br>101 Young HC (33)	**Genetic** APOE4	-	-	£4 allele frequencies for AD (0.22) HC 0.077. The OR for our case control study, in the presence of one or two £4 alleles, was 3.33 (95% CI 1.204-9. 020).	Association between e4 allele and LOAD in the Hispanic population from South America
(Moreno, Pino et al. 2017)	Latam/ Colombia/ Colombian	280 AD (75)<br>357 HC (71)	**Genetic** APOE	-	Greater percentage of APOEε4 carriers was found AD (46.1%) compared to HC (22.8%)	African ancestry was associated with an increased LOAD risk (OR 1.55; P=0.029), whereas Native American ancestry was associated with lower risk (OR: 0.75; P=0.046). Significant differences in the proportion of Native American ancestry between carriers and noncarriers of the APOE4 allele (P=0.047)	Variants of African Colombians and different from APOE4 that represents a risk factor for the development of LOAD, whereas variants of Native American origin confers protection
(Moreno, Ruiz et al. 2017)	Latam/ Colombia/ Colombian	280 AD (75)<br>357 HC (71)	**Genetic**<br>APOE<br>BIN1<br>CLU<br>PICALM<br>ABCA7<br>CD33			BIN1 (rs744373, OR: 1.42), CLU (rs11136000, OR: 0.66), PICALM (rs541458, OR: 0.69), ABCA7 (rs3764650, OR: 1.7), and CD33 (rs3865444, OR: 1.12).	Interaction effect was observed between CLU and CR1 variants with APOE.
(Muchnik, Olivar et al. 2015)	Latam/ Argentina/ Argentinian	2 families (2 Argentine pedigrees)	**Genetic** PSEN2	MMSE<br>GDS<br>CDR<br>FAST		the first families from Argentina with the PSEN2 p.N141I mutation	The first report of 2 Argentine EOFAD families with the missense PSEN2 mutation (p.N141I)
(Nishimura, Guindalini et al. 2005)	Latam/ Brazil/ Brazilian	210 AD (70)<br>207 HC (70)	**Genetic**<br>MAOA<br>5-HTTLPR<br>ApoE	MMSE<br>CDR	-	MAOA (AD vs HC: p = 0.01); 5-HTTLPR (= 0.05) allele frequencies	Combination of the MAOA allele 1 + the short allele of 5-HTTLPR + ApoE-4 was more frequent in AD.
Nishimura et al. 2004	Latam/ Brazil/ Brazilian	188 AD (68)<br>188 HC (72)	**Genetic**<br>BNDF	MMSE<br>CDR	-	APOE polymorphism showed an association between the e4 allele and AD (p < 0.0001, OR = 0.39)	Association between the APOE-4 polymorphism and LOAD but BDNF C-270T polymorphism is not an important risk for LOAD in Brazilian patients

(Olarte, Schupf et al. 2006)	LAC/ Caribbean countries/ Dominicas and Puerto Ricans.	11 FAD (85)<br>163 SAD (85)<br>406 HC (76)	**Genetic**<br>APOE	-	-	A significant dose effect of the APOE ε4 allele was present for age at onset in FAD (P = .001) but not in SAD.	ApoEε4 had a consistent lowering effect on age at onset of FAD, but this was attenuated in SAD.
(Ortega-Rojas, Arboleda-Bustos et al. 2022)	Latam/ Colombia/ Colombian	50 AD (?)<br>50 HC (?)	**Genetic**<br>TOMM40<br>APOE<br>APOC1	MoCA<br>IFS<br>GDS	-	Association between LOAD and genetic variants at the TOMM40, APOE and APOC1 were identified.	Genetic variants and haplotypes near the APOE locus are related to LOAD risk and accelerated onset of LOAD in the Colombian population.
(Ortega-Rojas, Morales et al. 2016)	Latam/ Colombia/ Colombian	181 AD (74)<br>181 HC (74)	**Genetic**<br>APOE<br>TOMM40<br>CR1<br>PVRL2<br>SORL1<br>PICALM, GWA_14q32.13 (rs11622883)	-	-	Association of the APOE4 allele with AD;<br>association of rs2075650 TOMM40 with LOAD;<br>TOMM40 allele rs2075650-G have an average age of disease onset of 6 years earlier ;	s2075650 of TOMM40 could be involved in earlier presentation of LOAD in the Colombian population.
5		12 AD (82)<br>33 MCI (79)<br>41 HC (76)	Genetics<br>APOE<br>Imaging<br>Doppler ultrasound	MMSE<br>Digit Span forward<br>Trail Making Test part A<br>executive function<br>Trail Making Test part B<br>Digit Span Backward<br>10 word free recall immediate and delayed list<br>Boston Naming Semantic Verbal Fluency<br>Clock Drawing copy version<br>Poppelreuter Test<br>FAQ<br>GDS-30<br>EGQ-5D-3L.	Ultrasound assessment of carotid IMT and stenosis	-	Increased IMT was associated with poorer performance in memory and those with carotid stenosis showed poorer performance in language, visuospatial abilities and attention, independent of age, education or ApoE ε4 expression.

(Parra-Bonilla, Arboleda et al. 2003)	Latam/ Colombia/ Colombian	25 EOAD (58)<br>54 LOAD (71)<br>67 HC (71.47)	**Genetic**<br>APOE	MMSE	-	The polymorphism −491A/T confers increased risk for AD associated with AA genotype independent of APOEe4 allele (odds ratio (OR): 2.64) and more pronounced in men (OR: 6.07).	The present findings support the hypothesis of an independent role of the APOE promoter polymorphisms in the modulation of the risk for AD.
(Pastor, Roe et al. 2003)	Latam/ Colombia/ Colombian	57 AD E280A PS1 mutation carriers (?)<br><br>62 E280A PS1 mutation carriers (?)	**Genetic**<br>APOE	-	-	APOE ε4 allele carriers were more likely to develop AD at an earlier age than subjects without the ε4 allele (hazard ratio, 2.07; 95% confidence interval, 1.07–3.99; p = 0.030).	Promoter APOE variants did not influence either the onset or the duration of the disease.
(Payao, Goncalves et al. 2012)	Latam/ Brazil/ Brazilian	99 AD (75.31)<br>165 HC (71,67)<br>122 young HC (20.76)	**Genetic**<br>IL-1RN	-	-	-511C/-31T/2-repetitions VNTR (IL-1RN) haplotype had a protective effect for AD when compared to EC (p=0.005);<br>-511C/-31C/1-repetition VNTR haplotype was associated as a risk factor for AD (p=0.021)	Relevant role of IL-1 genes cluster in AD pathogenesis in this Brazilian population.
(Paz, Garcia-Cardenas et al. 2015)	Latam/ Ecuador/ Ecuadorian	56 AD (73.12)<br>55 HC (72.38)	**Genetic**<br>CST3<br>CTSD<br>MnSOD	-	-	Positive association between a CTSD polymorphism (Ala224Val) and the development of AD (OR = 8.1; P < 0.025).	Variations in CTSD and MnSOD showed no association with the development of AD, whereas the presence of the Ala224Val polymorphism in CTSD had a positive association with the development of AD.
(Paz-y-Mino, Carrera et al. 2010)	Latam/ Ecuador/ Ecuadorian	39 AD (78.4)<br>39 HC (77.9)	**Genetic**<br>APOE<br>GPX1	-	-	Positive association between ε4 and ε2 alleles of Apo E.<br><br>The GPX1 gene shows an association of leu allele, whereas pro allele shows a negative association.	ApoE is not a risk factor, nor a protective one for AD, whereas the leu allele of GPX1 is a possible risk factor for the disease.
(Pereira, Alvim-Soares et al. 2016)	Latam/ Brazil/ Brazilian	249 AD (?)<br>112 HC (?)	**Genetic**<br>PER2<br>PER3<br>CLOCK<br>OX2R	-	-	The rs9370399 (OX2R) has also shown an association between A allele (p=0.03, OR= 1.4) and AD, which not resisted 1000 permutations.	PER2, PER3, CLOCK and OX2R polymorphisms were not associated with AD.
(Pereira, Romano-Silva et al. 2012)	Latam/ Brazil/ Brazilian	42 SAD with BPSD (82)<br>56 SAD with no BPSD (78)<br>113 HC (78)	**Genetic**<br>COMT	MMSE	-	COMT SNP were Not associated with AD	COMT SNP were Not associated with AD

(Pimentel, Gomes da Cunha et al. 2014)	Latam/ Brazil/ Brazilian	58 AD (77) 73 HC (76.5)	**Genetic** PRNP	-	-	No association between the PRNP polymorphism at codon 129 and AD	No association between the PRNP polymorphism at codon 129 and AD
Poblete et al. 2019	LAC/Puerto Rico/Puerto Rican	1 ADAD	**Genetic** PSEN1	-	-	Identification of G206A PSEN1 mutation	Identification of G206A PSEN1 mutation
(Quiroga, Calvo et al. 1999)	Latam/Chile/Chilea n	95 AD (80.7) 187 HC (78.2)	**Genetic** APOE	-	-	Frequencies: HC APOE (e2 = 0.07, e3 = 0.74 and e4 = 0.19) AD APOE (e2 = 0.08, e3 = 0.52 and e4 = 0.40.) OR for e4 carriers vs non-e4 carriers was 2.9 (95% CI 1.7-5.1); OR e4/e4 vs e3/e3 was 12.8 (95% CI 3.9-47.6).	Association between ApoE e4 allele with late-onset AD in a Chilean population.
(Rajabli, Feliciano et al. 2018)	LAC/ Puerto Rico/ Puerto Ricans	220 AD (75.1) 169 HC (73.6)	**Genetic** APOE	MMSE Modified MMSE	-	global ancestry showed no interaction with *ApoE* risk (Puerto Rican: p-value = 0.49); ancestry local to the *ApoE* region showed an interaction with the *ApoE* ε4 allele in both populations (Puerto Rican: p-value = 0.019;	Lower risk effect in the *ApoE*ε4 allele are likely due to ancestry-specific genetic factors near *ApoE*
(Ramirez Aguilar, Acosta-Uribe et al. 2019)	Latam/ Colombia/ Colombian	93 (26 carriers of the Ile416Thr variant)	**Genetic** PSEN1 **Imaging** PIB FTP	MMSE FAST	Amyloi d and Tau positivit y	A missense variant in PSEN1 (NM_000021.3: c.1247T.C p.Ile416Thr) was identified.	Ile416Thr is a novel pathogenic variant that causes AD in the sixth decade of life
(Rasmussen, Delabio et al. 2013)	Latam/ Brazil/ Brazilian	200 AD (75.3) 165 HC (71.67)	**Genetic** APOE IL-6	MMSE CDR	-	No association was found between any IL-6 polymorphism and AD; haplotype composed of the -597 A allele and the -174G allele indicated a crude OR of 0.15 (p = 0.0021) and an adjusted OR (adjusted for the APOE E4 allele value) of 0.15 (p = 0.00294);	The findings revealed a protective effect of AG (-597A, -174G) haplotype, which worked independently of the APOE E4 allele in our Brazilian population sample.
(Reiman, Quiroz et al. 2012)	Latam/ Colombia/ Colombian	20 PSEN1 E280A mutation carriers (22) 24 non-carriers (22)	**Genetic** PSEN1 APOE **Imaging** MRI **Fluid** CSF and Plasma AB1-42	MMSE Verbal Fluency Naming Word memory Word Recognition	-	The carrier and non-carrier groups did not diff er significantly in their dementia ratings, neuropsychological test scores, or proportion of APOE ε4 carriers;<br><br>Carriers had greater right hippocampal and parahippocampal activation ((p=0·001 and p<0·014) less precuneus and posterior cingulate deactivation (p=0·009);<br><br>Carries have higher CSF Aβ1–42 concentrations (p=0·008) and plasma Aβ1– 42 concentrations (p=0·01) than non-carriers.;	Young adults at genetic risk for ADAD have functional and structural MRI findings and CSF and plasma biomarker fi ndings consistent with Aβ1–42 overproduction

(Reitz, Cheng et al. 2012)	LAC/Multiple countries/Caribbean Hispanics		**Genetic**<br>IDE-KIF11-HHEX complex<br><br>**Plasma**<br>plasma Ab40 and Ab42	MMSE	-	3 SNPs in IDE (rs2421943, rs12264682, rs11187060) were associated with plasma Ab40 or Ab42 levels in single marker and haplotype analyses;	Association in the IDE region on chromosome 10q with Ab40 and 42 levels.
(Reitz, Conrad et al. 2012)	LAC/Multiple Countries/Caribbean Hispanics	549 AD (79.98)<br>544 HC (78.87)	**Genetic**<br>LRRTM3	MMSE<br>Boston Naming Test<br>Controlled Word Association Test<br>Boston Diagnostic Aphasia Evaluation<br>WAIS-R Similarities subtest<br>Mattis Dementia Rating Scale<br>Rosen Drawing Test<br>Benton Visual Retention Test<br>Selective Reminding Test<br>Total recall, long-term recall, long-term storage, continuous long-term storage, words recalled on last trial, delayed recall, and delayed recognition.	-	One SNP in the promoter region (rs16923760; C allele: odds ratio,−0.74, P = .03), and a block of 4 SNP in intron 2 (rs1925608, C allele: 0.84, P=.04; rs7082306, A allele: 0.75, P = .04; rs1925609, T allele: 1.2, P = .03; and rs10997477, T allele: 0.88, P = .05) were associate to AD.	Genetic variation in LRRTM3 is associated with AD risk
(Reitz, Lee et al. 2011)	LAC/<br>Multiple Countries/<br>Caribbean Hispanics	549 AD (79.98)<br>544 HC (78.87)	**Genetic**<br>*SORCS1*	MMSE<br>Boston Naming Test<br>Controlled Word Association Test<br>Boston Diagnostic Aphasia Evaluation<br>WAIS-R<br>Similarities subtest	-	3 SNPs in intron 1, were associated with memory retention in the Caribbean Hispanic (rs10884402(A allele:β=−0.15,p=0.008), rs7078098(C allele:β=0.18,p=0.007) and rs950809(C allele:β=0.17,p=0.008)) (0.002<p<0.03).	Variation in intron 1 in *SORCS1* is associated with memory changes in AD.

				Mattis Dementia Rating Scale<br>Rosen Drawing Test<br>Benton Visual Retention Test<br>Selective Reminding Test<br>Total recall, long-term recall, long-term storage, continuous long-term storage, words recalled on last trial, delayed recall, and delayed recognition.			
(Reitz, Tosto et al. 2012)	LAC/<br>Multiple Countries/<br>Caribbean Hispanics	549 AD (79.98)<br>544 HC (78.87)	Genetic<br>FTO gene	MMSE<br>Boston Naming Test<br>Boston Diagnostic Aphasia Evaluation<br>the Wechsler Adult<br>Mattis Dementia Rating Scale<br>the Rosen Drawing Test<br>the Benton Visual Retention<br>Reminding Test	-	Three SNPs (rs17219084, rs11075996, rs11075997, FDR p-value: 0.009<p<0.01) that were associated with AD.	genetic variation in Introns 1 and 2 of the FTO gene may contribute to AD risk.
(Ringman, Medina et al. 2011)	Latam/Mexico/Mexicans	14 FAD MCs (29.9)<br>9 HC (37.3)	**Genetic**<br>APOE<br>**Imaging**<br>fMRI	CDR	-	FAD MCs showed decreased BOLD activation in the anterior cingulate gyrus relative to 9 HCs<br>Four APOE ε3/4 carriers demonstrated increased BOLD signal compared with 14 ε3/3 carriers in the occipital and perisylvian cortices bilaterally.	Increased fMRI activation was associated with APOE genotype but not with FAD mutations.
(Romas, Mayeux et al. 2000)	LAC/Multiple Caribbean Countries/Puerto Ricans, Dominicans	Caribbean Hispanic families.<br>100 affected (?)<br>47 unaffected (?)	**Genetic**<br>APOE<br>A2M	-	-	Odds of having the alpha2m deletion/insertion polymorphism was increased 3-fold for family members with Alzheimer's disease compared to healthy family members, rising to 5-fold after adjusting for APOEe4;<br>There was no relationship between the alpha2m Val1000Ile polymorphism and AD in these families.	Alpha2m and AD found in the Caribbean Hispanic families suggest that the overall effect of this gene on susceptibility is small and may be limited to certain populations or families.

(Romas, Santana et al. 2002)	LAC/Puerto Rico-Dominic Republican/Caribbean Hispanic	Families<br>218 Unaffected (?)<br>132 CDR 0.5 (?)<br>72 Other Dementia (?)<br>306 AD (?)	Genetic<br>APOe4	CDR	-	APOe4 is associated with AD	Late-onset familial AD among Caribbean Hispanic is strongly associated to APOe4.
(Santos-Reboucas, Abdalla et al. 2009)	LATAM/<br>Brazil/<br>Brazilian	180 (77.06)	**Genetic**<br>LRRK2	MMSE		LRRK2<br>p.G2019S in one AD patient with no PD signs.	This mutation is not a common etiological factor for AD in Brazil
(Santos-Reboucas, Goncalves et al. 2017)	LATAM/<br>Brazil/<br>Brazilian	174 AD (77,15)<br>166 PD (69)<br>176 HC (70,7)	**Genetic**<br>APOE<br>PICALM<br>CR1<br>CLU<br>APOE	-		Significant differences in genotype and allele frequencies for the SNP PICALM rs3851179 between AD/PD cases and controls, but none for CR1 rs6656401 and CLU rs11136000;<br><br>After<br>stratification by APOEe4 status, the protective effect of the PICALM rs3851179 a allele in AD cases remains evident only in APOE e4 (-) carriers, suggesting that the APOE e4 risky allele weakens its protective effect in the APOE e4 subgroup.	Significant differences in genotype and allele frequencies for the SNP PICALM rs3851179 between AD/PD cases and controls, but none for CR1 rs6656401 and CLU rs11136000;<br><br>After<br>stratification by APOEe4 status, the protective effect of the PICALM rs3851179 a allele in AD cases remains evident only in APOE e4 (-) carriers, suggesting that the APOE e4 risky allele weakens its protective effect in the APOE e4 subgroup.
Steven Sevush., 2000	Caribbean/<br>Cuba/	140 AD (?)<br>49 HC (?)	**Genetic**<br>APOE	-		ApoEe4 allele frequencies were greater for patients with AD than for control subjects, both for white non-Hispanic (P=0.005) and for Cuban American subjects (P =0.01).<br><br>The relative risk of AD conferred by the ApoEe4 allele was somewhat greater for Cuban Americans (OR4.33; range: 1.27–14.79) than for white non-Hispanics (OR3.40), but the difference was not statistically significant (P = 0.1).	ApoEe4 allele frequencies were greater for patients with AD than for control subjects for Cuban American subjects.<br><br>The relative risk of AD conferred by the ApoEe4 allele was greater for Cuban Americans than for white non-Hispanics but the difference was not statistically significant.

(Shang, Lv et al. 2015)	Caribbean	1175 (?)	**Genetic**<br>APOE<br>APOC1<br>TNFRSF1A<br>LRP1B<br>CDH1<br>TG<br>CASP7	-		TNFRSF1A, CDH1, CASP7, LRP1B and TG were associatied with AD susceptibility in Caribbean Hispanic individuals.	This is the first genetic association study which showed the significant association between these five genes and AD susceptibility in Caribbean Hispanic individuals.
(Simao-Silva, Bertolucci et al. 2013)	LATAM/<br>Brazil/<br>Brazilian	82 AD (74,5)<br>78 HC (71,7)	**Genetic**<br>BCHE			The allele, genotype and haplotype frequencies of the K and the 116A variants of BCHE gene were not significantly different between cases and controls.	The allele, genotype and haplotype frequencies of the K and the 116A variants of BCHE gene were not significantly different between cases and controls.
(Smid, Landemberger et al. 2013)	LATAM/<br>Brazil/<br>Brazilian	99 AD (66,7)<br>111 HC (68,5)	**Genetic**<br>APOE<br>PRNP	MMSE		Codon 129 genotype distribution in AD 45.5% methionine (MM), 42.2% methionine valine (MV), 12.1% valine (VV); and 39.6% MM, 50.5% MV, 9.9% VV among controls (p>0.05). There were no differences of cognitive performance concerning codon 129. Stratification according to ApoE genotype did not reveal difference between groups.	Stratification according to ApoE genotype did not reveal difference between groups.
(Souza, de Godoy et al. 2003)	LATAM/<br>Brazil/<br>Brazilian	68 AD<br>39 other dementias<br>58 HC<br>(age 65-82)	**Genetic**<br>APOE	-		APOE ε2 frequency was lower in AD (1%), and the ε3 allele and ε3/ε3 higher in HC (84 and 72%, respectively) as were the ε4 allele and ε3/ε4 genotype frequencies in AD (25 and 41%, respectively).	The higher frequency of the ε4 allele in AD confirmed its role as a risk factor, while ε2 provided a weak protection against development of the disease.
(Takada, Alaez-Verson et al. 2022)	Latam/<br>Multiple /Mexican-Brazilians	DIAD (43.5)	**Genetic**<br>PSEN1	MMSE<br>MoCA<br>CDR<br>FAQ	-	PSEN1 p.Val103_Ser104delinsGly, p.Lys395Ile, p.Pro264Se, p.Ala275Thr, and p.Ile414Thr variants have not been reported DIAD families.	5 novel variants in the presenilin1 (PSEN1) gene from Brazilian and Mexican families.
(Tang, Alaniz et al. 2020)	Caribbean Hispanic	302 AD (?)<br>49<br>Unnafected (?)	**Genetic**<br>CDH23<br>SLC9A3R1<br>RHBDD2<br>ITIH2	-	-	CDH23, SLC9A3R1, RHBDD2, contribute to AD etiology.	CDH23, SLC9A3R1, RHBDD2 contribute to AD etiology.

(Teresa, Fernado et al. 2020)	Latam/ Mexico/ Mexicans	221 AD (76.8) 534 HC (71.4)	**Genetic** ABCA1	MMSE CDR	-	BCA1 polymorphisms located in the exonic region (rs2230808, rs2066718, rs2230806) and two in the promoter region (rs1800977, rs2422493). They found one protective haplotype: CCCCGC (OR = 0,502, p < 0.001), and one risk haplotype TCCCAT (OR = 2208, p < 0.000) for the development of dementia.	ABCA1 plays a role in the pathophysiological mechanisms for the development of dementia.
(Tosto, Fu et al. 2015)	Caribbean Hispanic	Unrelated sample AD 78.6 (?) HC 73.5 (?) Related sample AD 78.9 (?) HC 73.4 (?)	**Genetic** APOE FBXL7	-	-	rs75002042 in FBXL7 (5p15.1), was found genome-wide significant in the case–control cohort and confirmed in the related members cohorts.	rs75002042 in FBXL7 was found genome-wide significant in the case–control cohort and confirmed in the related members cohorts.
(Tycko, Lee et al. 2004)	African American/ Caribbean Hispanic	AD (82.3) HC (78.2)	**Genetic** APOE APOC1	-	-	The APOC1 HpaI+ variant was associated with AD in Caribbean Hispanic individuals, but linked to the APOE ε4 allele. Estimated haplotypes including −219G/T, APOE, and APOC1 differed significantly in Caribbean Hispanic patients and controls but not in African American participants.	The APOC1 HpaI+ variant was associated with AD in Caribbean Hispanic individuals, but linked to the APOE ε4 allele. Estimated haplotypes including −219G/T, APOE, and APOC1 differed significantly in Caribbean Hispanic patients and controls but not in African American participants.
(Velez, Lopera et al. 2019)	Latam/ Colombia/ Colombian	78 sAD (?)	**Genetic** APOE ASTN2 SNTG1	-	-	Two key epistatic interactions between the APOEe2 allele and SNPs ASTN2-rs7852878 and SNTG1-rs16914781 that delay AOO by up to ~ 8 years (95% CI 3.2–12.7, P = 1.83 × 10−3) and ~ 7.6 years (95% CI 3.3–11.8, P = 8.69 × 10−4).	Interactions between the APOEe2 allele and SNPs ASTN2-rs7852878 and SNTG1-rs16914781 that delay AOO by up to 8 years
(Velez-Pardo, Rojas et al. 2015)	Latam/ Colombia/ Colombian ("Paisa" population)	HC 1001	**Genetic** APOE			APOEe3/3 genotype presented the highest frequency (66.33%) and the APOEe4/4 had the lowest frequency (1.89%). Allele frequencies obtained for APOEe2, APOEe3 and APOEe4 were 0.075, 0.814 and 0.111, respectively.	Genotype frequencies comply with Hardy–Weinberg expectations.

(Vendramini, de Labio et al. 2011)	Latam/ Brazil/ Brazilian	199 AD (73.81)<br>146 EC (71.25)<br>95 YC (20.56)	**Genetic**<br>APOE<br>IL-8<br>IL-1			The allele frequencies did not differ significantly among groups.<br><br>The APOEe4 allele was strongly associated with AD ($p < 0.001$).<br><br>No association of AD with either the IL-1 or the IL-8 polymorphism was observed.	The allele frequencies did not differ significantly among groups.<br><br>The APOEe4 allele was strongly associated with AD<br>No association of AD with either the IL-1 or the IL-8 polymorphism was observed.
(Vieira, Magalhaes et al. 2015)	Latam/ Brazil Brazilian/	269 AD (78)<br>114 HC (76)	**Genetic**<br>GAB2<br>BDNF<br>APOE ε4	MMSE		GAB2 and BDNF were not associated with AD in our sample. Nevertheless BDNF Val allele (rs6265) presented a synergic association with the APOE ε4 allele.	GAB2 and BDNF were not associated with AD in our sample. Nevertheless BDNF Val allele (rs6265)<br>presented a synergic association with the APOE ε4 allele.
(Villarreal, Grajales et al. 2016)	LAC/ Panama/ Panamanian	31 AD (82,1)<br>43 MCI (80,2)<br>185 HC (76,7)	**Genetic**<br>ApoE	MMSE		At least half of AD (57%) and MCI (50%) groups expressed one or<br>two copies of the ApoEe4 allele.<br>Age (OR = 2.53,<br>$p = 0.042$) and ApoE ε4 expression (OR = 5.14, $p < 0.001$) were significant<br>predictors of cognitive.	Significant differences in cognitive performance were noted among groups;<br><br>Most of AD and MCI expressed one or<br>two copies of the ApoEe4 allele.<br><br>Age<br>and ApoEe4 expression<br>were significant<br>predictors of cognitive.
(Yescas, Huertas-Vazquez et al. 2006)	Caribbean/ Mexico/ Mexican	97 related (40)<br>100 unrelated (76)	**Genetic**<br>APOE<br>PSEN1	MMSE		The Ala431Glu mutation in exon 12 of PSEN1 was found in nine (75%) of these families	The Ala431Glu mutation in exon 12 of PSEN1 was found in nine (75%) of these families, which segregated showing autosomal dominant inheritance.
(Zhang, Farrell et al. 2022)	Caribbean	AD: 5519 (European ancestry) (?)<br>and 4917 (Caribbean Hispanic) (?)<br>HC: 218 (European ancestry) (?)	**Genetic**<br>mtDNA	-		Significant association of AD with a rare MT-ND4L missense variant (rs28709356; minor allele frequency = 0.002; $P = 7.3 \times 10^{-5}$) as well as with MT-ND4L in a gene-based test ($P = 6.71 \times 10^{-5}$). Significant association was also observed with a MT-related nuclear gene, TAMM41, in a gene-based test ($P = 2.7 \times 10^{-5}$). The expression of TAMM41 was lower in AD cases	Significant findings in *MT-ND4L* and *TAMM41* provide evidence for a role of mitochondria in AD.

		and 177 (Caribbean Hispanic) (?)				than controls (P = .00046) or mild cognitive impairment cases (P = .03).	

Supplementary Table 5: Fluid Biomarker Studies (Plasma/CSF) in Africa							
**Study**	**Regions/Country/ population**	**Sample disease (mean age)**	**Biomarker** (type, markers)	**Cognitive measures**	**Brain measures**	**Biomarker results**	**Multimodal results**
Smach et al (2011)(14)	Tunisian Arab descendants who live at the center of Tunisia	70 probable AD (NINCDS-ADRDA), 33 non-AD (consisting of 16 vascular dementia, 7 mixed dementia, 5 FTD, 3 LBD and 2 unclassified dementia)	CSF Aβ1-42 and T-tau, **Folate** and Homocysteine (Hcy)	MMSE (validated Arabic version)	Brain MRI	Levels of Aβ1-42, T-tau and folates in CSF were significantly different between groups, but not Hcy.<br>Average folate in CSF was lower in AD patients compared with controls (18.7 ± 2.4 vs. 20.3 ± 1.7 nmol/l, Bonferroni-corrected p value < 0.02).<br>There was no correlation between Aβ1-42 or T-tau and folate or Hcy in CSF, regardless of the group.<br>In the AD group, there was a significant inverse correlation between Hcy and folate in CSF ($\rho = -0.63$, $p < 0.0001$), whereas in the nAD group, a significant correlation was found for Hcy between plasma and CSF ($\rho = 0.59$, $p < 0.0005$).	
Schwinne et al (2023) Pre-print	Democratic Republic f Congo	AD 81 participants (43 with AD, 38 healthy controls)	Aβ40, Aβ42, Aβ42/40, p-tau 181	CSID, AQ		Plasma Aβ42/40 was significantly associated with lower CSID scores and higher AQ scores, indicative of AD (p<0.001). These relationships were observed in healthy controls (CSID p=0.01, AQ p=0.03), but not in dementia cases. However, p-tau 181 did not exhibit significant associations with either measure. Factors such as age, sex, education, presence of APOE e4 allele, did not alter these relationships.	

Supplementary Table 6: Cognitive Studies in Africa							
**Study**	**Regions/Country/ population**	**Sample disease (mean age)**	**Biomarker** (type, markers)	**Cognitive measures**	**Brain measures**	**Biomarker results**	**Multimodal results**
Gray et al (2014)(19)	6 villages in Hai District, Tanzania	Sample size = 60<br>11 (18,3%) DSM-IV dementia		Identification and Intervention for Dementia in Elderly Africans (IDEA) Study Dementia Screening Instrument		AUROC (95% CI): 0.888 (0.766-1.000)<br>Dementia cutoff: < 7<br>Sensitivity: 81.8%<br>Specificity: 84.4%	
Paddick et al (2018)(20)	Tanzania	73 (17.2%) of 424 study participants had DSM-IV dementia		IDEA Cognitive screen		Diagnostic accuracy: 0.874 (95% CI: 0.826-0.907)<br>At cut-off ≤ 7<br>● Sensitivity = 0.82<br>● Specificity = 0.77<br>● PPV = 0.56<br>● NPV = 0.92<br>At cut-off ≤ 8<br>● Sensitivity = 0.88<br>● Specificity = 0.71<br>● PPV = 0.52<br>● NPV = 0.94	
Paddick et al (2015)(21)	Moshi, Tanzania and Ibadan, Nigeria	**Outpatients Nigeria**<br>12 major cognitive impairment (median age: 71 (65.3 - 77.5))<br>109 without major		IDEA Cognitive screen		**At cut-off of ≤ 7**<br>**Nigerian Outpatients**<br>● AUROC curve - 0.990<br>● 100% sensitivity<br>● 96.3% specificity<br>**Tanzanian Outpatients**<br>● AUROC curve - 0.919	

		cognitive impairment (median age: 70 (67 - 75.5))<br><br>**Outpatients Tanzania**<br><br>13 major cognitive impairment (median age: 79.5 (73.3 - 89.8))<br><br>46 without major cognitive impairment (median age: 72 (67.3 - 78.8))<br><br>**Inpatients Tanzania**<br><br>33 major cognitive impairment (median age: 78 (72.5 - 90))<br><br>64 without major cognitive impairment (median age: 75.5 (70/3 - 81))				• 61.5% sensitivity<br>• 93.5% specificity<br><br>**Tanzanian Inpatients**<br><br>• AUROC curve - 0.917<br>• 90.9% sensitivity<br>• 87.5% specificity<br><br>**Sensitivity improved in the Tanzanian population using a cut-off of ≤ 8**	

Lenger et al (1996)(22)	South Africa	20 patients and their [20] relatives		The Deterioration Cognitive Observee (DECO)		The DECO scores correctly predicted normal functioning in 7 patients and dementia in 8. The DECO scores correlated with the MMSE scores (r = 0.625; p < 0.01) and MMSE scores correlated with the clinicians' diagnosis (chi 2 = 0.114; df = 1; p = 0.73).<br><br>The DECO was found to predict dementia correctly in all but the severely demented patients.	
Gray et al (2016)(23)	Rural Hai district, Tanzania	93 DSM-IV dementia<br><br>60 no dementia		IDEA Cognitive screen		The AUROC curve was 0.855 (95% CI 0.794 to 0.915) for the 153 people who had a clinical examination for the presence or absence of dementia, see Table 4. At a cut-off of ≤7, sensitivity was 59.0% and specificity 86.0% and at a cut-off of ≤8, sensitivity was 87.2% and specificity 67.5%. Although sensitivity was higher at a cut-off of ≤8 than ≤7, the likelihood ratio was higher at ≤7 than at ≤8 (4.21 and 2.68 respectively).	
Gray et al (2021)(24)	12 representative villages in Kilimanjaro, Tanzania; and 3 communities in Akinyele Local Government, Oyo State, Nigeria	3011 people in Tanzania and 1117 in Nigeria		IDEA cognitive screen		The 50th decile values for IDEA were 13 (60-64 years) vs. 8/9 (above 85 years), 10-11 uneducated vs. 13 primary educated, and 11/12 in females vs. 13 in males. The normative values for 10-word list delayed recall and categorical verbal fluency varied with education [i.e., delayed recall mean 2.8 [standard deviation (SD) 1.7] uneducated vs. 4.2 (SD 1.2) secondary educated; verbal fluency mean 9.2 (SD 4.8) uneducated vs. 12.2 (SD 4.3) secondary educated], substantially lower than published high-income country values.	
Shalash et al (2020)(25)	Cameroon, Egypt and Nigeria	81 PD and 78 controls		Montreal Cognitive Assessment and the Identification and Intervention for Dementia in Elderly Africans cognitive screen<br><br>Aims of the current study were to develop cross-cultural translated and validated Arabic and French versions of a PD screening questionnaire, and determine its diagnostic accuracy for recognition of parkinsonism in early and moderate-advanced PD		The PD screening questionnaire scores were significantly higher in PD (median 8.0, IQR 6.0–10.0) in contrast to controls (0.0, IQR 0.0–0.0) (p<0.0001), with a similar pattern and level of significance across all country sites. In ROC analysis, the questionnaire demonstrated high diagnostic accuracy for PD overall, with an AUC of 0.992 (95% CI 0.981–1.002).	

Hendrie et al (2006)(26)	Yorubas living in Idikan, Ibadan, Nigeria and African Americans in Indianapolis, US	**Ibadan population sample 308 [38 (82.9) dementia cases*, 257 (78.2) without dementia]**<br><br>Indianapolis populations sample 201 [40 (83.4) dementia cases*, 115 (80.7) without dementia]<br><br>*cases met ICD-10 and DSM-III criteria		Clinician Home-based Interview to assess Function (CHIF)		The area under the ROC curve for dementia diagnosis was 0.965 for Indianapolis and 0.925 for Ibadan<br><br>Inter-rater reliability for the CHIF was high (Pearson's correlation coefficient 0.99 in Indianapolis and 0.87 in Ibadan). Internal consistency, in both samples, was good (Cronbach's α 0.95 in Indianapolis and 0.83 in Ibadan). Scores on the CHIF correlated well with the Blessed Dementia scores at both sites (−0.71, $p < 0.0001$ for Indianapolis and −0.56, $p < 0.0001$ for Ibadan) and with the MMSE (0.75, $p < 0.0001$ for Indianapolis and 0.44, $p < 0.0001$ for Ibadan). For all items at both sites, the subjects without dementia performed significantly better than those with dementia.	
Collingwood et al (2014)(27)	Hai district of Tanzania	87 DSM-IV dementia and 43 controls		IDEA-IADL questionnaire		**Lawton IADL scale**<br>AUROC curve (95% CI):0.828 (0.751–0.906)<br>**IDEA-IADL questionnaire**<br>AUROC curve (95% CI): 0.896 (0.842–0.951)<br>**Internal consistency: 0.959**<br><br>**Performance on the IDEA-IADL was not biased with regard to age, gender or education level**<br>**This is the first validation of functional assessment tools for use in Tanzania**	

						**Combined IDEA cognitive screen and IDEA-IADL questionnaire**<br>AUROC curve (95% CI): 0.937 (0.896–0.979)	
Masika et al (2021)(28)	Chamwino district, Tanzania	25 dementia (DSM-V) cases, 42 MCI, 19 controls		Kiswahili version of Montreal Cognitive Assessment (K-MoCA)		K-MoCA demonstrated acceptable reliability (Cronbach's alpha = 0.780). Concurrent validity was evident by its significant correlation with the IDEA screening test (Pearson's r = 0.651, p < 0.001).<br>Optimal cut-off score for MCI and dementia was 19 and 15, respectively, which yielded the sensitivity of 70% and specificity of 60% for MCI, and sensitivity of 72% and specificity of 60% for dementia.<br>Further analysis indicated that education and age influence performance on K-MoCA	
Paddick et al (2021)(29)	12 villages in Hai district, Kilimanjaro Tanzania	201 probable dementia, 85 possible dementia, 324 without dementia		Dementia screening mobile application consisting of 2 previously-validated culturally appropriate low-literacy screening tools for cognitive (IDEA cognitive screen) and functional impairment (abbreviated IDEA-IADL questionnaire).		For the IDEA cognitive screen, the area under the receiver operating characteristic (AUROC) curve was 0.79 (95% CI 0.74-0.83) for DSM-5 dementia diagnosis (sensitivity 84.8%, specificity 58.4%). For those 358 (44%) completing the full app, AUROC was 0.78 for combined cognitive and informant-reported functional assessment.	
Paddick et al (2017)(30)	Hai district of Northern Tanzania on the slopes of Mount Kilmanjaro, Tanzania	34 DSM-IV mild/moderate dementia cases (median age - 80.0 (IQR: 76.5-85.3))<br>32 controls (median age - 74.0 (IQR: 68.0-83.0))		Alzheimer's Disease Assessment Scale – Cognitive (ADAS-Cog)		Median ADAS-Cog scores were 28.75 (interquartile range (IQR), 22.96–35.54) in mild/moderate dementia and 12.75 (IQR 9.08– 16.16) in controls.<br>The area under the receiver operating characteristic curve (AUC) was 0.973 (95% confidence interval (CI) 0.936–1.00) for dementia.<br>Internal consistency was high (Cronbach's α 0.884) and inter-rater reliability was excellent (intra-class correlation coefficient 0.905, 95% CI 0.804–0.964).	
Stone et al (2018)(31)	4 rural villages in Hai district demographic surveillance site (DSS) in	36 DSM-IV dementia cases, 62 healthy controls		A 3-item IDEA-IADL		The questionnaire was deemed to be valid and enhanced screening performance in 2 villages (AUROC: 0.857 and 0.895) but detracted from the accuracy of the IDEA cognitive screen in the other 2 villages (AUROC: 0.591 and 0.639).	

	northern Tanzania, Tanzania					These differences appeared to be due to differences in interpretation of responses to questions by the assessors	
Njamnshi et al (2008)(32)	Yaoundé, Cameroon	204 (37.2) HIV-positive and 204 (37.1) HIV-negative subjects		The International HIV Dementia Scale (IHDS)		The HIV-positive subjects had a mean IHDS score of 10.87 ± 0.91, whereas the HIV-negative subjects scored 11.28 ± 0.56 (P < 0.00001).<br>The number of HIV patients with IHDS total score ≤10 (abnormal) was 43 (21.1%), whereas just 5 HIV-negative subjects (2.5%) had an IHDS score of ≤10, P = 5.0 × e−10.<br>In the psychomotor speed subtest, there was a significant difference between the HIV-positive and HIV-negative groups, P = 0.0051. Twenty-two HIV-positive subjects (10.8%) scored ≤ 3, whereas just 8 HIV-negative subjects (3.9%) scored ≤ 3.<br>In the memory recall subtest, there were significant differences between the 2 groups as well, P < 0.00001. Eighteen (8.8%) HIV-negative subjects scored ≤ 3, whereas 51 (25%) HIV-positive subjects scored ≤ 3. Whereas 91.2% of the HIV-negative subjects had a score ≥ 3.5, 75% of the HIV-positive subjects had a score ≥ 3.5.<br>There was no difference in the motor speed subtest between the 2 groups, P = 0.1797.	
Baiyewu et al (2005)(33)	Ibadan, Nigeria	88 (80.4) dementia cases<br>296 (79.0) with cognitive impairment no dementia (CIND)<br>340 (78.2) normal controls		Constructional Praxis test;<br>Stick Design test		Gender, age, and education affected performance on both tests.<br>The Stick Design test was more acceptable than Constructional Praxis as measured by the number of participants with total test failure (3.9% vs. 15.1%).<br>The Stick Design test was significantly more sensitive to cognitive impairment and dementia than the Constructional Praxis test.<br>The authors conclude that Stick Design is a reasonable test of visuoconstructional ability in older cohorts with very limited educational exposure and literacy.	
Vamcampfort et al (2019)(34)	**Ghana, South Africa**, China, India, Mexico, Russia	**32715 individuals across 6 countries, namely: Ghana (4201),**		MCI was ascertained based on the recommendations of the National Institute on Aging-Alzheimer's Association		Overall data shows that weak handgrip strength is a predictor of MCI [OR = 1.41 (1.23-1.61)]	

		**South Africa (3672)**, China (12815), India (6191), Mexico (2070), Russia (3766)				Countrywise analyses showed that weak handgrip strength is associated with MCI in all countries although statistical significance was reached only in **Ghana** and China	
Edjolo et al (2019)(35)	Central African Republic [Nola (rural) and Bangui (urban)]<br><br>Republic of Congo [Gamboma (rural) and Brazzaville (urban)]	301 study participants; 20% MCI, 27% dementia		Central African – Daily Functioning Interference (DFI)		area under curve = 0.878; 95% CI = 0.839–0.916; sensitivity = 0.96; specificity = 0.69; LR+ = 3.10; LR– = 0.06; and classification accuracy = 0.75<br><br>high reliability (Cronbach's alpha = 0.92).<br><br>The cutoff for detecting 96% of those with dementia was with a latent score ≥0.035 that corresponds to the LAUNDRY limitation	
Chen et al (2010)(36)	Rural population sample of Kikuyu Kenyans aged in Nyeri, Kenya	16 (76.4) dementia (DSM-IIIR and ICD-10) cases and 84 (69.7) controls		MMSE<br><br>The CSID was translated from English to Kikuyu and was then independently back translated to English for an accuracy check. A Swahili version of the CSID was also produced for occasional use as a reference. This was to obtain clarity of meaning of some Kikuyu terms derived from Swahili, which most informants also spoke as the second language<br><br>The two resulting scores of the CSID from each subject and informant relative, i.e.		apolipoprotein E ε4 allele frequencies were high (~30%) and not different between normal subjects and those with probable AD<br><br>When the cut-off points were selected for 100% sensitivities, the specificities of the DF scores were remarkably similar (93.75%) in the Kenyan sample.<br><br>Authors propose the adapted CSID can be utilised to detect dementia among East Africans.	

				cognitive and informant scores, were computed to derive the discriminant function (DF) score for each subject			

Supplementary Tables 7: Neuroimaging Studies in Africa							
**Study**	**Regions/Country/ population**	**Sample disease (mean age)**	**Biomarker** (type, markers)	**Cognitive measures**	**Brain measures**	**Biomarker results**	**Multimodal results**
Akinyemi et al (2014)(15)	Abeokuta and Ibadan, Nigeria	143 (60.4 years)) stroke survivors and 74 (58.8) healthy controls		CSID, MMSE and V-NB	Schelten's scale	Medial temporal lobe atrophy [OR = 2.25 (1.16-4.35)] independently associated with cognitive dysfunction	
Amod et al (2022)(16)	Durban, South Africa	81 (66) patients consisting of 53 (58) PD and 28 (61) Parkinsonian plus disorders (PPD) (including multiple systems atrophy (MSA), progressive supranuclear palsy (PSP), dementia with Lewy bodies (DLB) and corticobasal degeneration (CBD)			ROI analysis of patterns of glucose metabolism (18F-FDG PET)	**Using final clinical diagnosis as gold standard**<br>**PD**<br>• Sensitivity 91% (79-97)<br>• Specificity 89% (72-98)<br>• PPV 94% (84-99)<br>• NPV 83% (65-94)<br>• Agreement 90% (82-96)<br>**PSP**<br>• Sensitivity 100% (72–100)<br>• Specificity 100% (95–100)<br>• PPV 100%<br>• NPV 100%<br>• Agreement 100% (96-100)<br>**MSA**<br>• Sensitivity 57% (18–90)<br>• Specificity 97% (91–100)<br>• PPV 67% (31–90)<br>• NPV 96% (91–98)<br>• Agreement 94% (86–98)<br>**DLB**	

						• Sensitivity 100% (63–100)<br>• Specificity 99% (93–100)<br>• PPV 94% 89% (53–98)<br>• NPV 100%<br>• Agreement 99%<br>**CBD**<br>• Sensitivity 100% (15–100)<br>• Specificity 100% (95–100)<br>• PPV 100%<br>• NPV 100%<br>• Agreement 100% (96–100)<br>**PPS**<br>• Sensitivity 89% (72–98)<br>• Specificity 94% (84–99)<br>• PPV 89% (73–96)<br>• NPV 94% (85–98)<br>• Agreement 93% (85–97)	
Akinyemi et al (2015) (17)	Abeokuta and Ibadan, Nigeria	8 (68.3) cases of post-stroke dementia (PSD)<br><br>24 (62.8) (vascular CIND)<br><br>26 (54.9) normal controls		Community Screening Instrument for Dementia (CSID)—cognitive part;<br>The Mini-Mental State Examination (MMSE);<br>Vascular Neuropsychological Battery (V-NB)	Scheltens medial temporal lobe atrophy (MTA) visual rating scale;<br><br>Scheltens visual rating scale for white matter hyperintensities (WMH)	This study is unique in being the first in sub-Saharan Africa to examine neuroimaging correlates of cognitive impairment and suggests that MTA, which has often been interpreted as a signature of Alzheimer pathology, may have a vascular basis resulting from cerebral hypoperfusion.<br><br>In a two-step multivariate regression analysis, MTA ($p < 0.035$ and $p < 0.016$) was sustained as an independent statistical predictor of cognitive outcome.<br>OR (normal vs vCIND) = 2.02 (1.05-3.87); p = 0.035<br>OR (vCIND vs PSD) = 2.25 (1.16-4.35); p = 0.016<br><br>WMH was not associated with cognitive outcome	
Mworozi et al (2019)(18)	Sub-counties of rural Busukuma and peri-urban Nansana of Wakiso	40 (19%) mild MMSE, 72 (34.3%) moderate MMSE, 6 (2.9%) severe MMSE, 92		MMSE	Carotid artery plaque detected on ultrasound	The presence of plaque was associated with an abnormal cognitive function at both univariate and multivariate analysis with respective OR = 3.8 (95% CI = 1.90–7.54, p-value = 0.0001) and OR = 3.4 (95% CI = 1.38–8.15, p-value = 0.007)	

	district, Uganda	(43.8%) normal					

Supplementary Table 8: Genetic Studies in Africa							
**Study**	**Regions/Country/ population**	**Sample disease (mean age)**	**Biomarker** (type, markers)	**Cognitive measures**	**Brain measures**	**Biomarker results**	**Multimodal results**
Rizig et al (2023)(1)	Africa (data from IPDGC-Nigeria and GP2) and Admixed African populations (data from GP2 and 23andMe)	1488 cases of PD, 196430 controls	GBA1 locus (rs3115534-G)			OR=1.58 (95% CI: 1.37-1.80), p =2.397e-14<br>The risk locus is rare in non-African/African admixed populations. Present in 39% cases in the study.<br>This signal mediates PD risk via eQTL mechanisms<br>**Other identified risk factor:**<br>age at onset, BETA =-2.004, SE =0.57, P = 0.0005)	
Kacem et al (2022)(2)	Tunisia	197 ALS patients including 167 unrelated sporadic patients, 11 familial form (including 17 fALS patients) and 13 JALS forms(including one patient with familial JALS)<br>No healthy controls	mutations in, C9orf72, SOD1 (exons 1-5), TARDBP (exon 6), FUS (exons 5, 6, 13/14, and 15), ALS2 (exons 3, 10, 28)			The pathogenic variant TARDBP p.G294A mutation was reported among 18 patients.<br>Repeat expansion in C9orf72 was recorded in 9 patients.<br>2 unrelated patients carried a double mutation in both C9orf72 and TARDBP genes.<br>The p.Asp91Val mutation in SOD1 was identified among 4 cases in homozygous state including 3 sALS and 1 familial JALS with recessive inheritance.<br>No pathogenic variants in FUS were identified, nor ALS2 variants in JALS cases.	

						The most frequently mutated gene is TARDBP (9.4%), followed by C9orf72 (3.9%) and SOD1 (2.1%).<br><br>Study broadens the mutational spectrum in patients with ALS and defines for the first time the mutational frequency of the main ALS genes in patients of African ethnicity.	
Yonova-Doing et al (2012) (3)	Zambia	38 PD and 1 Parkinsonian-pramidal syndrome<br><br>No healthy controls	LRRK2, SNCA, Parkin, PINK1, and DJ-1			The LRRK2 p.Gly2019Ser mutation was not detected.<br><br>A novel LRRK2 missense variant (p.Ala1464Gly) of possible pathogenic role was found in one case.<br><br>Two heterozygous, likely disease-causing deletions of parkin (exon 2 and exon 4) were detected in an early-onset case.<br><br>Pathogenic mutations were not detected in SNCA, PINK1, or DJ-1.<br><br>There were several single nucleotide polymorphisms in the above-mentioned genes	
Okubadejo et al (2022)(4)	Nigeria	1100 PD cases and 1097 age-matched healthy controls.<br><br>Of the `PD cases, 121 cases with impaired cognition and 922 with normal cognition were analysed	APOE	Movement Disorder Society (MDS) Unified Parkinson's Disease Rating Scale (UPDR single item question on cognitive status (Part 1 item 1.1 of the instrument)		Homozygosity for ε4 conferred a two-fold increased risk for cognitive impairment in PD (Hazards ratio 2.09 (95% CI 1.13–3.89), p = 0.02), whereas the presence of at least one ε2 allele reduced the likelihood of cognitive impairment (HR 0.41 (95% CI 0.19–0.88), p = 0.023).<br><br>None of the 18 PD participants homozygous for ε2 had cognitive impairment.<br><br>The Odds ratios (95% CI) for the comparison between PD and controls for allele distribution were as follows: ε2: 0.97 (0.87–1.08), p = 0.56; ε3: 1.10 (0.97–1.24), p = 0.15; ε4: 0.94 (0.97–1.03), p = 0.17	

Hendrie et al (2014)(5)	Ibadan, Nigeria and Indianapolis, US	**2200 Yoruba in Ibadan, Nigeria**<br>173 (75.72) incident AD and 2027 (72.99) with normal cognition<br>**1871 African Americans in Indianapolis, US**<br>182 (77.47) incident AD* and 1689 (75.52) with normal cognition<br><br>*AD was diagnosed using the NINCDS/ADRDA criteria	APOE ε4			**In comparison with participants without APOE ε4 alleles**<br>**Yoruba**<br>● Homozygous APOE ε4 Hazard ratio (HR) = 2.95 (1.67-5.19); p = 0.0002<br>● Heterozygous APOE ε4 HR = 1.21 (0.88-1.67); p = 0.2362<br>**African Americans**<br>● Homozygous APOE ε4 HR = 4.12 (2.33-7.28); p < 0.0001<br>● Heterozygous APOE ε4 HR = 2.31 (1.70-3.14); p < 0.0001<br><br>After adjusting for covariates, one or two copies of the APOE ε4 allele were significant risk factors for incident AD (p < 0.0001) and cognitive decline in the African-American population (p < 0001). In the Yoruba, only homozygosity for APOE ε4 was a significant risk factor for AD (p = 0.0002) but not for cognitive decline (p = 0.2346), however, possession of an e4 allele was significant for both incident AD (p = 0.0489) and cognitive decline (p = 0.0425).	
Landoulsi et al (2018)(6)	Tunisia	172 (75.84) LOAD patients and 158 (74.27) control subjects	Exon 2 of TREM2; APOE ε4			ApoE ε4 allele was overrepresented in LOAD patients compared to the control group (36.9 vs. 16.1%; p < 0.05)<br><br>4 previously reported nonsynonymous variants (p.Asp39Glu, p.Arg62His, p.Thr96Lys, and p.Val126Gly) and 1 novel synonymous variant (p.Gln109Gln) were identified; none of which was significantly associated with the risk of Alzheimer's disease.<br>The rare TREM2 variant (p.Arg47His), which was considered to be a risk factor for	

						Alzheimer's disease in European descent populations, was not detected in this cohort.<br><br>The association analysis of all the identified TREM2 variants and AD was not statistically significant	
Rajabli et al (2022)(7)	Ibadan, Nigeria,<br>African American population,<br>Puerto Rican population,<br>non-Hispanic White	**Ibadan, Nigeria**<br>63 (83.5) cases, 648 (81.1) controls<br><br>**African American population**<br>1850 (78.6) cases, 4331 (75.9) controls<br><br>**Puerto Rican population**<br>273 (76.5) cases, 275 (75.4) controls<br><br>**non-Hispanic White**<br>8463 (75.9) cases, 11365 (77.5) controls<br><br>AD diagnosis using the National Institute of Neurological and Communicative Disorders and Stroke-Alzheimer's Disease and Related Disorders Association criteria for AD	Objective was to find genetic variants that lower the risk for ApoE ε4;<br><br>locus at 19q13.31 was found reduce ApoE ε4 risk for AD in African ancestry			Significant interaction between the ApoE ε4 allele and the SNP rs10423769_A allele, (β = -0.54,SE = 0.12,p-value = 7.50x10-6) in the discovery data set, this finding was replicated in Ibadan (β = -1.32,SE = 0.52,p-value = 1.15x10-2) and Puerto Rican (β = -1.27,SE = 0.64,p-value = 4.91x10-2) individuals.<br><br>The non-Hispanic Whites analyses showed an interaction trending in the "protective" direction but failing to pass a 0.05 significance threshold (β = -1.51,SE = 0.84,p-value = 7.26x10-2).<br><br>The presence of the rs10423769_A allele reduces the odds ratio for Alzheimer disease risk from 7.2 for ApoE ε4/ε4 carriers lacking the A allele to 2.1 for ApoE ε4/ε4 carriers with at least one A allele.<br><br>This protective haplotype has a frequency of 12% in the African ancestry, but only 0.003 in Europeans. This locus is at 19q13.31	

Haithem et al (2018)(8)	Tunisia	200 (71.86) dementia (DSM-IV and ICD-10) cases, 300 (65.69) controls	APOE, ACE I/D and PON1-L55M polymorphisms	Mini Mental State Examination, Frontal Assessment Battery, Geriatric Depression Scale, The ClockDrawing Test, Five-Word Test and Instrumental Activities of Daily Living scale		Carrying the ApoE ε4 allele seems to increase dementia risk by 4.32 fold (p=0.001). The risk associated with ACE I and PON1-L55M T alleles were lower (2.58 and 2.11 fold, p<0.001 and p=0.015 respectively). When combined in haplotypes, these polymorphisms showed a cumulative and synergetic effect. GTICC haplotype appears to be associated with 9-fold dementia risk (p<0.001), whereas AADTT seems to reduce dementia risk by 80% (p=0.003)	
Bouhouche et al (2015)(9)	outpatient clinic of six medical centers in the province of Rabat-Salé, Morocco	21 consecutive patients with Huntington's disease from 17 families	IT15 gene CAG expansion			Clinical symptoms were predominantly motor (19/21). Twelve patients had psychiatric and behavioral disorders, and 11 patients had cognitive disorders essentially of memory impairment. Analysis of genetic results showed that 5 patients had reduced penetrant (RP) alleles and 16 had fully penetrant (FP) alleles. The mean CAG repeat length in patients with RP alleles was 38.4 ± 0.54, and 45.37 ± 8.30 in FP alleles. The age of onset and the size of the CAG repeat length showed significant inverse correlation (p <0.001, r = −0.754)<br><br>Clinical and genetic data of Moroccan patients are similar to those of Caucasian populations previously reported in the literature.	
Kadmiri et al (2014)(10)	Casablanca, Morocco	17 sporadic AD cases and 8 family cases<br><br>No controls	Exons 16 and 17 of the APP gene	MMSE, BEC96, visual short-term or digital memory assessment, work memory assessment, language assessment test (DO80)	All 25 patients underwent neuroimaging (18 MRI, 7 CT)	All patients had hippocampal and parahippocampal atrophy accompanied by ventricular extension (in 62% of familial cases and 47% of sporadic cases)<br>*"Approximately half of our patients (48 %) have a score lower than 10 and are affected by severe dementia, while 28 % are affected by moderate severe dementia and 12 % are lightly to moderately insane."*<br><br>Identified seven novel frameshift mutations in exons 16 and 17 of the APP gene, of which five were identified in familial AD cases and two in sporadic AD cases	

						Only one novel splice mutation was detected in a family case	
Heckmann et al (2004)(11)	Large Xhosa family in South Africa with early-onset autosomal dominant PS1-linked familial AD	13 individuals including 4 affected and 9 unaffected family members	Presenilin 1 (PS1) gene	Xhosa translation of DECO;<br><br>MMSE performed in one subject	Axial CT brain scans on all 4 affected individuals;<br><br>One subject had a single MRI brain scan that only included axial and sagittal T1-weighted sequences.<br><br>Single-photon emission computed tomography imaging was performed on one subject	In all affected subjects, head CT imaging showed generalized cerebral atrophy, most marked in the medial temporal lobes and tempoparietal regions, with ex-vacuodilatation of the lateral ventricles. The severity of cerebral atrophy paralleled the degree of cognitive dysfunction. However, the posterior fossa appeared normal in all the subjects. A T1-weighted MRI of the proband was of poor quality due to movement artefact but essentially showed the same features as the CT image, with marked cerebral atrophy most prominent in the medial temporal lobe. Single-photon emission computed tomography in patient IV-13 showed markedly reduced cerebral perfusion in the left medial temporal region, right parietal region and both parieto-occipital regions.<br><br>Initial linkage-based analysis using known DNA markers suggested allele cosegregation with a locus on chromosome 14. Direct sequencing of the PS1 gene disclosed a novel I143M (ATT to ATG at nucleotide 677) mutation that lies in a cluster in the second transmembrane domain of the protein. Examination of the proband's brain at autopsy revealed severe AD pathology characterized by neuronal loss, abundant β amyloid (Aβ) neuritic plaques (Aβ42) and neurofibrillary degeneration extending into the brainstem. The phenotype of the I143M mutation was clearly associated with a high degree of neurofibrillary change compared with early-onset sporadic AD cases.	

Fekih-Mrissa et al (2017)(12)	Tunis, Tunisia	60 (75.18) AD and 120 (72.94) healthy controls	plasminogen activator inhibitor 1 (PAI-1)	MMSE, clock-drawing tests, the 5-word test, auditory verbal learning test, and Frontal Assessment Battery	medial temporal lobe and hippocampal volume reduction on CT and/or MRI	The results show a significantly increased risk of AD in carriers of the 4G/4G and 4G/5G genotypes versus the wild-type 5G/5G genotype (4G/4G: 28.33% in patients vs 10.0% in controls; P < 10−3; OR = 8.78; 4G/5G: 55.0% in patients vs 38.33% in controls; OR = 4.45; P < 10−3).<br><br>The 4G allele was also more frequently found in patients compared with controls; P < 10−3; OR = 3.07.<br><br>For all participants and by gender, homozygotic carriers (4G/4G) were at an increased risk of AD over heterozygotes and women were at an increased risk over their male genotype counterparts. The odds ratio for AD among 4G/4G carriers for any group was approximately twice that of heterozygotes in the same group.<br><br>Women homozygotes ranked highest for AD risk (OR = 20.8) and, in fact, women heterozygotes (OR = 9.03) ranked higher for risk than male homozygotes (OR = 6.12).	

Supplementary Table 9: Fluid Biomarker (Plsma/CSF) Studies in South-East Asia

Study	Regions/Country/	Sample disease (mean age)	Biomarker<br>(type, markers)	Cognitive measures	Brain measures	Biomarker results	Multimodal results

	population						
Kandimalla et al (2011)[1]	South Asia/ India/	51 AD, 69 NAD, 79 NC 49 HC	CSF A42, hTau, ubiquitin	MMSE	NA	low AB42 levels in AD compared to non-AD, NC, HC. Tau and ubiquitin levels were significantly high in AD compared to non-AD, NC, HC	AB42 and high Tau–ubiquitin levels were found in North Indian AD patients.
Mansoori et al (2010)[2]	South Asia/ India/ Indigenous	74 AD, 46 VaD, 113 HC	Blood/ Serum ApoE, interleukin-6 (IL-6)–174 G/C gene polymorphism along with serum IL-6 levels	BDR MMSE	NA	The allele ε 4 of the ApoE gene was found to be associated with AD and VaD patients	ApoE ε4 allele is an important genetic marker for AD and VaD. Presence of both ApoE ε4 and IL-6 C genes increase the OR of having AD and VaD
Kandimalla et al (2013)[3]	South Asia/ India/ Indigenous	44 AD, 63 NAD, 70 NC 46 HC	CSF ApoE, Total ApoE and ApoE4	MMSE	NA	No significant difference was observed in CSF ApoE concentrations between the patients with AD and the controls	CSF ApoE measurement does not offer any diagnostic advantage for AD
Sonali et al (2013)[4]	South Asia/ India/Indigenous	63 AD, 15 aMCI, 63 HC,	Serum BDNF	MMSE	Val66Met polymorphism	No significant difference was obtained between BDNF genotype and allele distribution between AD patients, aMCI versus controls	val66met polymorphism and BDNF serum level between the three groups and genotype did not significantly affect the serum BDNF level or age, Mini-Mental State Examination score in AD and aMCI.
Sarada S et al., (2021)[5]	South Asia/India/ Indigenous	26 AD<br>24 Controls	NfL levels using automated immunoassay platforms- single molecule array (SIMOA)	CDR, HMSE, NPI	NA	plasma NfL levels were significantly higher in AD subjects ($p < 0.0001$) in comparison to controls	Significant inverse correlation was observed between HMSE and plasma NfL levels
Sarada S et al., (2021)[6]	South Asia/India/ Indigenous	30 AD<br>28 Controls<br>10 FTD, 2 DLB, 5 VaD, 3 Mixed, 1 PD	Serum amyloid β42, phospho-Tau181, neurofilament L using Simoa HD-X analyzer	HMSE, CDR, NPI	NA	Plasma phospho-Tau181 levels are elevated in AD subjects to a much greater extent in comparison to NAD and healthy controls in the order of AD>NAD >HC<br>Plasma amyloid β42 levels are grossly indistinguishable among AD, NAD and HC groups and thus, singly may have limited scope as screening test for AD	Ratio of neurofilament L to amyloid β42 could achieve significance in discriminating AD from NAD neurodegenerative dementia
Vishnu et al., (2016)[7]	South Asia/ India/ Indigenous	11 MCI AD, 5 MCI VaD, 41 AD, 11 VaD, 16, CSF C, 19, PL	CSF biomarkers	MMSE	MRI brain, FDG-PET brain	AD and VaD had a significantly lower value of clusterin than the control no correlation of plasma clusterin with AD severity	The sensitivity and specificity of plasma clusterin was low for any significance for clinical use.

V.Y. Vishnu et al., (2017)[8]	South Asia/India	41 AD<br>16 MCI<br>11 VaD	CSF Biomarkers Aβ42 and total tau (T-tau), Plasma biomarkers (IL6, Fibrinogen, D dimer and CRP)	MMSE, AD assessment Scale (Cognitive), PGI (Postgraduate Institute) Memory scale, Verbal fluency	MRI Brain<br>PET/CT imaging	Plasm fibrinogen and d-dimer, VaD> AD	Plasma fibrinogen levels can be helpful in differentiating between VaD and AD
Lei D et al., (2023)[9]	South Asia/China	52 EOAD,<br>53 non-AD,<br>20 Controls	Ab42, total tau (t-tau), p-tau by ELISA	MoCA, MMSE, HADS	NA	Significant decrease in Ab42, and increase in t-tau, p-tau, t-tau/Ab42 and p-tau/Ab42, in the AD group compared with both the non-AD and control groups	t-tau/Ab42 and p-tau/Ab42 are more sensitive than relying on Ab42 levels alone
Gao F et al., (2023)[10]	South Asia/China	110 MCI, 208 AD, 55 Non-AD, 104 Healthy controls	Plasma Ab-40, Ab42, p-tau 181, t-Tau, NFL, GFAP	MMSE, CDR-SB, MoCA	18F-florbetapir PET and MRI	AD showed the highest levels of p-Tau and GFAP	Elevated levels of pTau and GFAP, but not NFL, were linked to a reduction in cortical thickness across various brain regions, particularly in the frontal, occipital, and temporal lobes
Tang CS et al., (2018)[11]	South Asia/Taiwan	27 Stroke without dementia, 34 VCI, 21 AD, 24 controls	Plasma Ab40, Ab42, tau using immunomagnetic reduction	CDR	NA	Plasma tau and Ab42 but not Ab40 were significantly higher in stroke patients than controls	Ab42 is a potential biomarker for dementia in stroke patients
Huang LC et al., (2022)[12]	South Asia/Taiwan	30 AD, 38 Normal adult children (AC), 21 Normal controls	Aβ1–42, Aβ1–40, total tau (T-tau) and phosphorylated tau (P-tau), APOE	AD8, MMSE, CDR	NA	Higher levels of Aβ1–40, P-tau, and P-tau/T-tau ratio, and lower levels of Aβ1–42 and Aβ1–42/Aβ1–40 ratio in the AD and AC groups. The correlation between the level of Aβ1–42 and Aβ1–40 and level of T-tau and P-tau was only observed in the AC but not in the AD group.	This suggests influence of FH for AD on a wide array of plasma biomarkers in the AC cohort of cognitively normal middle to older age individuals. Changes in the correlation between the levels of Aβ1–42 and Aβ1–40, and that of T-tau and P-tau in AC may be a biomarker for the development of AD.
Chou CT et al., (2016)[13]	South Asia/Taiwan	798 AD, 157 MCI, 401 controls	SNPs in SORL1, APOEe4, plasma Ab42, Ab40	MMSE, TMT A, Boston naming, verbal fluency category test, item word recall test	NA	SORL1 is a susceptible gene for LOAD and MCI. The SNP rs1784933 located in the 3′ region of the SORL1 genome and the nonsynonymous SNP rs2298813 were most significantly associated with AD and MCI.	Reduced plasma concentration of Aβ42 was found in individuals carrying the minor allele of the most significant SNP, rs1784933, implying a biological role of SORL1 genetic markers on the Aβ cascade.
Chen TB et al., (2019)[14]	South Asia/Taiwan	22 aMCI	Plasma Ab42 and total tau (t-tau), ApoE	MMSE, CDR, CVVLT	NA	Aβ42 and t-tau are associated with lower episodic verbal memory performance at baseline and cognitive decline over the course of follow-up	Aβ42 and t-tau are potential predictors for monitoring progressive cognitive decline in the MCI stage of AD

Hsu JL et al., (2017)[15]	South Asia/Taiwan	177 AD, 60 MCI, 108 Controls	Plasma Ab40, Ab42, clusterin	MMSE, CDR, NPI	MRI brain	Plasma clusterin level correlated with the severity of AD, plasma Aß1-42/Aß1-40 ratio correlated with the mean MTA score, and the clusterin level was correlated with the right-side PA score, in patients with AD	Different plasma biomarkers are linked individually to regional brain atrophy and that these changes were correspondingly associated with clinical symptoms in patients with AD
Chui MJ et al., (2014)[16]	South Asia/Taiwan	20 MCI, 10 AD, 20 Controls	Plasma tau	MMSE, CDR, GDS, TMT, WSCT,	PIB-PET, FDG-PET 3D MRI brain	Patients with MCI or early AD had significantly increased plasma tau levels compared with controls	Plasma tau levels were negatively associated with logical memory, visual reproduction, and verbal fluency; also negatively associated with volume of total gray matter, hippocampus, amygdala; and gray matter densities of various regions.
Yang CC et al., (2017)[17]	South Asia/Taiwan	29 MCI, 21 AD, 23 Controls	Plasma p-tau 181	MMSE, CDR, WEMS-III	MRI brain	Significant difference in the plasma p-tau181 concentration between healthy controls and patients with MCI	Plasma p-tau181 level correlated more to AD severity than plasma T-tau is.
Lin SY et al., (2019)[18]	South Asia/Taiwan	33 MCI, 19 Mild Dementia	APOE genotyping, and plasma Aβ40, Aβ42, and total tau protein	MMSE, GDS, WMS-III, CDR	Amyloid PET imaging	Amyloid PET+ participants had lower plasma Aβ42 levels than amyloid PET− subjects. APOE ε4 carriers had higher plasma Aβ1–42 than non-carriers.	APOE genotypes in combination with plasma Aβ42 levels can be used as a pre-screening tool for predicting positivity of amyloid PET findings in early dementia
HC Kuo et al., (2013)[19]	South Asia/Taiwan	9 AD, 9 MCI, 9 Controls	CSF Aβ42, F2-isoprostanes (F2-IsoPs) and F4-neuroprostanes (F4-NPs)	MMSE, CDR, ADAS-Cog, Visual association memory test	MRI brain	CSF F2-IsoPs and F4-NPs did not significantly differ among the three groups. Aβ42 in CSF was significantly higher in the control group compared with the mild AD group and a-MCI group	There was a significant positive correlation between the level of F2-IsoPs and Aβ42 in the a-MCI group and between the level of F2-IsoPs and F4-NPs in the mild AD group
Lin YT et al., (2009)[20]	South Asia/Taiwan	28 AD, 16 non-AD, 14 other neurological disorder (OND), 21 Controls,	CSF total tau and Ab42	MMSE, Cognitive Abilities Screening Instrument (CASI)	NA	Higher CSF total tau (t-tau) and lower amyloid-β42 levels in AD patients compared with those in HC and OND groups	CSF t-tau levels, but not Aβ42 levels had an inverse correlation with short-term memory for patients with AD
Thaweepoksomboon J et al., (2011)[21]	South Asia/Thailand	14 AD, 16 Non-AD, 5 VaD, 5 NPH, 4 FTLD	CSF Ab42, p tau-181, total tau	NA	NA	1 AD had increased CSF ptau-181, none had increased CSF total tau, 8 Non-AD had decreased CSF Ab42, 1 had increased CSF total tau and none had increased CSF p tau181[21]	The specificity of decreased level of CSF beta-amyloid (1-42) in AD against non-AD dementia was 50%. The specificity of increased CSF total tau and phosphorylated tau (ptau-181) protein in AD against non-AD dementia were 100% and 93.75% sequentially.
V Senanarong et al., (2012)[22]	South Asia/Thailand	14 AD, 10 Non-AD, 4 subjective memory complaint (SMC)	CSF Ab41, total tau, p-tau	Thai mental state examination (TMSE)	Brain MRI	AD had significantly lower levels of CSF Aβ42 than those with non-AD dementia and non- cases.	NA

Li WW et al., (2019)[23]	South Asia/ China	53 AD, 22 MCI, 9 Healthy controls	Plasma Ab42, Ab40, T-tau, APOE	MMSE, MoCA, CDR	MRI brain, PiB-PET and FDG-PET scans	Plasma Ab42 in AD subjects was lower than MCI subjects. 77.36% AD patients PiB-PET+, and 81.13% were FDG-PET+, 31.8% MCI among PiB-PET+ subjects and 22.7% among FDG-PET+ subjects.	PiB-PET+ subjects had lower value of Ab42/Ab40 ratio than PiB-PET– subjects. After adjustment for age, sex, and APOE4, the association between Ab42/Ab40 ratio and PiB-PET remained significant
Jia JP et al., (2005)[24]	South Asia/ China	39 AD, 38 VD, 35 controls	CSF t-tau, p-tau, Ab42, IL-1α, IL-1β, IL-2, IL6, TNFα	MMSE		T-tau in CSF was significantly higher in AD and VDthan that in controls, but there was no statistical difference between AD and VD groups. P-tau level in AD was remarkably higher than that in VD and controls. Aβ42 in AD was lower than that in VD and in controls but there was no significant difference between VD and controls. The level of IL-6 in CSF was higher in AD and VD than that in controls, CSF TNFα in AD was higher than that in controls	Combinative use of CSF biomarkers such as T-tau, P-tau, Aβ1–42, IL-6 and TNFα may improve diagnostic accuracy of AD. CSF T-tau and IL-6 are useful for screening AD and VD in certain population, while Aβ1–42 and TNFα have a potential to differentiate AD from VD.
Fang Y et al., (2019)[25]	South Asia/ China	86 MCI-AD, 92 MCI-MCI, 90 controls	APOE, serum BDNF levels,	MMSE, CDR, GDS	MRI Brain	Significant decrease in serum BDNF levels in both the MCI-AD and MCI-MCI patients possessing the APOE ε4 genotype compared to control group	Hippocampal volume in the APOE ε4 carriers of both the MCI-AD and MCI-MCI groups were reduced than those of control groups
Liu YH et al., (2014)[26]	South Asia/ China	110 AD, 120 Healthy controls	BDNF, ApoE	MMSE, CDR	NA	Serum BDNF levels were significantly lower in AD patients than that in NC, Serum BDNF levels were significantly lower in ApoE ε4+/− and ApoE ε4+/+ subjects compared with ApoE ε4−/− subjects.	ApoE ε4 carrier status is associated with reduced serum BDNF levels, and there were potential interactions between ApoE ε4 carrier status and serum BDNF levels on AD and MMSE scores
Wang T et al., (2014)[27]	South Asia/ China	97 AD, 122 normal Controls, 54 aMCI	Plasma IL-10, IL-6, Aβ40, Aβ42, phosphorylated tau 181, and total tau.	MMSE	MRI brain	Aβ40 is capable of distinguishing AD patients from the normal controls	The results do not provide evidence of the use of any plasma biomarker to discriminate between aMCI and NC groups.
Wei et al (2021)[28]	South Asia/ China	133 sMCI, 64 pMCI	CSF Biomarkers	MoCA	SUVR-MRI	pMCI individuals had higher mean 18F-florbetapir SUVR, CSF total-tau (t-tau), and p-tau181P than those in sMCI individuals<br>Significant differences in regions of interest of structural MRI between the two groups, including bilateral amygdala, hippocampus and entorhinal, bilateral inferior lateral ventricle, left superior and middle temporal, left posterior and caudal anterior cingulate	Specific CSF and cognitive measures that predict dementia progression in A+T+MCI might be<br>useful risk factors for assessing the risk of dementia progression
Zhang et al (2018)[29]	South Asia/ Singapore	52 HC, 22 sMCI, 47 pMCI, 18 AD	CSF biomarkers	MMSE<br>ADAS-cog scores	CSF SNAP-25 and SNAP-25/Aβ42	CSF SNAP-25 and SNAP-25/Aβ42 were increased in patients with pMCI and AD compared with CN, and in pMCI and AD compared with sMCI. Elevated SNAP-25/Aβ42 ratio was associated with the rate of hippocampal atrophy in pMCI and the rate of change of cognitive impairment in CN	Both CSF SNAP-25 and SNAP-25/Aβ42 ratio are already increased at the early clinical stage of AD, and indicate the promise of CSF SNAP-25 and SNAP-25/Aβ42 ratio as diagnostic and prognostic biomarkers for the earliest symptomatic stage of AD

Chua et al (2020)[30]	South Asia/ Singapore/	80 HC, 160 CinD, 113 AD, 31 VaD	Plasma biomarkers (Sphingosine-1-phosphates (S1Ps)	NA	NA	Only d16:1 S1P was significantly reduced in the plasma of VaD, but not AD, patients, while the d18:1 to d16:1 ratios were increased in all cognitive subgroups (CIND, AD, and VaD).	Plasma d16:1 S1P may be useful as a diagnostic marker for VaD, while the d18:1 to d16:1 S1P ratio is an index of dysregulated S1P-mediated immunomodulation leading to chronic inflammation-associated neurodegeneration and cerebrovascular damage
Chai et al (2021)[31]	South Asia/ Singapore/	80 NCI, 158 CIND, 140 D	Blood biomarkers Osteopontin (OPN)	MMSE MoCA	MRI	Increased OPN and VCI groups, namely CIND with CeVD, AD with CeVD and VaD.<br>Higher OPN was also significantly associated with AD even in the absence of CeVD.<br>OPN significantly associated with neuroimaging markers of CeVD and neurodegeneration, including cortical infarcts, lacunes, white matter hyperintensities and brain atrophy.	OPN may play a role in both VCI and neurodegenerative dementia
Tan et al (2021)[32]	South Asia/ Singapore	8 HC, 28 AD, 12 FTD	CSF biomarkers	MMSE MoCA		miR-320a, miR-328-3p, and miR-204-5p were significantly lower in AD versus controls. miR-320a was reduced in FTD versus controls and miR-328-3p was lower in AD versus FTD. Notably, lower miR-328-3p levels could differentiate AD from FTD and controls with an AUC and showed significant correlation with lower CSF Aβ42 levels	miR-320a and miR-204-5p as reliable biomarkers for AD and FTD and report miR-328-3p as a novel AD biomarker.
Yang et al (2012)[33]	South Asia/ Singapore	72 NC, 86 MCI-s, 25 MCI-c, 35 AD	CSF biomarkers Ab42, t-tau, and p-tau,	CDR MMSE	NA	CSF markers, including Ab42, t-tau, and p-tau, distinguished MCI or AD from NC, while the Ab42 CSF marker contributed to the differentiation between MCI and AD<br>The hippocampal shapes performed better than the hippocampal volumes in classifying NC and MCI, NC and AD, as well as MCI and AD	Volumetric information may be good for the early stage of AD, while morphological shapes should be considered as markers in the prediction of MCI conversion to AD together with the CSF markers
Zhu et al (2017)[34]	South Asia/ Singapore	96 AD, 140 CIND, 79 HC	Serum biomarkers	NA-	MRI (WMHs)	High IL-8, but not the other measured cytokines, were associated with both CIND and AD only in the presence of significant CeVD.<br>Only WMH was associated with higher IL-8 levels in CIND and AD	Serum IL-8 may have clinical utility as a biomarker for WMH in AD
Moon et al., (2021)[35]	South Asia/ Korea	29 CN, 58 SCD, 29 MCI, 23 AD	CSF biomarkers (Aβ42, Aβ40, total-tau, & phosphorylated-tau181)	MMSE SMCQ	amyloid PET	Aβ42, Aβ42/Aβ40, t-tau/Aβ42, p-tau/Aβ42 showed good agreement with Aβ-PET Cognitive and neuropsychological scores CN>SCD>MCI>AD.	The Korean-specific Aβ-PET-based CSF biomarker cutoffs measured by the Lumipulse assay strongly predicts progression of cognitive decline.

						CSF AD biomarkers measured by different immunoassay platforms show strong intercorrelated agreement with Aβ-PET in Koreans	
Park et al (2022)[36]	South Asia/ Korea	51 CN, 54 aMCI, 31 AD	plasma (NFL/A1–42) CSF ATN Biomarker	K-MMSE	b- Amyloid PET Imaging	With disease progression, the NFL concentrations increased, and A1–42 concentrations decreased. The plasma and CSF NFL/A1–42 were strongly correlated. Plasma NFL/A1–42 was strongly correlated with hippocampal volume/intracranial volume	plasma NFL/A1–42 as a non-invasive plasma-based biomarker for early diagnosis and monitoring of AD spectrum disease progression
Park et al (2019)[37]	South Asia/ Korea	87 HC, 56 aMCI, 34 AD	CSF Aβ1–42, total tau protein (t-au), tau protein phosphorylated at threonine 181 (p-Tau181)	K-MMSE	PET florbetaben	For the detection of prodromal AD in patients with aMCI, the cutoff values of CSF Aβ1–42, t-Tau, and p-Tau181 useful CSF biomarkers is well correlated with the stages of the AD spectrum.	CSF biomarkers are very useful tools for the differential diagnosis of prodromal AD in aMCI patients.
Nam et al (2020)[38]	South Asia/ Korea	26 HC, 30 MCI, 20 Mild-AD	serum tau proteins	MMSE	-	NEX t-tau and p-tau (S202) were significantly higher in the mild-AD group compared to AMC and MCI. Serum amyloid (A1–42) was lower in the mild-AD group compared to MCI	Serum tau proteins, especially NEX tau proteins, are useful biomarkers for monitoring AD progression.
Lee et al (2020)[39]	South Asia/ Korea	51 NC, 23 MCI, 65 AD	CSF biomarkers	-	amyloid PET	CSF Aβ42, total tau (t-tau) and phosphorylated tau (p-tau) significantly differed across the three groups. CSF biomarker abnormalities led to a majority of NC categorized into A-T-N-(73%), MCI as A+T-N-(30%)/A+T+N+(26%), and ADD as A+T+N+(57%).	CSF biomarkers had high sensitivity and specificity in differentiating ADD from NC and were as accurate as amyloid PET.
Lim et al (2020)[40]	South Asia/ Korea	86 HC, 20 Pre-AD, 56 Pro-AD, 49 ADD	CSF biomarkers	MMSE	-	AB1-42 levels decreased, while t-Tau and p-Tau levels increased according to the AD stages	CSF p-Tau highly accurate for distinguishing both preclinical AD and prodromal AD from HC.
Park et al (2019)[41]	South Asia/ Korea	52 CN, 9 MCI, 15 ADD	Plasma biomarkers	MMSE	FDG PET	Significant correlations of plasma p-tau, t-tau, p-tau/amyloid-b1–42, and t-tau/amyloidb1–42 with brain tau deposition t-tau/amyloid-b1–42 in plasma was highly predictive of brain tau deposition the brain regions where plasma t-tau/amyloid-b1–42 correlated with brain tau were similar to the typical deposition sites of neurofibrillary tangles in AD	Plasma tau and amyloid-b1–42 levels might be potential biomarkers for predicting brain tau pathology and neurodegeneration

Kang JH et al (2016)[1]	South Asia/ South Korea	660 PD, 189 controls	CSF α-synuclein, Aβ1-42, t-tau, and tau phosphorylated Thr181 (p-tau)	UPDRS	NA	CSF α-syn, t-tau and p-tau levels, but not Aβ1-42, were significantly lower in PD compared with HC, while the diagnostic value of the individual CSF biomarkers for PD diagnosis was limited due to large overlap.	Low level of CSF Aβ1-42 or the higher CSF t-tau/Aβ1-42 ratio was associated with more severe baseline cognition and motor symptoms of PD, a strong, significant correlation between the level of CSF α-syn and t-tau, less so for p-tau and Aβ1-42, and between Aβ1-42 and tau species in both HC and PD. APOE ε4 genotype was associated with low levels of CSF Aβ1-42 in both the HC and PD subjects, but not with diagnosis of PD or clinical features.
Chen Q et al., (2020)[2]	South Asia/China	151 PD, 21 MSA, 138 HCs	Plasma circulating miRNA expression	NA	NA	Elevated miR-133b and miR-221-3p discriminated early-stage PD from controls with 94.4% sensitivity and 91.1% specificity.	Elevated miR-133b and miR-221-3p potentially represent good biomarkers for early PD, and a combination of miR-133b, miR-221-3p and miR-4454 has the potential to serve as a non-invasive biomarker for PD diagnosis.
Sanyal J et al., (2021)[3]	South Asia/ India	530 PD	CSF and serum Ca, Mg, Iron	NA	NA	Metallomic profile of 110 CSF and 530 serum samples showed significant variations in 10 elements of CSF and six in serum of patients compared to controls.	The model in the study provides 99% accuracy as diagnostic biomarkers in detection of PD from CSF and serum
Khosla R et al., (2020)[4]	South Asia/ India	54 ALS, 32 controls	VEGF, VEGFR2, Optineurin, MCP-1, angiogenin and TDP-43	NA	NA	CSF levels of VEGF ($P = .014$) and ANG ($P = .009$) were decreased, whereas VEGFR2 was higher ($P = .002$) in patients with ALS than in controls. TDP-43 positively correlated with MCP-1 ($P = .003$), VEGF ($P < .001$), and VEGFR2 ($P < .001$) in patients with ALS.	
Varghese AM et al., (2013)[5]	South Asia/ India	31 ALS	Quantitative mass spectrometry of ALS-CSF chitotriosidase-1 (CHIT-1)	NA	NA	Elevated CHIT-1 levels in the ALS-CSF suggest a definitive role for the enzyme in the disease pathogenesis	NA

Thomas A et al., (2023)[6]	South Asia/ India	142 ALS	Serum and CSF VEGF level	NA	NA	CSF VEGF levels of ALS patients (46.18 ± 27.8) were significantly elevated compared to controls (25.95 ± 25.64 pg/ml) ($p = 0.001$), but not serum VEGF.	NA
Deng X et al., (2022)[7]	South Asia/ Singapore	205 PD, 102 non-PD	Serum total cholesterol (TC), triglyceride (TG), HDL-C, Apo A1, LDL-C, and apolipoprotein B (Apo B).	NA	NA	PD patients had significantly lower level of lipid panel including TC, TG, HDL-C, Apo A1, LDL-C, and Apo B (all $p < 0.05$).	TC, TG, Apo A1, and Apo B levels were independent protective factors ($p < 0.05$) for PD in the logistic regression model.
Seet RC et al., (2010)[8]	South Asia/ Singapore	61 PD, 61 HCs	Serum lipid and DNA oxidation products	NA	NA	Plasma F(2)-IsoPs, HETEs, 7beta-and 27-hydroxycholesterol, 7-ketocholesterol, F(4)-NPs, and urinary 8-OHdG were elevated, whereas the levels of plasma PLA(2) and PAF-AH activities were lower, in PD patients compared to controls ($p< 0.05$).	Oxidative damage markers are systemically elevated in PD, which may give clues about the relation of oxidative damage to the onset and progression of PD
Huang X et al., (2018)[9]	South Asia/ Singapore	125 PD	Fasting Serum Uric acid levels	three motor subtypes: tremor-dominant (TD), PIGD, and mixed	NA	The mean serum uric acid levels were significantly different between the three motor subtypes ($p = 0.0106$), with the mixed subtype having the lowest serum uric acid levels.	Higher serum uric acid levels were associated with TD motor subtype and less fatigue in early PD
Songsomboon C et al., (2020)[10]	South Asia/ Singapore	61 PD, 135 HCs	Serum Uric acid (UA), UA/creatinine ratio, Serum Bilirubin	UPDRS scale	NA	SUA/SCr ratio, but not SUA, was significantly lower in the PD patients than in the controls.	Serum total bilirubin (TB) and indirect bilirubin (IDB) were significantly higher in the PD patients. The SUA/SCr ratio is more sensitive than SUA in determining their association with PD.
Rathnayake D et al., (2019)[11]	South Asia/ Sri Lanka	72 PD, 56 HCs	serum immune mediators (IFNγ, TNFα, IL-10, and NOx)	NA	NA	Increase in serum IFNγ and IL-10 was observed in PD compared to healthy controls ($p < 0.001$). The Th1: Th2 (IFNγ: IL-10) cytokine ratio was higher in PD of 3–12 years compared with PD < 1 year ($p < 0.001$). A low serum NOx level was associated with cognitive impairment ($p = 0.002$)	The potential of using multi-biomarker panel, IFNγ, IL-10 and TNFα, for detection of PD onset was evident
Cao XY et al., (2017)[12]	South Asia/ China	109 PD, 40 HCs	Serum miRNAs using qRT-PCR technque	NA	NA	When compared with the control group, the area under the curve (AUC) values for miR-19b, miR-24, and miR-195 were 0.753, 0.908, and 0.697, respectively.	Analysis of the expression levels of miR-19b, miR-24, and miR-195 in serum may be useful for the diagnosis of PD.
Xiong M et al., (2021)[13]	South Asia/ China	40 PD, 35 HCs	Serum LCN2	NA	NA	Serum LCN2 levels were not significantly increased in PD patients compared with HC (4.9 [- 0.7 to 18.6] vs 1.9 [- 1.5 to 16.9] ng/mL, $P = 0.33$)	NA
Zhu Y et al., (2021)[14]	South Asia/ China	58 PD, 91 HCs	Serum SIRT1 determined by ELISA	NA	NA	Serum SIRT1 was significantly reduced in PD patients compared with controls.	SIRT1 may be a potential biomarker for PD.

Yifeng Li et al., (2023)[15]	South Asia/ China	165 vascular parkinsonism, 159 PD	Serum Sirtuin 1, and cytokines	MMSE UPDRS	NA	Serum SIRT1 levels were remarkably decreased in VP patients.SIRT1 levels were negatively correlated with levels of IL-6, TNF- α and hcy.	
Xu XM et al., (2018)[16]	South Asia/ China	77 PD, 39 HCs	Serum ProNGF	UPDRS	NA	Median concentration of proNGF was significantly lower (p = 0.000) in PD patients (94.91 ng/L, range 85.92-118.06 ng/L) compared with that of healthy controls (106.67 ng/L, range 102.39-122.06 ng/L).	proNGF concentration positively correlated with UPDRS. Serum proNGF concentration may represent a biomarker for PD and its role in the pathogenesis of PD thus warrants further investigation.
Ma LZ et al., (2021)[17]	South Asia/ China	301 PD, 144 HCs	Serum Nfl	Amyloid imaging	NA	PD patients had higher serum NfL than controls at baseline (p = 0.031), and NfL increase was faster in PD group (p < 0.001).	Baseline NfL of the third tertile of high concentrations was associated with a future high risk of PD dementia
Zheng H et al., (2022)[18]	South Asia/ China	100 PD, 100 HCs	Serum PRR14, VCAM-1, sCD163	NA	NA	PD exhibited increased PRR14 and VCAM-1 serum levels compared with HCs.	Bivariate correlation analysis revealed that there was a positive correlation between VCAM-1 and AAO
Liang H et al., (2022)[19]	South Asia/ China	53 PD, 49 HCs	Serum levels of sNogo-B and α-Synuclein (α-Syn)	UPDRS	NA	Serum sNogo-B level is significantly lower in the PD group than that in healthy controls and is negatively correlated with UPDRS-III score as well as serum α-Syn level	Decreased serum sNogo-B may be a potential biomarker for PD. Lower Nogo-B level reflects worse motor function and disease progression of PD.
Ma W et al., (2016)[20]	South Asia/ China	138 PD, 112 HCs	serum miRNA measured by RTPCR	UPDRS	NA	serums miR-29c, miR-146a, miR-214, and miR-221 were significantly decreased in PD patients compared with healthy control populations.	Serum miR-221 was positively correlated with UPDRS-III (r = .4702) and UPDRS-V (r = .4788) score in PD patients.
Cui SS et al., (2019)[21]	South Asia/ China	PD, ET , HCs	Serum sLAG-3 was measured by ELISA	NA	NA	Serum sLAG-3 levels in patients with PD were significantly higher than those in ET patients and age- and sex-matched controls.	Serum sLAG-3 was associated with non-motor symptoms and excessive daytime sleep.
Yi X et al., (2021)[22]	South Asia/ China	156 PD	Serum mBDNF and proBDNF levels measured by ELISA	NA	NA	Serum levels of mBDNF and mBDNF/proBDNF were significantly lower in the PD group (19.73 ± 7.31 and 0.09 ± 0.05 ng/ml) as compared with the non PD group (23.47 ± 8.21 and 0.15 ± 0.12 ng/ml) (p < 0.01 for both) and in the PD group (19.24 ± 7.20 and 0.09 ± 0.05 ng/ml) as compared with the non PD group	mBDNF/proBDNF can be used as biomarkers for early stage Parkinson's disease;
Wang X et al., (2023)[23]	South Asia/ China	1125 PD	Serum NfL	NA	NA	Significant association between serum NfL and early symptoms of PD.	There was a significant positive correlation between NfL and smell dysfunction, short sleep and long sleep.
Tian C et al., (2019)[24]	South Asia/ China	225 PD, 133 HCs	Serm Ser129 α-Syn,	NA	NA	Total and aggregated α-Syn levels were significantly higher in the membrane fraction of PD patients compared to healthy controls	NA

Chen D et al., (2023)[25]	South Asia/ China	244 MSA, 200 PD, 244 HCs	vitamin A, B1, B2, B9 (folate), B12, C, D, and E.	NA	NA	Compared with the healthy controls, decreased serum folate levels and increased serum vitamin A and C levels were detected in MSA patients. no differences detected between MSA and PD patients.	In MSA patients, significant correlation was found between vitamin A, folate, or vitamin C and relevant clinical scales or laboratory findings
Gao L et al., (2014)[26]	South Asia/ China	ALS	Serum and CSF VEGF levels	NA	NA	VEGF levels were found to increase significantly in CSF and serum in ALS patients studied; they were positively and significantly correlated with the disease duration in ALS patients and inversely and significantly correlated with disease progression rate (DPR) of ALS patients.	NA
Yang X et al., (2015)[27]	South Asia/ China	58 ALS, 45 HCs	Serum and CSF MIP-1α	ALSFRS	NA	MIP-1α in patients with ALS significantly increased compared to controls and they were positively correlated with duration.	MIP-1α showed negative correlations with disease progression rate and the decrease in ALSFRS-r
Shi J, et al., (2021)[28]	South Asia/ China	52 ALS, 30 controls	Serum and CSF NFL and phosphorylated neurofilament heavy chain (NFH)	ALSFRS	NA	Serum and CSF levels of NFL and pNFH in ALS patients were significantly increased. These values were negatively correlated with disease duration	Cox proportional hazards regressions confirmed that NFL and pNFH were significant predictors of survival. Overall, NFL and pNFH in serum and CSF can be used as reliable biomarkers in ALS.
Li R et al., (2020)[29]	South Asia/ China	20 ALS, 20 HCs	CSF biomarker, UCHL1	NA	NA	Elevated levels of CSF-derived UCHL1 in both discovery and validation cohorts	serum UCHL1 levels showed a positive relationship with the burden of UMN and LMN dysfunction
Liu J et al., (2015)[30]	South Asia/ China	52 ALS, 31 non-ALS patients	Levels of IFN-γ in CSF and serum were assessed	ALSFRS	NA	Compared to the non-ALS patients, the ALS patients displayed significantly increased levels of IFN-γ in both CSF and serum,	CSF IFN-γ was a more reliable biomarker of disease diagnosis and progression than serum IFN-γ.
Luo X et al., (2013)[31]	South Asia/ China	61 AD, 32 DLB, 40 HCs	CSF Visinin-like protein-1 (VILIP-1), Aβ1-42	NA	NA	CSF VILIP-1 level had significantly increased in AD patients compared with both normal controls and DLB patients.	he CSF VILIP-1 and VILIP-1/Aβ1-42 levels had enough diagnostic accuracy to allow the detection and differential diagnosis of AD.

Supplementary Table 10: Cognitive Studies in South-East Asia							
**Study**	**Regions/Country/Population**	**Sample disease (mean age)**	**Biomarker (type, markers)**	**Cognitive measures**	**Brain measures**	**Biomarker results**	**Multimodal results**
Shim et al (2017)	Asia/South Korea, India, Indonesia, Malaysia, Philippines, Singapore, Taiwan, Thailand/Indigenous	NA	Cognition/Functionality (MMSE)	MMSE - multilanguage	NA	Many items may be applicable or comparable with a little modification, for Asian countries. However, attention and calculation and repetition may be incomparable. There were some differences in the contents and the ways to administer.	
Jang et al (2023)	Asia/South Korea/Indigenous	108 HC (72.3)<br>12 MCI (75.8)	Cognition/Functionality (VARABOM, MoCA)	MoCA, VARABOM	NA	No difference in MoCA between HC and MCI<br>VR total score HC > MCI ($F = 8.674$, $p = 0.004$).<br><br>MoCA ~ VR scores in the total and matched subdomain scores.<br><br>The ROC curve analysis also showed a larger AUC for the VR test (0.765) than for the MOCA test (0.598), and the sensitivity and specificity of the VR program were 0.833 and 0.722, respectively.	

Kandiah et al (2014)	Asia/Singapore/Chinese, Malays, Indians	74 HC (61.9)<br>41 MCI (67.5)<br>91 AD (72.7)	Cognition/Functionality (VCAT)	MMSE, CDR, MoCA, VCAT	NA	AUC of VCAT for detection of cognitive impairment was found to be 93.3 (95% CI 90.1 to 96.4).<br><br>The Sensitivity and Specificity of VCAT for the diagnosis of cognitive impairment (MCI and mild AD) were 85.6% and 81.1%, respectively.<br><br>VCAT's diagnostic Sensitivity and Specificity comparable to those of the MoCA in the same cohort.<br><br>Mean time-to-complete VCAT was 15.7±7.3 min.	
Aniwattanapong et al (2019)	Asia/Thailand/Indigenous	60 HC (68.0)<br>60 aMCI (74.8)<br>60 AD (78.8)	Cognition/Functionality (MMSE, 10T BNT)	MMSE, CDR<br>CERAD-NP (15T-BNT, 60T-BNT, VFT, WLM)	NA	This study validated a 10-item T-BNT (10T-BNT), which yielded good internal consistency (0.92), a one-factor unidimensional structure, and adequate concurrent and discriminant validity.<br><br>Lower scores on the 10T-BNT highly significantly predict AD, but not aMCI, and are positively associated with VFT and WLM test scores.	Lowered 10T-BNT scores are significantly associated with the ApoE4 allele, lower folate levels and an increased triglyceride/HDL-cholesterol ratio.

Gurja et al (2022)	Asia/India/Indigenous	25 HC<br>28 MCI<br>26 AD	Cognition/Functionality (MMSE, CDR, E-Prime software)	MMSE, CDR<br>E-Prime software (working memory, semantic memory, attention)	NA	MCI > AD<br>MMSE, CDR, cognitive task scores<br><br>HC > MCI<br>Cognitive scores of all tasks for except MMSE and digits forward score.<br><br>ROC analysis showed that picture memory had 100% sensitivity, 91.6% specificity for AD and 88.4% sensitivity, 92.5% specificity for MCI.<br><br>Word memory had 92.3% specificity, 100% specificity for AD and 80.7% specificity, 84.6% specificity for MCI.	NA
Sjahrir et al (2001)	Asia/Indonesia/Indigenous	473 subjects	Cognition/Functionality (MMSE)	MMSE	NA	MMSE ~ age and education<br><br>median MMSE score - age group<br><20 - median 27<br>20~39 - median 28<br>40~49 - median 26<br>50~59 - median 27<br>>60 - median 21<br><br>median MMSE score - education level<br>>6 years education - median 24<br>7~12 - median 26<br>>12 - median 28	

Dong et al (2012)	Asia/Singapore/Indigenous	33 CU (62.8)<br>61 MCI (70.1)<br>136 Dementia (76.3)	Cognition/Functionality (MMSE, MoCA)	MMSE, MoCA, formal neuropsychological battery	NA	MoCA > MMSE (AUC) in discriminating md–MCI from the lower risk group for incident dementia (NCI and sd–MCI) [MoCA 0.92 (95% CI, 0.86–0.98) vs. MMSE 0.84 (95% CI, 0.75–0.92), p = 0.02).<br><br>At their optimal cut-off points, the MoCA (19/20) remained superior to the MMSE (23/24) in detecting md–MCI [sensitivity: 0.83 vs. 0.72; specificity: 0.86 vs. 0.83; PPV: 0.79 vs. 0.72; NPV: 0.89 vs. 0.83; correctly classified: 85.1% vs. 78.7%].	

Ng et al (2007)	Asia/Singapore/Chinese, Malays, Indians	1092 All (69)<br>501 Chinese<br>354 Malays<br>237 Indian	Cognition/Functionality (MMSE)	Mutilanguage MMSE	NA	Ethnic differences in mean MMSE scores among Chinese (26.2), Indians (25.0), and Malays (23.6), but only in noneducated subjects.<br><br>No ethnic differences in MMSE were observed in higher educated subjects.<br><br>Overall, MMSE discriminated well between subjects with and without dementia (cutoff: 23/24, area under the curve: 95%, sensitivity: 97.5%, specificity: 75.6%).<br><br>MMSE test performance was much better in higher educated subjects (higher specificity: 85.2%).<br>Lower specificities were shown in less educated subjects (57.3%), and in Malays (62.8%), and especially in less educated Malays (35.3%) and Indians (50.0%).	

Lim et al (2018)	Asia/Singapore, Malaysia, Indonesia,Philippines/Indigenous	164 HC (66.9)<br>120 CI (69.4)	Cognition/Functionality (VCAT)	MMSE, GDS, MoCA, multilanguage VCAT	NA	The VCAT, without local translation or adaptation, was effective in discriminating between HC and CI subjects<br><br>Mean (SD) VCAT scores for HC and CI subjects were 22.48 (3.50) and 14.17 (5.05) respectively.<br><br>AUC for MoCA (0.916, 95% CI 0.884–0.948) and the VCAT (0.905, 95% CI 0.870–0.940) in discriminating between HCs and CIs were comparable.<br><br>The multiple languages used to administer VCAT in four countries did not significantly influence test scores.	
Lee et al (2008)	Asia/South Korea/Indigenous	115 HC (69.1)<br>37 MCI (71.3)<br>44 AD (70.4)	Cognition/Functionality (MoCA-K)	MoCA-K, MMSE, CDR, CERAD	NA	MoCA-K scores were highly correlated with those of MMSE and CDR.<br><br>Using acutoff score of 22/23 MoCA-K sensitivity 89%, specificity 84% for screening MCI.<br><br>Internal consistency and test-retest reliability were good.	

Kang et al (1997)	Asia/South Korea/Indigenous	81 AD (67.9)<br>64 VaD (68.4)<br>23 PD (60.0)	Cognition/Functionality (K-MMSE)	K-MMSE<br>Blessed Orientation-Memory-Concentration Test	NA	K-MMSE sensitivity for detecting dementia - 70 ~ 83%<br><br>K-MMSE ~ Blessed Orientation-Memory-Information test (r=-0.78)	
Lim et al (2021)	Asia/South Korea/Indigenous	15 HC (69.1)<br>11 MCI (71.3)<br>12 Dementia (76.2)	Cognition/Functionality (13 experimental ADL tasks)	experimental ADL tasks based on IoT device<br>B-ADL, I-ADL<br>K-MMSE, CDR, GDS	NA	Signifcant diferences in the average success rate of 13 tasks were found among groups. success proportion<br>Dementia group (49.3%)<br>MCI group (78.3%)<br>HCl group (97.4%).<br><br>Correlation between classical ADL scales and the number of completed ADL tasks was statistically signifcant.<br><br>In particular, instrumental ADL (I-ADL) had stronger relationship with the number of completed ADL tasks than Barthel's ADL (B-ADL).<br><br>Dementia group required more time to accomplish the tasks when compared to MCI and HC groups.	

Tiwari et al (2009)	Asia/India/Indigenous	20 literate<br>20 illiterate	Cognition/Functionality (MMSE, HMSE)	Hindi version MMSE (HVMMSE)<br>HMSE	NA	All illiterate subjects scored below the cut-off on translated HVMMSE while only four of them scored below the cut-off on HMSE.<br><br>Among literate subjects, the translated HVMMSE and HMSE classified three subjects and one subject respectively as having possible cognitive impairment among urban elderlies.	
Ganguli et al (1995)	Asia/US-India/Indigenous	45 nondemented US<br>100 Indian rural random	Cognition/Functionality (MMSE)	MMSE, MoVIES battery	NA	Systematic, item-by-item, empirically based test development has shown that effective modifications can be made to existing tests that require reading and writing; and that culturally sensitive modifications can be made to render the test meaningful and relevant while still tapping the appropriate cognitive domains.<br><br>Certain cognitive functions, particularly orientation to time, remain difficult to test adequately in this type of population.<br><br>Educated individuals obtain higher test scores in both population	

Khobragade et al (2022)	Asia/India/Indigenous	4028 participants (LASI-DAD study)	Cognition/Functionality (IQCODE)	Informant Questionnaire on Cognitive Decline in the Elderly (IQCODE), HMSE, CERAD word list memory task	NA	Several IQCODE items had high levels of missingness, which was associated with urbanicity, respondent's gender, and informant's generation (same vs. younger generation).<br><br>Full IQCODE scores showed strong criterion validity against the HMSE; each 1-point increase in IQCODE score was associated with a 3.03-point lower score on the HMSE, controlling for age, gender, and urbanicity.<br><br>The statistically significant association between IQCODE and HMSE was stronger in urban than rural settings (p-value for interaction = 0.04).<br><br>Associations between IQCODE and HMSE remained unchanged after removing the three items with the highest levels of differential missingness	

Supplementary Table 11: Neuroimaging Studies in South-East Asia							
**Study**	**Regions/Country/Population**	**Sample disease (mean age)**	**Biomarker (type, markers)**	**Cognitive measures**	**Brain measures**	**Biomarker results**	
Chan et al (2011)	Asia/Singapore/ Chinese, non-Chinese	52 HC (64.7)<br>30 aMCI (74.5)<br>43 mild AD (76.6)	Neuroimage (CT or MRI)<br>Cognition/Functionality (CERAD profile)	MMSE, CDR, iADL, CERAD	MTA score, WMH score<br>Group comparison	late aMCI > HC<br>higher MTA scores<br><br>l-aMCI > either preMCI or e-aMCI<br>more progress to dementia over a mean follow-up period of 2.5 years and 20.0%, respectively).	NA
Huang et al (2013)	Asia/Taiwan/Indigenous	11 CU (69.3)<br>13 aMCI (70.0)<br>12 AD (75.8)	Neuroimage (MRI, 18F-Flobetapir PET)	MMSE, CDR, logical memory in the Wechsler memory scale-revised (LM), visual-association memory test (VAMT), category verbal fluency test (CVFT), trail-making A test (TMAT), clock-drawing test (CDT)	VBM<br>Group comparison	A+ rate - CU 9%, aMCI 62%, and AD 92% i<br><br>AD > CU<br>higher SUVRs in the global cortical, precuneus, frontal, parietal, occipital, temporal, and posterior cingulate areas;<br><br>aMCI > CU<br>higher values in the global cortical, precuneus, frontal, occipital and posterior cingulate areas.<br><br>Negative correlations of MMSE scores with SUVRs in the global cortical, precuneus, frontal, parietal, occipital, temporal, posterior cingulate and anterior cingulate areas	NA

Hong et al (2015)	Asia/South Korea/Indigenous	100 SCD-nc (64.8)<br>29 SCD-c (69.5)	Neuroimage (MRI)<br>Cognition/Functionality (New score system from Bootstrap model based on age, SVLT delayed recall, APOE4, MMSE recall)	SNSB, MMSE, CDR	WMH score	Older age, a lower MMSE recall score, APOE4 carrier, and a lower verbal delayed recall score were the most relevant predictors of progression, and a new modeling scale with these 4 predictors provided a better explanation of progression	NA
Mandal et al (2015)	Asia/India/Indigenous	21 HC (65.4), 28 HC (65.3)<br>22 MCI (66.8), 19 MCI (66.8)<br>21 AD (70.2), 19 AD (67.6)	Neuroimage (MRS - Glutathione)	MMSE, CDR, TMT A, TMT B-A	ROI based comparison	ROC analyses -<br>Hippocampal GSH<br>MCI and HC with 87.5% sensitivity,<br>100% specificity<br>Cortical GSH<br>MCI and AD with 91.7% sensitivity, 100% specificity	NA
Cho et al (2016)	Asia/South Korea/Indigenous	20 HC (71.5)15 MCI (72.9)20 AD (74.3)	Neuroimage (FBB PET, FTP PET, MRI)	SNSB, MMSE, CDR	VOI-based comparison	FTP binding was increased only in the entorhinal cortex in patients with MCI, while patients with AD exhibited greater binding in most cortical regions. In the patients with MCI and AD, FTP binding in most of the neocortex increased with a worsening of global cognitive function. The visual and verbal memory functions were associated with the extent of FTP binding, especially in the medial temporal regions. The 18F-FTP binding also correlated with the severity of regional atrophy of the cerebral cortex.	NA

Cho et al (2016)	Asia/South Korea/Indigenous	67 HC (66.1)<br>23 naMCI (68.5)<br>52 aMCI (70.3)<br>53 AD (74.9)	Neuroimage (FBB PET, FTP PET, MRI)	SNSB, MMSE, CDR	Surface-Based Comparison	Tau accumulation was most frequently observed in the medial temporal regions and spread stepwise to the basal and lateral temporal, inferior parietal, posterior cingulate, and other association cortices, and then ultimately to the primary cortical regions.<br><br>Amyloid accumulation was found with similar frequency in the diffuse neocortical areas and then finally spread to the medial temporal regions.<br><br>The image-based tau stage correlated with the general cognitive status, whereas cortical thinning was found only in the advanced tau stages: medial temporal region in stage V and widespread cortex in stage VI.	NA
Dhikav et al (2016)	Asia/India/Indigenous	32 CU (68.6)<br>40 AD (62.4)	Neuroimage (MRI)	MMSE	Hippocampal volume,<br>MTL rating scale scores	Hippocampal volume correlated with MTL score in both CU and AD groups.<br><br>Hippocampal volume had positive correlation with MMSE in AD.	NA

Hsu et al (2017)	Asia/Taiwan/Indigenous	160 HC (70.4)40 early AD (67.8)	Neuroimage (FDG-PET)Cognition (ECog)	MMSE, CDR,CEARD-NAB,Ecog	FDG SUVRAD t-sum score,	Significant correlation total ECog ~ CERAD-NAB (rho = -0.28, p < 0.01)Category verbal fluency test ~ executive domain of the ECog scale (rho =-0.20, p < 0.01). CERAD-NAB ~ AD-related hypometabolism (rho = -0.49, p < 0.01). Memory domain of the ECog scale ~ FDG uptake in the angular gyrus and posterior cingulum gyrus (rho = -0.41 and -0.46, P < 0.01).	
Cho et al (2017)	Asia/South Korea/Indigenous	15 Young HC (64.3)<br>10 EOMCI (63.2)<br>10 EOAD (64.8)<br>15 Old HC (76.5)<br>17 LOMCI (76.5)<br>21 LOAD (78.6)	Neuroimage (FBB PET, FTP PET, MRI)	SNSB, MMSE, CDR	VOI-based comparison	FTP binding<br>EOMCI = LOMCI<br>EOAD > LOAD greater binding in the parietooccipital cortex than LOAD.<br><br>The parieto-occipital FTP binding ~ visuospatial dysfunction in the EOAD spectrum,<br>The temporal cortex FTP ~ verbal memory dysfunction in the LOAD spectrum	

Vishnu et al (2017)	Asia/India/Indigenous	7 MCI-AD<br>4 MCI-VaSC<br>27 AD<br>8 VaD	Plasma (fibrinogen, D-dimer)<br>CSF (T-tau, Aβ42)<br>Neuroimage (FDG PET)	MMSE, ADAS-Cog, PGI memory scale, Verbal fluency – Controlled oral word test (phonemic) & Animal Names test, Quality of life - AD, ADCS-ADL	Visual assessment	Clinical-PET discordance was found in 7 patients.<br>One patient of MCI-VaSC had a normal PET study with elevated haemostatic biomarkers. Those with clinical diagnosis of AD either had normal hemostatic biomarkers and supporting AD profile CSF biomarkers where they were done.<br><br>The discordant vascular group had elevated plasma hemostatic biomarker with normal CSF profile.<br>Even those who were reported as FTD in PET imaging had Alzheimer profile and normal hemostatic factors.	NA
Balachandar et al (2017)	Asia/India/Indigenous	23 HC (69.9)<br>23 AD (70.2)	Neuroimage (rsfMRI)	NNB-E (NIMHANS neuropsychological battery for elderly)	Group comparison	Patients with AD having severe VS deficits exhibited significantly reduced rsFC in bilateral lingual gyri of the visual network compared to patients with mild VS deficits.	NA

Cho et al (2018)	Asia/South Korea/Indigenous	20 HC (63.4)20 bvFTD (63.0)20 AD (65.3)	Neuroimage (FBB PET, FTP PET, MRI)	SNSB, MMSE, CDR	VOI-based comparison	bvFTD > HC increased FTP binding in the putamen and globus pallidus bvFTD was associated with increased binding in the white matter regions underlying the frontal, anterior cingulate, and insula cortices.The bvFTD - predominantly subcortical FTP binding pattern	The clinical characteristics of bvFTD patients may be attributable tothe dysfunctional frontal-subcortical networks
Thientunyakit et al (2018)	Asia/Thailand/Indigenous	23 HC<br>32 AD (age not mentioned)	Neuroimage (18F-AV45, FDG)	NA	SUV	18F-AV45 uptake AD>HC<br>18F-FDG uptake AD<HC	NA
Suh et al (2019)	Asia/South Korea/Indigenous	159 mild AD (76.4)	Neuroimage (MRI)	MMSE, CDR	Visual assessment (Posterior atrophy, medial temporal atrophy)	Visual assessment of the MRI scans revealed that 112 patients (70.4%) showed MTA, whereas 80 patients (50.3%) showed PA.<br><br>The ORs with 95% confidence intervals for MTA and PA were 1.825 (0.819-4.070) and 2.844 (1.378- 5.835), respectively.<br><br>The association of visually assessed PA, but not MTA, with rapid progression was significant after adjustment for covariates.	

Hong et al (2019)	Asia/South Korea/Indigeno us	31 SCD<br>[23 A-SCD (67.8)<br>8 A+SCD (73.0)]`	Neuroimage (MRI, FBB PET)	SNSB, MMSE, CDR<br>Self-report questionnaire (CFQ)	WMH score	A total of 31 participants with SCD completed the study and 25.8% showed positive amyloid depositions.<br><br>The degree of periventricular WMH and hippocampal atrophy were more severe in A+ SCDs compared to the A- group.<br><br>In the self-reported questionnaire, the 'informant's report a decline' and 'symptom's onset after 65 years of age' were associated with more AD pathologic changes.	

Cho et al (2019)	Asia/South Korea/Indigenous	272 AD spectrum [195 ε4- (70.4)77 ε4+ (70.0)]	Neuroimage (FBB PET, FTP PET, MRI)	SNSB, MMSE, CDR	Region-wise multiple comparisons	In contrast to no change in global cortical FTP SUVR in the CU- and CU+ groups during the 2y period, global cortical FTP SUVR increased by 0.06 (2.9%) in the MCI+ group and 0.19 (8.0%) in the DEM+ group at follow-up. A+MCI group was associated with additional tau accumulation predominantly in the medial and inferior temporal corticesA+AD group showed increases in the lateral temporal cortex. Progressive tau accumulation occurred in the diffuse cortical areas in the MCI+ patients who developed dementia and the DEM+ patients who showed deterioration of global cognition, whereas there was only a small increase of additional tau accumulation in the lateral temporal cortex in those who did not show worsening of cognition. Deterioration of global cognition and language functions was associated with progression of diffuse tau accumulation in the association neocortex.	
Tripathi et al (2019)	Asia/India/Indigenous	87 aMCI (66.6)	Neuroimage (FDG PET)	MMSE	VBM	MCI convertor vs. non-convertor<br>FDG PET AUC 91.9%<br>Sensitivity 86.9%<br>Specificity 93.7%	NA

Mukku et al (2019)	Asia/India/Indigenous	14 EOAD<br>2 LOAD<br>5 MCI<br>(all participants 61.2)	Neuroimage (FDG PET)	NA	Visual assessment	Based on the pattern of hypometabolism, the MCI group had one patient each indicative of AD, Semantic-Frontotemporal dementia (Semantic-FTD), mixed Alzheimer's dementia (AD + FTD) and two patients had patterns suggestive of Behaviour Variant of FTD (Bv-FTD).<br><br>In Dementia group the pattern of hypometabolism was indicative of Bv-FTD<br>in seven, AD in four, PCA in one, Semantic-FTD in one, Mixed AD-LBD in one and no specific pattern in two patients. MRI and 18 F-FDG-PET brain had concordance in 9 (56.26%) patients	NA
Kumar et al (2019)	Asia/India/Indigenous	50 HC50 MCI	Plasma (SOD, GPX, MDA)Neuroimage (MRI-DTI)	MMSE	ROI based comparison	DTI metrics FA values in right and left frontal lobe, fornix, corpus callosum, while ADC values in right temporal lobe, hippocampus head, corpus callosum right, and forcep majorwere significantly altered in MCI as compared with controls. SOD and GPX levels were lower while MDA was increased in patients with MCI as compared with controls.	NA

Wong et al (2020)	Asia/Singapore/ Chinese, Malays, Indians	792 CI [262 Chinese (70.3) 276 Malays (70.9) 254 Indians (68.7)]	Neuroimage (MRI)	MMSE, MoCA, native language NP battery Executive function using the Frontal Assessment Battery and Maze Task Attention using the Digit Span, Visual Memory Span and Auditory Detection Language using the Boston Naming Test and Verbal Fluency Test Visuomotor speed using the Symbol Digit Modality Test and Digit Cancellation Test Visuoconstru ction using the Wechsler Memory Scale–Revised, Visual Reproduction Copy task, Clock	Cortical thickness, Subcortical volumes WMH volume	Malays > Chinese higher burden of intracranial stenosis (OR: 2.28. 95%CI: 1.23–4.20) and cortical atrophy (β: -0.60. 95%CI: -0.78, -0.41) Indians > Chinise higher burden of subcortical atrophy (β: -0.23. 95%CI: -0.40, -0.06).<br><br>Malays > Chinese cognitively impaired (OR: 3.79. 95%CI: 2.29–6.26) worse performance in global cognition (β: -0.51. 95%CI: -0.66, -0.37) Indians > Chinise cognitively impaired (OR: 2.87. 95%CI: 1.74–4.74) worse performance in global cognition (β: -0.32. 95%CI: -0.47, -0.17).	

				Drawing, and Wechsler Adult Intelligence Scale–Revised subtest of Block Design Verbal Memory using the Word List Recall and Story Recall Visual Memory using the Picture Recall and Wechsler Memory Scale–Revised Visual Reproduction Tests			

Ye et al (2020)	Asia/South Korea/Indigenous	21 HC (71.8)38 A+DLB (73.6)25 A-DLB (76.3)38 AD (70.8)	Neuroimage (FP-CIT PET, FDG PET, and FBB PET)	SNSB, MMSE	Group comparison	A-DLB, A+DLB > ADincreased metabolism in the bilateral central cerebellum, posterior putamen, and somatomotor corticesDLB and AD > HChypometabolism in the bilateral lateral temporal, temporo-parietal junction, posterior cingulate, and precuneus cortices. A-DLB, A+DLB > HChypometabolism in the bilateral thalami and dorsolateral prefrontal cortices, AD > HChypometabolism in the bilateral entorhinal cortices and hippocampi.	
Son et al (2020)	Asia/South Korea/Indigenous	5 HC<br>35 CI<br>(all subjects 68.5)	Neuroimage (FDG PET, dual phase FBB PET, MRI)	MMSE, CDR	SUVR<br>Visual assessment	ROI based SUVR<br>E-FBB ~ FDG<br>significant correlations ($P < 0.0001$)<br>FDG > E-FBB<br>higher significancy<br><br>Visually rated scores<br>E-FBB ~ FDG<br>significant correlation ($P<0.0001$)	

Park et al (2020)	Asia/South Korea/Indigenous	10 MCI (72.3)<br>10 AD (73.5)	Neuroimage (PiB PET, FC119S PET)	SNSB, MMSE, CDR	AD-signiture area SUVR	Except attention domain, all other cognitive domains were relatively impaired in AD compared with MCI.<br><br>Copy score of RCFT in MCI groups ~ frontal area in both 11C-PiB-PET and 18F-FC119S PET.<br><br>In AD group, 18F-FC119S PET presented more extensive correlation in each cognitive domain with multiple cortical areas compared with 11C-PiB-PET.	
Baek et al (2020)	Asia/South Korea/Indigenous	57 A-CU (65.9)7<br>A+CU (71.7)39<br>A+MCI (73.9)29<br>A+AD (72.7)	Neuroimage (FBB PET, FTP PET, MRI)	SNSB, MMSE, CDR	Trajectory - restricted cubic spline	Tau burden first emerged in the Braak's stage I–II regions, followed by stage III–IV regions, and finally in the stage V–VI regions. Time intervals between two time points at which Z-score curves rose above 2 were 17.3 years for the stages I–II and III–IV and 15.2 years for the stages III–IV and V–VI. Rise in the tau curve for stages I–II preceded that for global cortical Aβ, while the rise in global cortical Aβ curve preceded that for global cortical tau. Aβ accumulation rate was attenuated during the surge in tau burden in the global cortex and reached a plateau.	

Soman et al (2020)	Asia/India/Indigenous	31 HC (65.1)<br>30 MCI (65.7)<br>30 AD (67.2)	Neuroimage (rsfMRI)	ACE-M, CDR, RAVLT	seed analysis	Seed-based analysis between AD and controls revealed reduced posterior connectivity within the DMN, DAN and antero-posterior connectivity with SMN networks.<br><br>Reduced cerebellar connectivity of DMN and posterior connectivity within the FPN separated AD from MCI.<br><br>MCI-control comparisons revealed differences only on ICA.<br><br>Positive correlation was observed between FC in DMN network clusters with verbal list-learning (r = 0.50) and recall scores (r = 0.51) in AD, the latter additionally demonstrating correlation with SMN clusters (r = 0.50).	NA
Chong et al (2021)	Asia/Singapore/ Indigenous	43 HC (74)<br>99 MCI (76)<br>44 AD (77)<br>22 VaD (75)	Plasma (Aβ42/40, p-tau181, t-tau)<br>Neuroimage (MRI, PiB-PET or 18F-Flutafuranol PET)	MMSE	Hippocampal volume, WMH volume, number of lacune, SUVR	P-tau181/Aβ42 ratio showed the highest AUC for Aβ+ (AUC = 0.889) and for discriminating between AD Aβ+ and VaD Aβ- subjects (AUC = 0.903).<br><br>P-tau181/Aβ42 ratio was associated with hippocampal atrophy.<br><br>None of the biomarkers was associated with CeVD.	

Yoon et al (2021)	Asia/South Korea/Indigenous	12 A-CU (71)32 A+MCI (73)16 A+AD (71)	Neuroimage (FBB PET, dual phase)	SNSB, MMSE, CDR	dual phase PETVBM	Both the R1 and eFBB perfusion reductions in the cortical regions were significantly reduced from the Aβ+MCI to Aβ+AD groups in regional and voxel-wise analyses.Cortical Aβ depositions on dFBB Aβ+MCI = Aβ+AD There were strong positive correlations between the R1 and eFBB images in regional and voxel-wise analyses. Both perfusion components showed significant correlations with general and specific cognitive profiles.	
Hong et al (2021)	Asia/South Korea/Indigenous	35 A-SCD (67.1)<br>12 A+SCD (74.2)	Neuroimage (MRI, FBB PET)	SNSB, MMSE	EC volume, Hippocampal volume, PET SUVR	A+SCD > A-SCD<br>greater decline in the verbal memory function<br><br>MMSE scores decreased more in the A+SCD (1.1 in the A+SCD; 0.55 in the A-SCD), although it was not statistically significant.<br><br>Amyloid burden and baseline memory score were associated with memory decline.	

Roh et al (2021)	Asia/South Korea/Indigenous	168 SCD (70.9)<br>534 MCI (72.6)<br>211 AD (74.1)<br>80 VD (75.1)<br>20 Others (68.9)	Genetic (SNP)<br>Plasma (dermal fibroblast)<br>Neuroimage (MRI, FMM PET)<br>Cognition/Functionality (actigraphy)	SNSB, MMSE, GDS	Group comparison	Each group exhibited many differences in various clinical, neuropsychological, and neuroimaging results at baseline.<br><br>Baseline characteristics of BICWALZS participants in the MCI, AD, and VaD groups were generally acceptable and consistent with 26 worldwide dementia cohorts and another independent AD cohort in Korea.	
Mangalore et al (2021)	Asia/India/Indigenous	24 AD<br>7 PCA<br>17 bvFTD<br>11 svPPA<br>4 nfvPPA<br>5 PDD<br>3 PSP<br>3 CBD	Neuroimage (MRI)	NA	Visual assessment	AD and PCA-AD had predominant atrophy of splenium of CC.<br><br>In Bv-FTD, the genu and anterior half of the body of CC was atrophied, whereas in PNFA, PSP, PDD, and CBD there was atrophy of the body of CC giving a dumbbell like profile.	NA

Zhou et al (2022)	Asia/Taiwan/Indigenous	54 HC (80.0)42 MCI (81.3)	Neuroimage (MRI)	MMSE, CDR	DTI, rsfMRImechine learning classification	The mean diffusivity of hippocampus-temporal and thalamus-related fibers are significantly higher in MCI and could be used to classify 2 groups effectively. Compared with normal fibers, the degenerated fibers detected by the DTI indexes, especially for hippocampus-temporal fibers, have shown significantly higher correlations with cognitive scores. Compared with the hippocampus-temporal fibers, thalamus-related fibers have shownsignificantly higher correlations with depression scores within MCI.	
Sohn et al (2022)	Asia/South Korea/Indigenous	195 CU (71.7)<br>65 MCI (74.1)	Neuroimage (PiB PET, FDG PET, MRI) Cognition/Functionality (LTPAQ, MET, 39-item expanded version of the lifetime cognitive activity scale)	MMSE, CDR, CERAD-K	AD-specific area cortical metabolism, cortical thickness, amyloid PET SUVR	Physical activity (PA) of neither midlife nor late-life showed direct correspondence with any neuroimaging biomarker.<br><br>However, late-life PA moderated the relationship of brain Aβ deposition with AD-cortical metabolism (CM) and AD-cortical thickness (CT).<br><br>Aβ positivity had a significant negative effect on both AD-CM and AD-CT in individuals with lower late-life PA, but those with higher late-life PA did not show such results.<br><br>Midlife PA did not have such a moderation effect.	

Chakraborty et al (2022)	Asia/India/Indigenous	36 AD (65.7)	Neuroimage (MRI)	CDR	global cortical atrophy (GCA) score<br>Mesial temporal atrophy (MTA)score<br>Fazekas grading scale<br>Posterior parietal atrophy score	CDR worsening had correlation with low hippocampal volume, and high GCA, MTA, and Koedam's score	NA
Chong et al (2023)	Asia/Singapore/Indigenous	43 HC (74)<br>99 MCI (76)<br>44 AD (77)<br>22 VaD (75)	Plasma (NfL)<br>Neuroimage (MRI, PiB-PET or 18F-Flutafuranol PET)	NA	WMH, MTA score	N+WMH- or N-WMH+ manifested increased plasma NfL levels.<br><br>N+WMH+ showed the highest NfL compared to N+WMH-, N-WMH+, and N-WMH individuals.	
Chun et al (2023)	Asia/South Korea/Indigenous	205 Dementia (74.1)	Neuroimage (MRI, FBB, FMM, FTP PET)	SNSB, MMSE, CDR	Group comparison	In A+ category, compared with the A+T-, the frequency of A+T+ was significantly lower in V+ group (31.8%) than in V- group (64.4%) (p=0.004). Each AT(N) biomarker was predictive of cognitive decline in the V+ group as well as in the V- group (p<0.001). The V+ group showed more severe cognitive trajectories than the V- group in the A–T+, A–N+; p=0.002) and Alzheimer’s pathological changes (p<0.001) categories.	

Kim et al (2023)	Asia/South Korea/Indigenous	160 A- SCD (71.3)<br>36 A+SCD (72.0)<br>61 A-MCI (71.5)<br>54 A+MCI (74.5)	Neuroimage (QEEG, FBB PET)	NA	machine learning<br>group comparison	The best model showed 90.9% sensitivity, 76.7% specificity and 82.9% accuracy in MCI + SCD (33 Aβ +, 43 Aβ -).<br><br>Limited to SCD, 92.3% sensitivity, 75.0% specificity, 81.1% accuracy (13 Aβ +, 24 Aβ-). 90% sensitivity, 78.9% specificity and 84.6% accuracy for MCI (20 Aβ +, 19 Aβ -).<br><br>Aβ + and Aβ -, MCI and SCD<br>similar trends<br>enhancement of frontal/ frontotemporal theta; attenuation of mid-beta in centroparietal areas.	

Aishah et al (2023)	Asia/Malaysia/Indigenous	14 HC (65.9)<br>3 mild AD (73.7)<br>11 modeate AD (77.0)<br>2 severe AD (77.5)	Neuroimage (FDG PET)	MoCA, ADA-Cog	VBM	The atrophy rate within each ROI is significantly different between groups (c2=35.9021, df=3, p<0.0001), Wilcoxon method test showed statistically significant differences were observed between Moderate vs. Mild AD (p<0.0001), Moderate AD vs. healthy control (p=0.0005), Mild AD vs. HC (p=0.0372) and Severe AD vs. Moderate AD (p<0.0001).<br><br>The highest atrophy rate within each ROI between the median values ranked as follows severe AD vs. HC (p<0.0001) > mild AD vs. HC (p=0.0091) > severe AD vs. moderate AD (p=0.0143).	
Sheelakumari et al (2017)	Asia/India/Indigenous	20 HC (61.1)27 bvFTD (61.2)12 PPA (64.6)	Neuroimage (MRI)	Frontal Systems Behavior Scale (FrSBe)	Fractal dimension	bvFTD>HCsignificant reduction in FD values of skeleton and general structure PPAmore significant decrease in FD was noted in the whole brain and left hemisphere skeleton along with left hemisphere general structure.Only the right hemisphere skeleton had a significant correlation with total score of Frontal Systems Behavior Scale (FrSBe).	

Chung et al (2022)	Asia/South Korea/Indigenous	75 PDMCI (38 PDD-high risk, 37 PDD low-risk)	Neuroimage (WM connectivity)	SNSB, MMSE	Voxel wised tract-based spatitial statistics<br>- graph-theoretical concept to identify the subnetworks of WM connectivity	PDD-H < PDD-L<br>poorer cognitive performance on frontal/executive, visual memory/visuospatial, and attention/working memory/language function at baseline assessment.<br><br>PDD-H - more severely disrupted WM connectivity in both frontal and posterior cortical regions with 8 hub nodes in the degree-based statistic analysis.<br><br>The strength of structural connectivity within the identified subnetworks was correlated with the composite scores of frontal/executive function domain ($\gamma = 0.393$) and the risk score of PDD conversion within 5 years ($\gamma =- 0.480$).	NA
Baek et al (2020)	Asia/South Korea/Indigenous	96 ALS (probable or definite)<br>47 HC	Neuroimage (DTI)	NA	Voxel wise analysis for FA, AD, RD, MD, MO	ALS > HC had significant differences in DTI scalars in the diffuse tracts of the brain, which was predominant in the corticospinal tract at the brainstem and cerebellar peduncle area.<br><br>DTI ~ ALSFRS-R scores<br>delta ALSFRS-R score ~ disease progression.<br><br>More severe and widespread brain degeneration was observed in rapidly progressive ALS.	NA

Lee et al (2017)	Asia/South Korea/Indigenous	26 ALS<br>26 CVD<br>26 HC	Neuroimage (QSM)	NA	ROI based susceptability value - relative susceptibility	ALS > CVD, ALS > HC<br>cortical and relative susceptability value ($p = 0.01$, $p = 0.004$, $p < 0.001$, and $p < 0.001$, respectively).<br><br>ALS<CVD, ALS<HC<br>subcortical white matter mean ($p = 0.04$).<br><br>ROC curve - RSmean was 0.70	NA
Ahn et al (2019)	Asia/South Korea/Indigenous	27 PSP-RS<br>27 IPD<br>27 HC	Neuroimage (MRI)	NA	midbrain area, pons area, P/M ratio	PSP-RS < PD and HC<br>midbrain area ((midbrain, Pre-PSP-RS vs. PD = 1.01 cm2 vs. 1.29 cm2, $p < 0.001$, Pre-PSP-RS vs. controls = 1.01 cm2 vs. 1.29 cm2, $p < 0.001$)<br><br>PSP-RS > PD and HC<br>P/M ratio (Pre-PSP-RS vs. PD = 5.27 vs. 4.03, $p < 0.001$, Pre-PSP-RS vs. controls = 5.27 cm2 vs. 4.06 cm2, $p < 0.001$)<br><br>The P/M ratio had high sensitivity (vs. PD, 96.3%, vs. control, 88.9%) and specificity (vs. PD, 81.5%, vs. control, 96.3%) in differentiating Pre-PSP-RS patients from PD and control subjects.	NA

Yoo et al (2022)	Asia/South Korea/Indigenous	55 DLB (15 prodromal, 30 probable)<br>13 HC	Neuroimage (MRI, FP-CIT, FDG, FBB PET)	SNSB, MMSE, CDR	MRI, FP-CIT, FDG, FBB PET	Independent of amyloid deposition, caudate and putamen DAT availabilities were positively correlated with brain metabolism in the DLB-specific hypometabolic regions, most prominently in the occipital and lateral parietal cortices.<br><br>Both reduced caudate dopamine and brain hypometabolism were associated with low z-scores of RCFT copy, SVLT immediate recall and COWAT–animal.<br><br>Path analyses showed that the effect of reduced caudate dopamine on the RCFT copy z-score was completely mediated by brain hypometabolism, whereas it affected the SVLT immediate recall z-score both directly and via the mediation of brain hypometabolism.<br><br>Caudate dopamine depletion was directly associated with the COWAT–animal z-score, not mediated by brain hypometabolism.	NA
Chaudahary et al (2021)	Asia/India/Indigenous	26 PD-CN<br>27 PD-CI<br>27 HC	Neuroimage (MRS)	HMSE	in vivo multi-voxel proton magnetic resonance spectroscopic imaging (H-MRSI)	PD-CI<PD-CN and HC<br>Significant (post hoc $p < 0.016$) reduction in the concentration of N-acetyl aspartate (NAA) in the middle and superior frontal GMs and total choline (tCho) and total creatine (tCr) in the frontal WM<br><br>The NAA and tCr/tCho metabolite concentrations showed significant ($p < 0.05$) positive correlations with cognitive test scores in the frontal GM and WM, respectively.<br><br>The ROC analysis revealed significant ($p < 0.05$) AUC for NAA/tNAA in the frontal GM and tCho in the frontal WM.	NA

Pal et al (2018)	Asia/India/Indigenous	45 PDD<br>65 PDND<br>26 HC	Cognition (E-prime software)	MMSE, CDR, E-prime software (mrmory, executive, semantic memory, attention, psychomotor speed, visuospatitial, constructive skill, language)	NA	PDD<PDND<br>word memory, attention, psychomotor speed, visuospatial skills and executive functions<br><br>PDD=PDND<br>picture memory, semantic memory and language functions<br><br>HC=PDNC<br>working memory, attention and executive functions<br><br>PDD<HC<br>all the cognitive domains	NA
Sheelakumari et al (2016)	Asia/India/Indigenous	17 ALS<br>15 HC	Neuroimage (SWI, DTI)	NA	Visual assess for SWI<br>Iron Quantitation<br>DTI analysis	ALS<HC<br>signal-intensity grades in the posterior bank of the motor cortex bilaterally.<br>Quantitative analysis confirmed significantly higher iron content in the posterior bank of the motor cortex<br><br>ALS=HC<br>the anterior bank of the motor cortex, anterior and posterior banks of the sensory cortex, and deep nuclei.<br><br>ROC comparison showed a cutoff of 35µg Fe/g of tissue with an area under the curve of 0.78 (P = .008) for the posterior bank of the motor cortex<br><br>FA was lower in the pyramidal tracts of patients with ALS at the pons and medulla on either side, along with higher directionally averaged mean diffusivity values.<br><br>The combination of SWI and DTI revealed an AUC of 0.784 for differentiating patients with aLS from HC	NA

Huang et al (2023)	Asia/Taiwan/Indigenous	25 PDNC<br>24 PDMCI	Plasma (α-synuclein, T-tau, Aβ-42)<br>Neuroimage (DTI)	MMSE, CDR, iADL, TMT-A, TMT-B, Verbal fluency, DST, CVLT-SF, BNT, JLO	DTI (MD, RD, AD, FA)	plasma Aβ-42 ~ FA<br>middle occipital, angular, and middle temporal gyri of the left brain,<br><br>plasma T-tau ~ surface area of the isthmus or the average thickness posterior part of right cingulate gyrus.<br><br>Visuospatial and executive function ~ axial diffusivity in bilateral cingulate gyri.	NA
Chang et al (2021)	Asia/Taiwan/Indigenous	146 PD	Neuroimage (MRI)	MMSE, CASI, WAIS-III	MRI - PWMH, DWMH	Attention and memory were significantly decreased in patients with more advanced DWMH injuries.<br><br>Attention, memory, and language were significantly impaired in patients with worse PWMH lesions.	NA

Fang et al (2021)	Asia/Singapore/ indigenous	20 PD<br>35 HC	Neuroimage (MRI)	MoCA, FAB, ADL	MRI - WML segmentation	WML volume ~ cognitive dysfunctions in PD patients ($p < 0.05$), with differential impact in the frontal lobe and periventricular regions on cognitive domains ($p < 0.01$)	NA
Mak et al (2014)	Asia/Singapore/ indigenous	25 PDMCI<br>65 PDNC	Neuroimage (MRI)	MMSE, MoCA, neuropsychological battery	Quantification of GM, WM and Subcortical Deep GM Volumes. Vertex Analysis for Assessment of SDGM Shape Alterations. Cortical Thickness Analysis.	PDMCI<PDNC<br>volumes of the thalamus (P = .03) and the nucleus accumbens (P = .04).<br><br>nucleus accumbens and putamen ~ attention/working memory domains (P < .05)<br><br>nucleus accumbens ~ language domains (P = .04).<br><br>PDMCI=PDNC<br>subcortical shape or in cortical thickness.	NA

Mak et al (2014)	Asia/Singapore/ indigenous	23 PDMCI<br>67 PDNC	Neuroimage (MRI)<br>Cognition (MMSE, MoCA, NP tests)	MMSE, MoCA, neuropsychol ogical battery	Quantification of GM volume	cognition scores PDMCI < PDNC<br>(MMSE: 26.9 vs28.4, p=0.011; MoCA: 24.5 vs 27.0, p<0.001).<br><br>PDMCI < PDNC<br>executive function, attention, memory and language abilities.<br><br>PD-MCI < PDNC<br>grey matter volumes in the left insular, left superior frontal and left middle temporal areas<br><br>left insular atrophy ~ executive–attention dysfunction.	NA
Foo et al (2017)	Asia/Singapore/ indigenous	11 PDMCI<br>54 PDNC	Neuroimage (MRI)	MMSE, MoCA, neuropsychol ogical battery	Quantification of GM volume, Hippocampal subfields volumes	baseline PDMCI < PDNC<br>volumes in the left fimbria, right CA1, and right HATA<br><br>PDMCI < PDNC<br>global cognition scores<br><br>Baseline right CA1 ~ attention.<br><br>18m f/u PD-convertor<br>CA2–3 ~ episodic memory<br><br>Baseline volumes of GC-DG, right CA4, left parasubiculum, and left HATA were predictive of the conversion from PDNC to PDMCI.	NA

Study	Regions/Country/Population	Sample disease (mean age)	Biomarker (type, markers)	Cognitive measures	Brain measures	Biomarker results	Multimodal results

Chung et al (2022)	Asia/South Korea/Indigenous	75 PDMCI<br>(38 PDD-high risk, 37 PDD low-risk)	Neuroimage (WM connectivity)	SNSB, MMSE	Voxel wised tract-based spatitial statistics<br>- graph-theoretical concept to identify the subnetworks of WM connectivity	PDD-H < PDD-L<br>poorer cognitive performance on frontal/executive, visual memory/visuospatial, and attention/working memory/language function at baseline assessment.<br><br>PDD-H - more severely disrupted WM connectivity in both frontal and posterior cortical regions with 8 hub nodes in the degree-based statistic analysis.<br><br>The strength of structural connectivity within the identified subnetworks was correlated with the composite scores of frontal/executive function domain ($\gamma = 0.393$) and the risk score of PDD conversion within 5 years ($\gamma =- 0.480$).	NA
Baek et al (2020)	Asia/South Korea/Indigenous	96 ALS (probable or definite)<br>47 HC	Neuroimage (DTI)	NA	Voxel wise analysis for FA, AD, RD, MD, MO	ALS > HC had significant differences in DTI scalars in the diffuse tracts of the brain, which was predominant in the corticospinal tract at the brainstem and cerebellar peduncle area.<br><br>DTI ~ ALSFRS-R scores<br>delta ALSFRS-R score ~ disease progression.<br><br>More severe and widespread brain degeneration was observed in rapidly progressive ALS.	NA

Lee et al (2017)	Asia/South Korea/Indigenous	26 ALS<br>26 CVD<br>26 HC	Neuroimage (QSM)	NA	ROI based susceptability value - relative susceptibility	ALS > CVD, ALS > HC<br>cortical and relative susceptability value (p = 0.01, p = 0.004, p < 0.001, and p < 0.001, respectively).<br><br>ALS<CVD, ALS<HC<br>subcortical white matter mean (p = 0.04).<br><br>ROC curve - RSmean was 0.70	NA
Ahn et al (2019)	Asia/South Korea/Indigenous	27 PSP-RS<br>27 IPD<br>27 HC	Neuroimage (MRI)	NA	midbrain area, pons area, P/M ratio	PSP-RS < PD and HC<br>midbrain area ((midbrain, Pre-PSP-RS vs. PD = 1.01 cm2 vs. 1.29 cm2, p < 0.001, Pre-PSP-RS vs. controls = 1.01 cm2 vs. 1.29 cm2, p < 0.001)<br><br>PSP-RS > PD and HC<br>P/M ratio (Pre-PSP-RS vs. PD = 5.27 vs. 4.03, p < 0.001, Pre-PSP-RS vs. controls = 5.27 cm2 vs. 4.06 cm2, p < 0.001)<br><br>The P/M ratio had high sensitivity (vs. PD, 96.3%, vs. control, 88.9%) and specificity (vs. PD, 81.5%, vs. control, 96.3%) in differentiating Pre-PSP-RS patients from PD and control subjects.	NA

Yoo et al (2022)	Asia/South Korea/Indigenous	55 DLB (15 prodromal, 30 probable)<br>13 HC	Neuroimage (MRI, FP-CIT, FDG, FBB PET)	SNSB, MMSE, CDR	MRI, FP-CIT, FDG, FBB PET	Independent of amyloid deposition, caudate and putamen DAT availabilities were positively correlated with brain metabolism in the DLB-specific hypometabolic regions, most prominently in the occipital and lateral parietal cortices.<br><br>Both reduced caudate dopamine and brain hypometabolism were associated with low z-scores of RCFT copy, SVLT immediate recall and COWAT–animal.<br><br>Path analyses showed that the effect of reduced caudate dopamine on the RCFT copy z-score was completely mediated by brain hypometabolism, whereas it affected the SVLT immediate recall z-score both directly and via the mediation of brain hypometabolism.<br><br>Caudate dopamine depletion was directly associated with the COWAT–animal z-score, not mediated by brain hypometabolism.	NA
Chaudahary et al (2021)	Asia/India/Indigenous	26 PD-CN<br>27 PD-CI<br>27 HC	Neuroimage (MRS)	HMSE	in vivo multi-voxel proton magnetic resonance spectroscopic imaging (H-MRSI)	PD-CI<PD-CN and HC<br>Significant (post hoc $p < 0.016$) reduction in the concentration of N-acetyl aspartate (NAA) in the middle and superior frontal GMs and total choline (tCho) and total creatine (tCr) in the frontal WM<br><br>The NAA and tCr/tCho metabolite concentrations showed significant ($p < 0.05$) positive correlations with cognitive test scores in the frontal GM and WM, respectively.<br><br>The ROC analysis revealed significant ($p < 0.05$) AUC for NAA/tNAA in the frontal GM and tCho in the frontal WM.	NA

Pal et al (2018)	Asia/India/Indigenous	45 PDD<br>65 PDND<br>26 HC	Cognition (E-prime software)	MMSE, CDR, E-prime software (mrmory, executive, semantic memory, attention, psychomotor speed, visuospatitial, constructive skill, language)	NA	PDD<PDND<br>word memory, attention, psychomotor speed, visuospatial skills and executive functions<br><br>PDD=PDND<br>picture memory, semantic memory and language functions<br><br>HC=PDNC<br>working memory, attention and executive functions<br><br>PDD<HC<br>all the cognitive domains	NA
Sheelakumari et al (2016)	Asia/India/Indigenous	17 ALS<br>15 HC	Neuroimage (SWI, DTI)	NA	Visual assess for SWI<br>Iron Quantitation<br>DTI analysis	ALS<HC<br>signal-intensity grades in the posterior bank of the motor cortex bilaterally.<br>Quantitative analysis confirmed significantly higher iron content in the posterior bank of the motor cortex<br><br>ALS=HC<br>the anterior bank of the motor cortex, anterior and posterior banks of the sensory cortex, and deep nuclei.<br><br>ROC comparison showed a cutoff of 35µg Fe/g of tissue with an area under the curve of 0.78 (P = .008) for the posterior bank of the motor cortex<br><br>FA was lower in the pyramidal tracts of patients with ALS at the pons and medulla on either side, along with higher directionally averaged mean diffusivity values.<br><br>The combination of SWI and DTI revealed an AUC of 0.784 for differentiating patients with aLS from HC	NA

Huang et al (2023)	Asia/Taiwan/Indigenous	25 PDNC<br>24 PDMCI	Plasma (α-synuclein, T-tau, Aβ-42)<br>Neuroimage (DTI)	MMSE, CDR, iADL, TMT-A, TMT-B, Verbal fluency, DST, CVLT-SF, BNT, JLO	DTI (MD, RD, AD, FA)	plasma Aβ-42 ~ FA<br>middle occipital, angular, and middle temporal gyri of the left brain,<br><br>plasma T-tau ~ surface area of the isthmus or the average thickness posterior part of right cingulate gyrus.<br><br>Visuospatial and executive function ~ axial diffusivity in bilateral cingulate gyri.	NA
Chang et al (2021)	Asia/Taiwan/Indigenous	146 PD	Neuroimage (MRI)	MMSE, CASI, WAIS-III	MRI - PWMH, DWMH	Attention and memory were significantly decreased in patients with more advanced DWMH injuries.<br><br>Attention, memory, and language were significantly impaired in patients with worse PWMH lesions.	NA

Fang et al (2021)	Asia/Singapore/ indigenous	20 PD<br>35 HC	Neuroimage (MRI)	MoCA, FAB, ADL	MRI - WML segmentation	WML volume ~ cognitive dysfunctions in PD patients ($p < 0.05$), with differential impact in the frontal lobe and periventricular regions on cognitive domains ($p < 0.01$)	NA
Mak et al (2014)	Asia/Singapore/ indigenous	25 PDMCI<br>65 PDNC	Neuroimage (MRI)	MMSE, MoCA, neuropsychol ogical battery	Quantification of GM, WM and Subcortical Deep GM Volumes. Vertex Analysis for Assessment of SDGM Shape Alterations. Cortical Thickness Analysis.	PDMCI<PDNC<br>volumes of the thalamus (P = .03) and the nucleus accumbens (P = .04).<br><br>nucleus accumbens and putamen ~ attention/working memory domains (P < .05)<br><br>nucleus accumbens ~ language domains (P = .04).<br><br>PDMCI=PDNC<br>subcortical shape or in cortical thickness.	NA

Mak et al (2014)	Asia/Singapore/ indigenous	23 PDMCI<br>67 PDNC	Neuroimage (MRI)<br>Cognition (MMSE, MoCA, NP tests)	MMSE, MoCA, neuropsychol ogical battery	Quantification of GM volume	cognition scores PDMCI < PDNC<br>(MMSE: 26.9 vs28.4, p=0.011; MoCA: 24.5 vs 27.0, p<0.001).<br><br>PDMCI < PDNC<br>executive function, attention, memory and language abilities.<br><br>PD-MCI < PDNC<br>grey matter volumes in the left insular, left superior frontal and left middle temporal areas<br><br>left insular atrophy ~ executive–attention dysfunction.	NA
Foo et al (2017)	Asia/Singapore/ indigenous	11 PDMCI<br>54 PDNC	Neuroimage (MRI)	MMSE, MoCA, neuropsychol ogical battery	Quantification of GM volume, Hippocampal subfields volumes	baseline PDMCI < PDNC<br>volumes in the left fimbria, right CA1, and right HATA<br><br>PDMCI < PDNC<br>global cognition scores<br><br>Baseline right CA1 ~ attention.<br><br>18m f/u PD-convertor<br>CA2–3 ~ episodic memory<br><br>Baseline volumes of GC-DG, right CA4, left parasubiculum, and left HATA were predictive of the conversion from PDNC to PDMCI.	NA

Study	Regions/Country/ population	Sample disease (mean age)	Biomarker (type, markers)	Cognitive measures	Brain measures	Biomarker results	Multimodal results
Talwar et al (2017)[1]	South Asia/ India/ Indigenous	108 AD, 153 HC	*APOE* genotyping	MMSE	APOE ε4 allele	ε4 allele frequency was significantly high in AD as compared to HC ε2 was overrepresented in HC	NA
P.S. Jairani et al., (2016)[2]	South Asia/India	156 AD, 87 MCI, 127 FTD, 37 VaD, 12 DLB, 138 Controls	Genetics APOE genotyping	Addenbrooke's Cognitive Examination (ACE)	NA	APOEϵ2/ϵ2, > Controls and Vascular dementia, DLB APOEϵ3/ϵ3, AD=MCI=FTD=VD =DLB = Controls APOEϵ4/ϵ4, AD> MCI	Strong positive association was established between the APOE ε4 allele and AD. H1H1 genotype was found to have an additive effect in contributing to either disease risk in combination with the APOE e4 allele or protection in combination with the APOE e2 or e3 allele.
P M Aswathy et al., (2016)[3]	South Asia/India	94 FTD, 12 PNFA, 7 SD, 3 FTD-ALS, 130 Controls	Genetics (MAPT, GRN), Plasma GRN	NA	MRI Brain	Frequency of PGRN mutation was found to be 1% (1/116) in total FTD cases. Progranulin levels were 28 ng/mL, which is reduced up to about one third with respect to non-mutation carriers and controls	-
Gautami Das et al., (2013)[4]	South Asia/India	19 bvFTD, 3 SD, 4 PPA, 3 FTD-MND, 48 PSP, 4 CBD, 269 Controls	MAPT, PGRN, APOE, Progranulin levels,	NA	NA	Plasma Progranulin level among individuals carrying different genotypes of rs5848. Individuals with C/C genotype had the highest level of plasma Progranulin followed by those with C/T and T/T genotypes	MAPT, PGRN and APOE play limited roles in FTLD pathogenesis among Indians
Mukherjee et al., (2015)[5]	South Asia/India	62 bvFTD, 9 SD, 9 PNFA, 2 FTD-MND, 29 PSP, 20 CBD, 150 controls	Reverse PCR assay	NA	MRI brain	Absence of abnormal GGGGCC expansions in the cohort	C9orf72 hexanucleotide repeat expansion may be rare in FTLD in Indian patients
Syama A et al., (2018)[6]	South Asia/India	17 AD	Whole-exome sequencing	HMSE, NPI, CDR	MRI brain	ECE2, MMP3, PLAT, PSEN2, SORL1 deleterious variants observed APOE4 was identified in 8 cases	Rare genetic variations, primarily in genes involved in amyloid signaling, and other associated dementia-linked pathways were identified
Sonali N et al., (2012)[7]	South Asia/India	63 AD, 15 aMCI, 63 healthy control	BDNF gene polymorphism	MMSE	NA	Patients with AD and aMCI had higher serum BDNF levels than had the controls	val66met polymorphism and BDNF serum level between the three groups and genotype did not significantly affect the serum BDNF level or age, MMSE score in AD and aMCI
Luthra K et al., (2004)[8]	South Asia/India	29 AD, 25 VaD, 76 Controls	APOE4	MMSE, CDR	MRI Brain	frequency of ϵ4 allele was significantly higher among cases of AD and VaD compared with controls	The ϵ3 ϵ3 and ϵ2 ϵ3 genotypes were found to be protective. The odds of developing AD or VaD were 4.4and 3.7 times higher, respectively, in the presence of even a single ϵ4 allele.

Chandak GR et al., (2002)[9]	South Asia/India	49 AD, 100 Healthy controls	APOE e4, PSEN1	NA	NA	Low frequency of APOE E*4 allele was seen in this study, consistent with a low prevalence of AD in this study.	NA
Wang G et al., (2019)[10]	South Asia/China	169 AD	Next-generation sequencing	NA	NA	69 functional variants (including missense, frame-error, nonsense, read-through, and splice-site) in 20 dementia-causal genes	Potentially pathogenic mutations were also observed in non-AD dementia-causal genes such as GRN, SQSTM1, LRRK2, NOTCH3, and HTRA1
Gao Y et al., (2019)[11]	South Asia/China	148 Familial AD	Sanger sequencing APP, PSEN1, 2	NA	NA	Total frequency of the mutations was 8.8%	-
Jiang Bin et al., (2018)[12]	South Asia/China	15 Familial AD	Whole exome sequencing	MMSE	MRI brain	12 (80%) were found to carry missense variants in APP, PSEN1 and PSEN2	-
Han LH et al., (2020)[13]	South Asia/China	49 EOD	Next generation sequencing (NGS)	NA	NA	Seven previously reported pathogenic variants (p.I213T and p.W165C in PSEN1; p.D678N in APP; c.1349_1352del in TBK1; p.P301L and p.R406W in MAPT; p.R110C in NOTCH3)	Two novel variants of uncertain significance (p.P436L in PSEN2; c.239- 11G>A in TARDBP) were identified
Cheng HR et al., (2023)[14]	South Asia/China	29 FTD	Whole exome sequencing and RP-PCR analysis	NA	NA	Known pathogenic variants (MAPT: p.P301L; TBK1:p.I450Kfs), and 4 novel variants (MAPT : p.R406Q, p.D430H, p.A330D; GRN : c.350-2A > G) were identified.	Functional analysis showed that phosphorylated tau levels were higher in cells expressing p.R406Q and p.D430H tau than those expressing wild-type tau, especially at the Thr205, Thr231, and Ser396 phosphorylation epitopes.
Sun L et al., (2021)[15]	South Asia/China	55 AD, 14 FTD, 9 Dementia	Whole exome sequencing	MoCA, MMSE,	NA	4 reported pathogenic variants, PSEN1 c.A344G, APP c.G2149A, MAPT c.G1165A, and MAPT c.G742A, 1 reported likely pathogenic variant, namely, PSEN2 c.G100A, one novel pathogenic variant, SQSTM1 c.C671A, and three novel likely pathogenic variants, namely, ABCA7 c.C4690T, ATP13A2 c.3135delC, and NOS3 c.2897-2A > G.	21 variants with uncertain significance in PSEN2, C9orf72, NOTCH3, ABCA7, ERBB4, GRN, MPO, SETX, SORL1, NEFH, ADCM10, and SORL1, etc., were also detected in patients with AD and FTD
Zhang W et al., (2020)[16]	South Asia/China	75 AD families, 506 AD, 498 normal controls	Targeted sequencing	MMSE	NA	Higher frequency for the APOE ε4 allele was observed in the LOAD families and LOAD sporadic cases compared with normal controls	Of the 75 FAD families that non-carrying mutation of causal dementia genes, 2 EOAD families had likely pathogenic variant of PLD3 and causative mutation of LRRK2, respectively, 15 AD families had risk variants of TREM2, ABCA7, CR1, FUS, and PARK7/DJ-1.
Kim EJ et al., (2022)[17]	South Asia/ Korea	72 FTD	Whole exome sequencing and RP-PCR analysis	MMSE, GDS, UPDRS, FTLDCDR	MRI brain	Only one patient with bvFTD harboring p.G706R in the MAPT gene was seen.	13 variants of uncertain significance (VUSs) in nine FTD patients were identified. Of these VUSs, M232R of the PRNP gene, was also found in two patients with bvFTD.

Park JH et al., (2021)[18]	South Asia/ Korea	331 AD, 169 Controls	Whole genome sequencing	NA	MRI or amyloid imaging	WGS analysis identified 22,526,987 variants from an APOE ε4 carrier dataset comprised of 331 AD patients and 169 controls	Combined analysis of WGS and cAD chip data revealed that SNPs rs1890078 and rs12594991 in SORCS1 and CHD2 genes, respectively, are novel genetic variants among APOE ε4 carriers
Park JE et al., (2019)[19]	South Asia/ Korea	60 EOAD	Exome sequencing	NA	18F-Florbetaben or 18F-Flutemetamolpo sitron emission tomography (PET).	5 likely pathogenic variants (LPVs), 18 variants of uncertain significance (VUSs), and 5 SORL1 or TREM2 variants were observed in this study	Three patients had ε2/ε3 and 57 patients had ε3/ε3 APOE genotype. 52 patients underwent amyloid PET imaging and they all showed positive results
VV Giau et al., (2019)[20]	South Asia/ Korea	67 EOAD	Next generation sequencing	MMSE, CDR	18 FDG-PET	Three missense mutations in PSEN1 (T119I, G209A, and G417A) and one known variant in PSEN2 (H169N) were discovered in 6% of the cases	67 missense mutations in susceptibility genes for LOAD were identified. 70 additional novel and missense variants in other genes, such as MAPT, GRN, CSF1R, and PRNP, related to neurodegenerative diseases, were also observed.
Tan YJ et al., (2023)[21]	South Asia/ Singapore	25 Familial FTD, 35 sporadic	Next generation sequencing (whole exome, genome) and repeat-primed PCR for C9orf72 expansion, Serum NfL levels	MMSE, MoCA, FTLDCDR	MRI Brain<br>18 F-FDG PET	16/60 cases carried pathogenic or likely pathogenic variants in a FTD-related gene, including: MAPT Gln351Arg (n = 1); GRN Cys92Ter (n = 1), Ser301Ter (n = 2), c.462 + 1G > C (n = 1); C9orf72 expansion (35–70 repeats; n = 8); TREM2 Arg47Cys (n = 1); and OPTN frameshift insertion (n = 2).	Genetic mutations accounted for 48% (12/25) of patients with familial FTD, and 11.4% (4/35) of patients with sporadic FTD. C9orf72 repeat expansions were the most common genetic mutation followed by GRN variants. Within mutation carriers, plasma NfL was highest in a C9orf72 expansion carrier, and CSF NfL was highest in a GRN splice variant carrier.
Hsu JL et al., (2021)[22]	South Asia/ Singapore	52 AD, 33 FTD	Targeted panel, Plasma biomarkers- total tau, Ab42, Ab40	MMSE, MoCA	MRI Brain, Amyloid and Tau PET	Nine of 52 patients with young-onset AD had mutations: 2 (APP), 4 (PSEN1), 2 (PSEN2), and 1 (TREM2). Two of 33 patients (6.1%) with young-onset FTD had mutations in MAPT and LRRK2. Three of the 6 patients (50.0%) with possible FTD had individual mutations in APP, PSEN2, or MAPT.	Plasma level of total tau was increased and Aβ42 and Aβ40 levels decreased in all groups of dementia patients compared to controls
De Silva (2005)[23]	South Asia/Sri Lanka/	23 AD, 21 Controls	Blood/ Serum<br>(tHcy)	MMSE<br>CAMCOG	apoE genotyping<br>MTL-CT	Mean plasma tHcy higher in AD patients than controls. The frequency of apoE4 allele was significantly higher in AD patients, The mean minimum MTL thickness was significantly higher in control compared to AD	High plasma tHcy, the presence of apoE4 allele, and MTL atrophy are associated with AD
Foo JN et al (2017)[1]	South Asia	5,125 PD cases and 17,604 controls	Genome wide association study	NA	NA	Significant associations were observed at 5 loci (DLG2, SIPA1L2, STK39, VPS13C and RIT2), and same direction of associations at 9 other loci including BST1 and PARK16	Results demonstrate some differences in the genetic contribution to PD between Europeans and Asians

Tan AH et al., (2020)[2]	South Asia	499 PD	Next generation sequencing-based PD gene panel	NA	Structural MRI	Homozygous p. Leu347Pro mutations were found in five unrelated Malay patients, yielding a prevalence of 6.9% among Malays	NA
Lim JL et al., (2021)[3]	South Asia/China/Malaysia/ India	496 PD	Next generation sequencing-based PD gene panel	NA	NA	14 heterozygous GBA alleles consisting of altogether 17 missense variants (8 classified as pathogenic or likely pathogenic for PD) in 25 (5.0%) patients, with a substantially higher yield among early (< 50 years) vs. late-onset patients across all three ethnicities	Largest study on GBA variation from South-East Asia, and highlights that these populations, especially those with EOPD, would be relevant for studies including clinical trials targeting GBA pathways.
Pulkes T et al., (2011)[4]	South Asia/ Thailand	155 PD, 158 controls	APOE genotyping	NA	NA	A high frequency of the APOE-ε2 allele among patients with PD than among controls (odds ratio = 2.309, 95% confidence interval) were identified	NA
Gopalai AA et al., (2019)[5]	South Asia/ Malaysia	523 PD, 491 controls	SNP genotyping assay	NA	NA	A significant protective association for N551K was observed in Malay ancestry, with a protective trend seen for R1398H.	NA
Lin CH et al., (2008)[6]	South Asia/ Taiwan/ Singapore/China	771 PD	Genetic sequencing and PCR	NA	NA	One novel missense variant AL746Thr was identified in a single heterozygous state in three patients. The frequency of this variant was significantly higher in PD cases than controls	A rare variant of the ATP13A2 was associated with an increased risk of PD among ethnic Chinese in Asia
Tan EK et al., (2006)[7]	South Asia/ Malaysia/ India/ China	80 PD, 200 HCs	Sequence analysis of exon-intron junctions in PINK1	NA	NA	Three different mutations (two homozygous nonsense and one heterozygous missense) in the putative kinase domain were found in three patients, giving a 3.7% frequency of PINK1 mutations. Mutations were absent in healthy controls	Polymorphisms of PINK1 do not appear to modulate risk of PD in our population.
Sadhukhan D et al., (2020)[8]	South Asia/ India	412 PD, 107 PD with cognitive impairment, 107 Parkinson plus syndrome, 200 HCs	p.Gly2019Ser variant screening by polymerase chain reaction followed by restriction fragment length polymorphism analysis.	NA	NA	p.Gly2019Ser variant was identified in an East Indian young-onset female PD patient in a heterozygous state having several motor and autonomic problems without disturbed cognition.	The overall low frequency of the p.Gly2019Ser variant suggests limited role in PD in Indian Patients
Oh JH et al., (2023)[9]	South Asia/ South Korea	310 PD, 100 HCs	Whole-genome sequencing	NA	NA	30 significant locus deletions were identified in PD, GPR27 was expressed specifically in brain tissue, and GPR27 copy number loss was associated with upregulated SNCA expression and downregulated dopamine neurotransmitter pathways	These observations provide a whole-genome view of PD and suggest that small genomic deletions in regulatory domains contribute to risk of PD
Deng X et al., (2022)[10]	South Asia/ Singapore	206 PD	Genetics, Homocysteine and CRP levels	NA	NA	Cluster A (severe subtype in motor, NMS and cognitive domains), cluster B (intermediate subtype with cognitive impairment and mild NMS) and cluster C (mild subtype and young age of onset). The significantly different allele frequencies in two SNPs (Park16 rs6679073 A allele and SV2C rs246814 T allele), suggest that these may be important genetic biomarkers for PD subtypes.	Of the 3 subtypes identified amongst early PD patients, the severe subtype was associated with significantly lower frequency of Park16 and SV2C alleles and higher levels of Hcy and CRP

Samat NA et al., (2017)[11]	South Asia/ Malaysia	46 PD	ApoE genetic polymorphism, plasma synuclein	MoCA, TMT,	NA	21 patients (72.4%) with executive dysfunction were from the PD-MCI group; 17 (77.3%) with severe executive dysfunction and 4 (57.1%) had mild to moderate executive dysfunction. There were no differences in the plasma α-synuclein concentration between the presence or types of cognitive impairment based on MoCA, PDCRS, and CTMT.	The ApoEe4 allele carrier frequency was significantly higher in patients with executive dysfunction ($p = 0.014$).
Padmaja MV et al., (2022)[12]	South Asia/India	16 PD	PARK2 gene mutations	NA	NA	PARK2 mutations were present in 68% of the early onset cases. 4 PARK2 sequence variants c.1239G>C, c.171+25T>C, c.202A>G, c.601G>A, and a novel insertion mutation, c.798_799insA in the exon 7 of PARK2 gene were also observed	NA
Sanyal J et al., (2015)[13]	South Asia/India	150 PD, 150 HCs	PARKIN gene	NA	NA	Eleven nucleotide variants including two novel changes were detected. Cerebrospinal fluid (CSF) parkin protein expression of the novel mutation, Val186Ile (found in heterozygous condition in one patient only) was almost 2.7 folds lower than the controls and other PD patients	Among the ethnically defined Bengalee population of West Bengal, occurrence of Parkin mutation is 4% (6/150) of the PD patient pool supported with decreased folds of expression of CSF PARKIN protein.
Tay YW et al., (2023)[14]	South Asia/ Malaysia	161 PD	Next-generation sequencing-based PD gene panel and MLPA	NA	NA	Thirty-five patients (21.7%) carried pathogenic or likely pathogenic variants involving: GBA1, PRKN, PINK1, DJ-1, LRRK2, and ATP13A2. Pathogenic/likely pathogenic variants in GBA1 were identified in 13 patients (8.1%), and were also commonly found in PRKN and PINK1 (11/161 = 6.8% and 6/161 = 3.7%, respectively). The overall detection rate was even higher in those with familial history (48.5%) or age of diagnosis ≤40 years (34.8%).	RKN exon 7 deletion and the PINK1 p. Leu347Pro variant appear to be common among Malay patients. Many novel variants were found across the PD-related genes.
Chew EGY et al., (2018)[15]	South Asia/ China	198 PD	Whole exome sequencing	NA	NA	8 of the 43 reported risk variants were polymorphic. Several heterozygotes for rare loss-of-function mutations were identified	The 33 reported candidate genes and associated variants are unlikely to confer significant PD risk in East Asian population
Pulkes T et al., (2014)[16]	South Asia/ Thailand	480 PD,<br>395 PD	Direct sequencing of GBA	NA	NA	Heterozygous GBA mutations were seen in 24 patients and 2 controls.	GBA mutations had more rapid progressive course
Narain P et al., (2019)[17]	South Asia/ India	154 ALS, 50 healthy controls	Targeted sequencing of 25 ALS associated genes	NA	NA	Variants were identified in 5.36% of Sporadic ALS, with one novel variant each in ERBB4, SETX, DCTN1 and MATR3	Rare variants could be potentially pathogenic and functional studies are warranted to decisively establish the pathogenic mechanisms associated with them.
Vats A et al., (2017)[18]	South Asia/ India	75 ALS, 115 HCs	Repeat-primed PCR	NA	NA	Range of repeat number was from 3 to 11 in ALS patients and controls	Repeat pattern was similar to most of the Asian populations
Narain P et al., (2018)[19]	South Asia/ India	154 ALS	NGS ALS associated genes	NA	NA	Known pathogenic mutations in SOD1 (G148D; H44R), TARDBP (M337V; N267S), DAO (R199Q), and ANG (K41I). In addition, 7 potentially pathogenic missense variants not been previously reported in ALS patients were identified including 3 novel variants (OPTN: K489E, DAO: E121K, and SETX: L2163V) that are not reported in large population databases and 4 rare variants	

						(CHMP2B: E45K, SQSTM1: G262R and P438L, ERBB4: R103H)	
Arshad F et al., (2022)[20]	South Asia/ India	13 FTD-ALS	Whole exome sequencing	ACEIII, CDR, NPI	Structural and PET MRI brain	Novel loss-of-function (LoF) variant c.1810G>T(p.E604X) in the TBK1 gene. Neuroimaging showed a pattern of asymmetric frontotemporal atrophy and hypometabolism. Segregation analysis of the variation demonstrated its presence in several family members, although none of the other members was symptomatic.	
Shamim U et al., (2020)[21]	South Asia/ India	593 ALS	Repeat-primed PCR- c9orf72	NA	NA	The G4C2 expansion was observed in 3.2% (19/593) of total cases where 9/19 (47.4%) positive cases belonged to the eastern region of India.	The study establishes the prevalence of C9orf72 expansion in Indian ALS cases
Edgar S et al., (2021)[22]	South Asia/ Malaysia	101 ALS	RP-PCR assay- SOD1, FUD, TARDBP	NA	NA	Mutations were found in 5.9% (6 of 101) of patients including 3.0% (3 of 101) of patients with the previously reported SOD1 missense mutations (p.V48A and p.N87S) and 3.0% (3 of 101) of patients with the C9orf72 repeat expansion.	No mutations were found in the FUS and TARDBP genes.
Giau VV et al., (2019)[23]	South Asia/ Thailand	50 AD	Next-generation sequencing in 50 AD genes	NA	NA	Novel mutation, APP p.V604M, and the known causative variant, PSEN1 p.E184G, were found in two of the familiar cases. Remarkably, among 61 missense variants were additionally discovered from 21 genes out of 50 genes, six potential mutations including MAPT P513A, LRRK2 p.R1628P, TREM2 p.L211P, and CSF1R (p.P54Q and pL536V) may be considered to be probably/possibly pathogenic	
Kim EJ et al., (2018)[24]	South Asia/ Korea	107 FTD	Next-generation sequencing in 46 FTD, ALS genes, RP-PCR c90rf72	NA	NA	NGS revealed one known pathogenic variant (c.708+1G>A) in GRN gene patient bvFTD. In addition, a novel in-frame deletion (c.2675_2683del) in the CSF1R gene was identified in a patient with bvFTD	46 variants of uncertain significance were detected in other patients. None of the patients had expanded hexanucleotide repeats in C9orf72.
Kwon MJ et al., (2012)[25]	South Asia/ Korea	258 ALS	SOD1, FUS, TARDBP, ANG, and OPTN genes	NA	NA	The frequency of fALS was estimated to be 3.5% (9/258), and mutations were identified in 88.9% (8/9) of fALS patients but only in 2.8% (7/249) of sALS patients	NA
